# Toward sustainable food preservatives: high-level production of sorbic acid in engineered *Saccharomyces cerevisiae*

**DOI:** 10.1038/s41467-026-72163-8

**Published:** 2026-04-21

**Authors:** Jianbin Xiao, Wei Lin, Xingtong Chen, Haoyu Yu, Chao Chen, Qin Li, Fan Cai, Huaidong Zhang, Huibin Chen, Mingliang Zhang, Yongjin J. Zhou, Li Li

**Affiliations:** 1 Engineering Research Center of Industrial Microbiology (Ministry of Education), Fujian Normal University, Fuzhou, P. R. China; 2 College of Life Sciences, Fujian Normal University, Fuzhou, P. R. China; 3 Division of Biotechnology, Dalian Institute of Chemical Physics, Chinese Academy of Sciences, Dalian, China

**Keywords:** Metabolic engineering, Fungi, Enzymes

## Abstract

Sorbic acid (SA) and its salts are among the world’s most widely used and safest food preservatives, yet their industrial production still relies on fossil fuel-derived feedstocks via chemical synthesis. Here, we report bioproduction of SA through microbial fermentation by decoding its biosynthetic pathway and metabolic engineering of *Saccharomyces cerevisiae* as a chassis. Here, we identify SA and its amide derivative sorbamide (SN) from *Myrothecium* sp. FJNU6, representing identification of SA from a microbial source. Genome sequencing and heterologous expression reveal the SA/SN biosynthetic gene cluster, comprising a highly reducing polyketide synthase (SoaA), a hydrolase (SoaB), and an amidotransferase (SoaC). To enable sustainable overproduction, we reconstitute and optimize the SoaA–SoaB pathway in *S. cerevisiae* through multilevel engineering, including dynamic promoter control, acetyl-CoA/malonyl-CoA pathway enhancement, peroxisomal compartmentalization, and two-stage fed-batch fermentation. These strategies collectively enable a production titer of 1.84 g/L SA in a 50 L bioreactor. This study uncovers a microbial biosynthetic pathway for SA/SN and establishes a microbial platform for SA production, providing a foundation for developing sustainable alternatives to fossil-based manufacturing.

## Abstract (web summary)

Sorbic acid is a widely used food preservative, yet its industrial production lacks a sustainable microbial route. Here the authors identify the biosynthetic pathway of sorbic acid and engineer yeast for its efficient production.

## Introduction

Fossil fuel-based industries have operated for over a century, but the depletion of non-renewable resources—coupled with severe environmental impacts from their extraction, refining, and utilization—has become an urgent driver for transitioning to sustainable alternatives. Microbial biotechnology has emerged as a focal area of interest, fueled by the global push to replace the fossil fuel-dependent “extract-produce-discard” linear economy with a bio-based circular one^1^. A key strength of this field lies in its ability to engineer microbial cell factories that synthesize high-value commercial chemicals via biological processes, a capability that has garnered substantial attention for its potential to reshape sustainable manufacturing^2–5^.

Sorbic acid ((2*e*,4*e*)-hexa-2,4-dienoic acid, SA) and its salt potassium sorbate (KS) are widely employed as preservatives in foods, cosmetics, and pharmaceuticals, where they exert antimicrobial activity by inhibiting the growth of molds, yeasts, and select bacteria^6^. The term “sorbic acid” traces its origin to its first isolation from the unripe berries of the mountain ash tree (*Sorbus aucuparia*) by A. W. Hoffman in the 1859^7^, and then was commercially used as antimicrobial additive in the 1940s. The diverse applications and proven safety profile of SA/KS underscore their broad functional utility and fuel sustained market growth. According to a market forecast from The Business Research Company, the global market size for SA and KS reached $279.31 billion in 2024; it is further projected to expand robustly, hitting $394.81 billion by 2029 at a compound annual growth rate (CAGR) of 7.4%^8^.

**Fig. 1:**
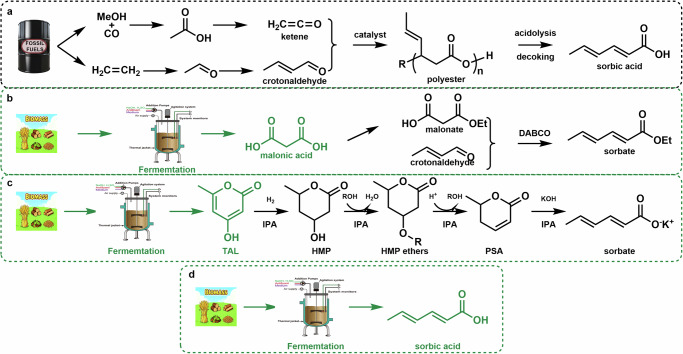
Comparison of synthesis routes to sorbic acid and sorbate. **a** Conventional industrial route using fossil-derived feedstocks (the predominant approach in current commercial production). **b** Route employing bio-based malonic acid. **c** Route utilizing bio-based triacetic acid lactone (TAL). **d** One-step fermentation process for sorbic acid production (this study). TAL: triacetic acid lactone; DABCO: 1,4-diazabicyclo [2,2,2] octane; IPA: isopropyl alcohol; HMP: 4-hydroxy-6-methyltetrahydro-2-pyrone; PSA: parasorbic acid. Chemical structures were drawn using ChemDraw Professional 17.0. Created in BioRender. Li, L. (2026) https://BioRender.com/fczlsmh.

Although SA is naturally derived from mountain ash (*S. aucuparia*) berries, the vast majority of commercial SA is produced via chemical synthesis—specifically, the reaction of ketene and crotonaldehyde, both of which are derived from fossil resources (Fig. 1a)^9^. Today, consumer demand for natural and clean-label products is rising rapidly, creating an urgent need to develop bio-production routes for SA to meet this growing preference for natural ingredients. Notably, Celanese International Corporation, the world’s leading SA manufacturer, has advanced this transition by producing bio-based KS: it uses a traditional synthesis process, but replaces conventional fossil-derived ketene and crotonaldehyde with bio-based counterparts that are chemically identical to their fossil-based equivalents^10^.

Recently, alternative synthesis routes using biomass-derived feedstocks have been developed to reduce reliance on fossil resources. In 2022, Li and colleagues explored one such approach for preservative production, employing biomass-derived malonate and crotonaldehyde as starting materials (Fig. 1b). Using the organoamine 1,4-diazabicyclo[2.2.2]octane (DABCO) as a catalyst, they achieved the selective synthesis of sorbate via a one-step condensation reaction of malonate and crotonaldehyde, with a yield of 75%^11^.

Triacetic acid lactone (4-hydroxy-6-methyl-2-pyrone, TAL) serves as a versatile bioprivileged building block for the organic synthesis of diverse high-value chemicals^12^. To date, TAL has been successfully produced using various microbial hosts, including *Escherichia coli*^13^, *S. cerevisiae*^14^, *Yarrowia lipolytica*^15^, *Pichia pastoris*^16^, *Rhodotorula toruloides*^17^. Most recently, the Huber group developed a method to produce food-grade KS using TAL as a precursor; this process employs food-grade solvents, ethanol and isopropyl alcohol, and achieves a yield of 84% (Fig. 1c)^18^.

While the aforementioned strategies (Fig. 1b, c) enable the production of bio-based SA/KS, they still rely on multiple subsequent chemical synthesis steps. Critically, KS is classified as a “synthetic” compound under current regulatory frameworks—this classification precludes its use as an active ingredient for controlling or suppressing plant diseases and pests in both field and greenhouse settings. Thus, achieving the full bioproduction of SA/KS remains a high-priority goal, as it would directly expand the scope of their applications.

In our natural products (NPs) isolation program guided by antimicrobial activity screening, we identify SA and (2*e*,4*e*)-hexa-2,4-dienamide (sorbamide, SN) from *Myrothecium* sp. FJNU6. Here, we identify the biosynthetic gene cluster responsible for SA and SN biosynthesis, and engineer *Saccharomyces cerevisiae* to produce SA via microbial fermentation (Fig. 1d). This work demonstrates de novo SA bio-production through microbial fermentation—thus laying a critical foundation for the biotechnological manufacturing of this high-value food preservative.

## Results

### Characterization and bioactivity analysis of microbial SA and SN

**Fig. 2:**
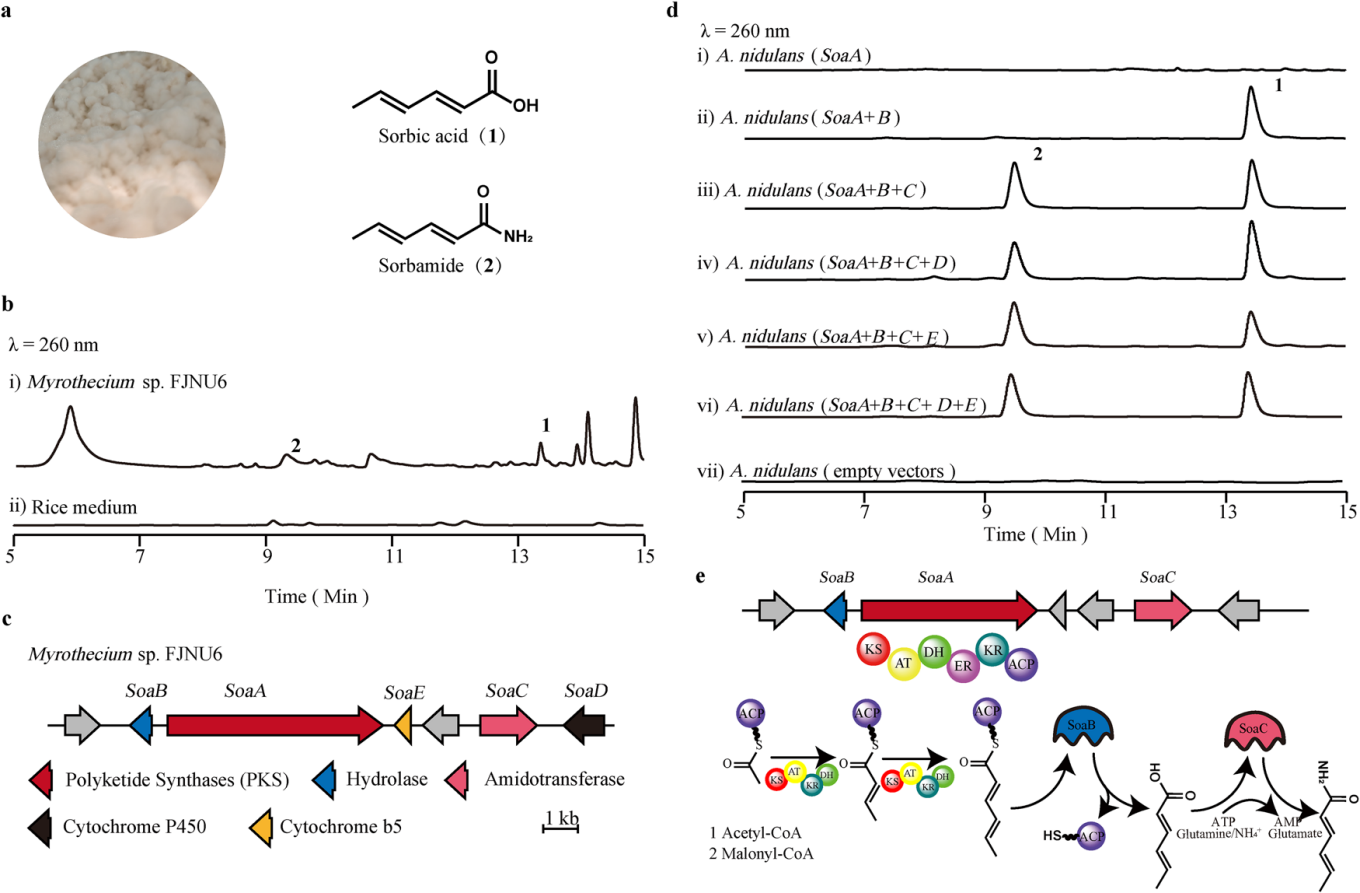
Production of sorbic acid (SA) and sorbamide (SN) by *Myrothecium* sp. and functional characterization of the *soa* gene cluster. **a** Left: Mycelial morphology of *Myrothecium* sp. FJNU6 grown in rice medium. Right: Chemical structures of SA (**1**) and SN (**2**). **b** HPLC analysis (λ = 260 nm) of metabolites from (i) *Myrothecium* sp. FJNU6 and (ii) rice medium (negative control), with peaks 1 and 2 corresponding to SA and SN, respectively. **c** Schematic of the *soa* gene cluster from *Myrothecium* sp. FJNU6, with genes color-coded by predicted function: polyketide synthases (PKS, red), hydrolase (blue), amidotransferase (pink), cytochrome P450 (black), and cytochrome b5 (yellow). The scale bar represents 1 kb. **d** HPLC analysis (λ = 260 nm) of *A. nidulans* strains expressing different combinations of *soa* genes: (i) *soaA* alone, (ii) *soaAB*, (iii) *soaABC*, (iv) *soaABCD*, (v) *soaABCE*, (vi) *soaABCDE*, and (vii) empty vectors (negative control). **e** Schematic diagram of the biosynthetic mechanism of SoaA, SoaB and SoaC. Peak 1 and 2 corresponding to SA and SN, respectively. Panels (**c**) and (**e**) were prepared using Adobe Illustrator 2021.

To isolate antifungal NPs from filamentous fungi, we first evaluated the ethyl acetate extract of *Myrothecium* sp. FJNU6 fermented on rice medium, which exhibits anti-*Candida* activity (Supplementary Fig. 1). Through a combination of extraction, fractionation, and chromatographic separation, we successfully purified 5 mg of SA and 2 mg of SN from 10 L of this rice-based fermentation medium (Fig. 2a, b). The structures of SA and SN were elucidated via 1D and 2D nuclear magnetic resonance (NMR) spectroscopy (Supplementary Figs. 2–13, Supplementary Tables 1 and 2). This represents the first report of SA and SN isolation from a microbial source, though SA had only been identified with a low level in *S. aucuparia* and several other plant species, highlighting the highly restricted natural occurrence of these compounds^19,20^.

SN was first isolated alongside SA from berry by Hoffman^7^. However, its natural occurrence has since been reported only sporadically, and its biological function remains largely uncharacterized. In our antimicrobial bioassays, SN showed no detectable antifungal activity (Supplementary Table 3, Supplementary Fig. 14), a result consistent with the well-established model that the antimicrobial efficacy of SA and its salts stem from their undissociated acid molecules.

Notably, the stark difference in bioactivity between SA (antifungal) and SN (non-antifungal) toward the producing strain (*Myrothecium* sp. FJNU6) suggests that amidation of SA (to form SN) may act as a self-protection mechanism for the microorganism. This would enable the producer to synthesize SA (for potential ecological competition) while avoiding self-toxicity via conversion to the inactive SN.

### Biosynthetic pathway of SA and SN

Research on the biosynthesis of SA remains limited. As early as 1968, Crombie and Firth investigated SA biosynthesis in *S. aucuparia* by administering [¹⁴C]-labeled precursors and analyzing the radiocarbon distribution within SA’s carbon skeleton^21^. Their findings—specifically, the efficient incorporation of labeled acetate and malonate into SA—provided key evidence that a putative polyketide synthase (PKS)-dependent pathway serves as the primary biosynthetic route for SA.

To investigate the biosynthetic pathway of SA and SN in *Myrothecium* sp. FJNU6, we first sequenced the genome of this strain and analyzed it using the online tool AntiSMASH (v.8.0)^22^. Guided by the chemical structures of SA and SN, we proposed two key hypotheses for their biosynthesis: (1) SA is synthesized via the condensation of one acetyl-CoA and two malonyl-CoA molecules, catalyzed by a highly reducing polyketide synthase (HR-PKS); and (2) the biosynthetic gene cluster for SN should contain an amidotransferase, an enzyme required for forming the amide bond in SN.

Bioinformatic analysis of the *Myrothecium* sp. FJNU6 genome identified 32 putative biosynthetic gene clusters (BGCs), including 20 polyketide synthase (PKS) BGCs (Supplementary Fig. 15). These PKS clusters were systematically evaluated based on (i) the presence of an HR-PKS capable of iterative chain elongation and programmed reduction/dehydration steps, (ii) the presence of a gene encoding a putative hydrolase or product-release enzyme, and (iii) the co-localization of an amidotransferase candidate capable of catalyzing SA amidation. Among the 20 PKS-containing BGCs, only BGC11.6 satisfied all three criteria and was therefore designated the *soa* cluster. This cluster contains three core genes: a HR-PKS gene (*soaA*), a hydrolase gene (*soaB*), and an amidotransferase gene (*soaC*). SoaA harbors the canonical functional domains of HR-PKSs: ketosynthase (KS), acyltransferase (AT), dehydratase (DH), enoyl-reductase (ER), keto-reductase (KR), and acyl carrier protein (ACP). Most HR-PKSs lack integral off-loading domains downstream of their ACP domains; instead, they either generate lactones via intramolecular lactonization of polyketide chain products or rely on trans-acting hydrolytic enzymes to facilitate polyketide release^23–25^. We hypothesize that SoaB, as a hydrolase, may act synergistically with SoaA to release SA as an unsaturated carboxylic acid. Meanwhile, SoaC, an amidotransferase, is predicted to mediate amide bond formation in SN. Additionally, there are a cytochrome P450 gene and a cytochrome b5 gene located in the *soa* (Fig. 2c).

To validate the function of the *soa* cluster, we heterologously expressed its genes in *Aspergillus nidulans* LO8030 and analyzed the products of different gene combinations. Expression of *soaA* alone yielded no detectable new peaks in the LC–MS profiles, consistent with the absence of an integral offloading domain in SoaA. In contrast, co-expression of *soaA* and *soaB* resulted in SA production. When all three core genes (*soaA*, *soaB*, and *soaC*) were co-expressed, both SA and SN were detected (Fig. 2d). Additionally, we tested the putative cytochrome P450 and cytochrome b5 genes adjacent to the *soa* cluster, but their expression did not generate new products (Fig. 2d).

These results confirm that the *soa* cluster encodes the complete biosynthetic machinery for SA and SN, representing identification and functional characterization of a SA/SN biosynthetic gene cluster. Under solid fermentation conditions, the titers of SA and SN in engineered *A. nidulans* reached 0.81 mg/L and 0.50 mg/L, respectively.

Based on the heterologous expression results, we propose the following biosynthetic pathway for SA and SN: SoaA is responsible for assembling the core scaffold of both compounds. SoaB then mediates the hydrolysis of the thioester bond linking the nascent polyketide chain to the thiol group of the holo-ACP domain in SoaA, thereby releasing free SA (Fig. 2e). For SN biosynthesis, SoaC catalyzes the formation of an amide bond between SA and an as-yet-unidentified amide donor, resulting in SN production. Notably, the lack of activity of the cytochrome P450 and cytochrome b5 genes in our heterologous expression system aligns with our observation that SA and SN are the primary (if not sole) related metabolites produced by *Myrothecium* sp. FJNU6, with no other derivative compounds detected.

### Biochemical characterization of the *soa* pathway enzymes

To provide direct biochemical evidence for the roles of SoaA, SoaB, and SoaC in SA/SN biosynthesis, we performed in vitro enzymatic assays.

**Fig. 3:**
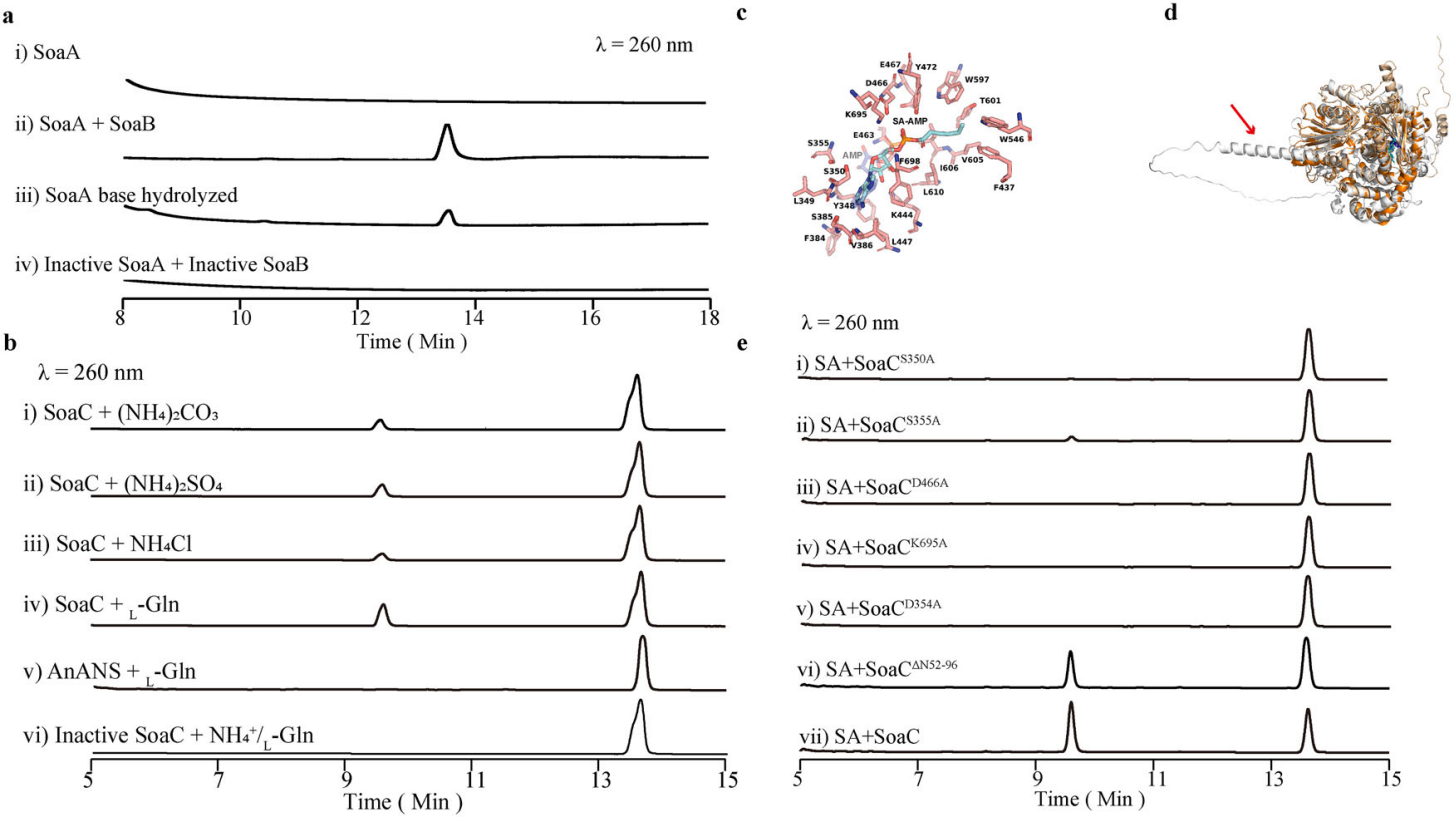
Characterization of SoaA/SoaB/SoaC activity in vitro. **a** HPLC analysis (λ = 260 nm) analysis of in vitro reactions involving SoaA and SoaB: (i) SoaA, (ii) SoaA + SoaB, (iii) SoaA reaction subjected to alkaline hydrolysis, and (iv) inactive SoaA + inactive SoaB. **b** HPLC analysis (λ = 260 nm) of reactions involving SoaC with different nitrogen sources: (i) (NH₄)₂CO₃, (ii) (NH₄)₂SO₄, (iii) NH₄Cl, (iv) *l*-Gln, (v) AnANS with *l*-Gln, and (vi) inactive SoaC with NH₄Cl/(NH₄)₂SO₄/(NH₄)₂CO₃/*l*-Gln. **c** Close-up view of the SoaC active site with key residues (yellow) and substrate/intermediate (colored sticks) showing distances (Å) between interacting moieties. **d** Overall structure of SoaC; the red arrow indicates the N-terminal region (disordered in the crystal structure). **e** HPLC analysis of reactions with SoaC mutants: (i) SoaC^S350A^, (ii) SoaC^S355A^, (iii) SoaC^D466A^, (iv) SoaC^K695A^, (v) SoaC^D354A^, (vi) SoaC^ΔN52-96^, and (vii) wild-type SoaC, all with SA as the substrate. FDAA: N-(2-furoyl) dansylalanine. Data are presented as mean values ± SD (*n* = 3 biologically independent samples).

To investigate the polyketide assembly steps of SA biosynthesis, SoaA and SoaB were characterized in vitro. His-tagged SoaA and SoaB were individually expressed in *S. cerevisiae* BJ5464-NpgA and purified to homogeneity (Supplementary Fig. 16a, b). Incubation of purified SoaA with acetyl-CoA, malonyl-CoA and NADPH did not yield a detectable free product by LC–MS (Fig. 3a, trace i). In contrast, alkaline hydrolysis of the reaction mixture released SA as the sole detectable product (Fig. 3a, trace iii), consistent with formation of an ACP-bound intermediate. Notably, the hydrolyzed product corresponded to SA derived from a triketide intermediate, with no longer-chain products detected, indicating that chain termination occurs at the triketide stage.

When SoaB was included in the reaction, SA was directly detected without chemical treatment (Fig. 3a, trace ii), indicating enzymatic product release. No product formation was observed in control reactions containing inactive SoaA and SoaB (Fig. 3a, trace iv). Together, these results indicate that SoaA catalyzes iterative polyketide chain assembly to generate a triketide ACP-tethered intermediate, while SoaB facilitates product offloading.

Sequence analysis classified SoaC as a type B asparagine synthase (AS-B)—enzymes that catalyze the ATP-dependent conversion of aspartate to asparagine using glutamine or ammonium as the nitrogen source. Like canonical AS-Bs, SoaC comprises an N-terminal glutaminase domain and a C-terminal synthetase domain, responsible for ammonia generation and ATP-dependent amidation, respectively^26^.

To characterize SoaC’s function, His-tagged SoaC was expressed in *E. coli* (Supplementary Fig. 16c) and assayed in vitro. SoaC converted SA to SN using either ammonium or glutamine as the amide donor in an ATP-dependent manner (Fig. 3b, traces i–iv) with a Km of 0.538 mM for SA (Supplementary Fig. 16d).

In contrast, the endogenous AS-B from *A. nidulans* (AnANS, ANIA_09317) showed no SA-to-SN activity (Fig. 3b, v), and heterologous expression of the *soa* cluster lacking soaC in *A. nidulans* failed to yield SN, confirming that AnANS cannot substitute for SoaC. Conversely, SoaC did not catalyze the conversion of aspartate to asparagine (Supplementary Fig. 16e), establishing it as a dedicated sorbamide synthetase.

The co-production of SN further suggests that amidation of SA is a genetically encoded modification rather than a shunt reaction. Given the weak-acid nature of SA, such modification may modulate intracellular SA levels by masking the carboxyl group. However, whether SN represents a terminal metabolite, storage form, or regulatory intermediate in vivo remains unclear.

Structural prediction by AlphaFold 3.0 and superposition with *E. coli* AsnB (PDB 1CT9) and TsnB9 (PDB 7YLZ) revealed a more enclosed hydrophobic pocket in SoaC relative to AsnB, which exposes AMP on the surface (Supplementary Fig. 17a, b), Sequence alignment showed conservation of key catalytic residues between AsnB and SoaC—K695 (ATP binding/activation), E463/D466/E467 (substrate binding and selectivity), and 350SGGIDS355 (pyrophosphate coupling)—indicating that SoaC adopts a catalytic mechanism consistent with canonical AS-B enzymes.

Molecular docking of the putative intermediate SA-AMP identified residues within 4 Å of the active site (Fig. 3c, Supplementary Fig. 18). Alanine substitutions at K695, D466, S350, D354, and S355 each abolished or markedly reduced activity (Fig. 3e, i–v), validating their catalytic roles. AlphaFold modeling further revealed an exposed N-terminal region (residues 52–96) (Fig. 3d); deletion of this segment did not affect activity (Fig. 3e, vi), confirming its dispensability for catalysis.

Together, these in vitro, in vivo, and structural analyses demonstrate that SoaC is a specialized sorbamide synthetase with a hydrophobic substrate pocket enabling SA amidation. Its unique amidotransferase activity likely provides a self-protection mechanism for *Myrothecium sp*. FJNU6 against its own SA metabolite.

### Engineering production of SA in *S. cerevisiae*

It is challenging in overproduction of commercially valuable antimicrobial agent SA in its natural host *Myrothecium* sp. FJNU6, since SoaC efficiently driven the bioconversion of SA toward SN biosynthesis. Thus, heterologous production in alternative hosts, including *S. cerevisiae*, represents a promising strategy for achieving high SA titers^27^. Therefore, we selected *S. cerevisiae* as the engineering chassis, given its well-established genetic toolbox, robustness in fermentation, and GRAS status for food-related applications. A key challenge, however, is that *S. cerevisiae* is sensitive to the antifungal activity of SA, initially suggesting high-titer SA production in this yeast might be unfeasible.

Notably, the antifungal activity of SA against *S. cerevisiae* is dictated by its dissociation state: at pH > 6.0, a condition that favors the dissociated form of SA, this antifungal activity is substantially attenuated. To quantify this effect, we evaluated the growth of *S. cerevisiae* BJ5464-NpgA across a range of pH conditions and determined the IC₅₀ of SA toward this strain. *S. cerevisiae* BJ5464-NpgA exhibited robust growth when cultured in media buffered to pH 6.0 or 7.0; correspondingly, the IC₅₀ values of SA against this strain at these two pH levels were 36.7 mM and 90.1 mM, respectively (Supplementary Fig. 19a). Furthermore, *S. cerevisiae* still maintains favorable growth at pH 7.0 (Supplementary Fig. 19b). Guided by these findings, we sought to develop a high-performance *S. cerevisiae* platform that capable of supporting efficient de novo SA biosynthesis.

### Promoter selection dictates SA production dynamics

**Fig. 4:**
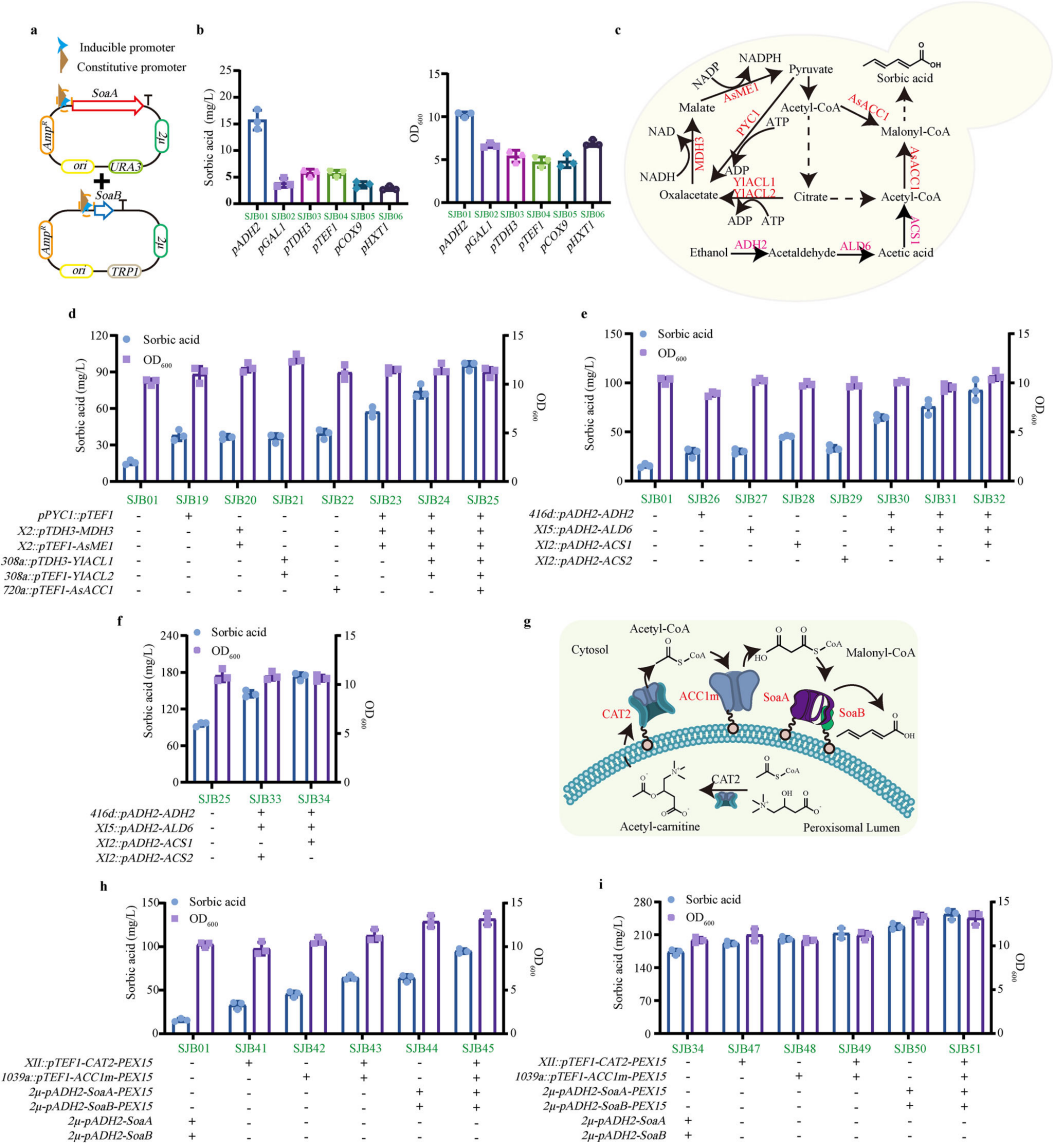
Engineering of metabolic pathways and regulatory elements for enhanced sorbic acid (SA) production. **a** Schematic of genetic constructs with inducible and constitutive promoters driving the expression of *soaA* and *soaB* genes. **b** Bar graphs showing SA titers (left y-axis) and cell density (OD₆₀₀, right y-axis) in strains with different promoter combinations for *soaA* and *soaB* genes expression. **c** Metabolic pathway illustration highlighting key enzymes and cofactors involved in SA biosynthesis, with connections to central carbon metabolism. **d** SA titers and cell density in *S. cerevisiae* strains with overexpression of genes related to acetyl-CoA pool. **e** SA production and cell growth in strains engineered with overexpression of *ADH2*, *ALD6*, *ACS1*, *ACS2*, and the combinations. **f** SA titers and cell density in strains of JB25 with overexpression of *ADH2-ALD6-ACS1* and *ADH2-ALD6-ACS2*. **g** Schematic depicting subcellular localization of key enzymes (e.g., CAT2, ACC1, SoaA, SoaB) and transport processes involved in SA biosynthesis, with focus on interactions at the peroxisomal membrane. **h** SA production and cell growth in strains with overexpression of *CAT2*, *ACC1*, SoaA and SoaB. **i** SA titers and cell density in strains the engineered strains of sJB34 with overexpression of CAT2, ACC1m, and SoaA and SoaB. In all bar graphs, blue bars represent SA titer and purple bars represent cell density (OD₆₀₀). Data are presented as mean ± s.d. (*n* = 3 biologically independent samples). Source data are provided as a Source data file. Panels (**a**), (**c**) and (**g**) were prepared using Adobe Illustrator 2021.

Promoter choice is critical for decoupling heterologous pathway expression from native regulatory networks and enabling precise metabolic control^28^. To optimize SA production, we evaluated a panel of promoters, including inducible promoters (*pADH2*, *pGAL1*, *pHXT1*) and constitutive promoters (*pTDH3*, *pTEF1*, *pCOX9*) (Fig. 4a). Among these, strains with *pADH2*-driven expression achieved both the highest cell density (OD₆₀₀ = 10.2; Fig. 4b) and the highest SA titer (15.7 mg/L; Fig. 4b). In contrast, the strain harboring the glucose‑regulated promoter *pHXT1* reached a relatively high cell density (OD₆₀₀ = 6.9) but produced only 2.88 mg/L SA. Although the *pGAL1* strain (SJB02) grown on glucose as the sole carbon source exhibited a higher cell density than the *pADH2* strain (SJB01), no detectable SA production was observed. In contrast, SA accumulation was detected in both the glucose–galactose mixed-carbon cultures and the staged induction group; however, the SA titer of the *pADH2* strain (SJB01) consistently remained higher than that of the *pGAL1* strain (SJB02) (Supplementary Fig. 20a–d). These results suggest that strong constitutive expression imposes a metabolic burden, leading to growth inhibition, whereas *pADH2*, whose activity is repressed during glucose consumption and induced upon ethanol accumulation, alleviates growth-phase toxicity by delaying pathway expression.

We also integrated the *soaA* and *soaB* under the control of different promoters into the yeast genome. Genomic integration yielded lower titers and biomass compared to episomal expression: *pTDH3-*driven strains produced 2.8 mg/L SA at OD₆₀₀ = 4.0, *pTEF1*-driven strains produced 3.1 mg/L at OD₆₀₀ = 3.9, and *pADH2*-driven strains produced 8.3 mg/L at OD₆₀₀ = 9.3 (Supplementary Fig. 20e, f). The reduced performance is likely attributed to lower gene copy number following genomic integration. Additionally, the limited biomass accumulation associated with integrated strains constrained subsequent CRISPR–Cas9 editing. Based on these findings, we adopted the 2μ episomal plasmid system as the preferred expression platform for subsequent engineering efforts.

Peptide linkers have been reported to enhance enzymatic cascades by promoting substrate channeling^29^. To test whether proximity between SoaA and SoaB could improve SA biosynthesis, we constructed fusion variants using eight rigid and flexible linkers in *S. cerevisiae*. However, all fusion constructs resulted in reduced SA production compared with the unfused control (Supplementary Fig. 21; Supplementary Table 4), with shorter linkers generally exerting stronger negative effects. The lack of improvement likely reflects intrinsic features of SoaA, a large multidomain PKS that may require structural flexibility and proper oligomerization. Forced fusion and the increased transcript size may therefore compromise expression or catalytic performance.

### Redirecting carbon flux by expanding the cytosolic acetyl-CoA pool

Cytosolic acetyl-CoA is a central metabolic precursor in *S. cerevisiae* and is a well-documented bottleneck for the biosynthesis of fatty acid and polyketide biosynthesis^30,31^. To enhance SA production, we investigated multiple strategies to boost the supply of both acetyl-CoA and its downstream derivative malonyl-CoA—two key precursors for SA biosynthesis (Fig. 4c).

Overexpression of native pyruvate carboxylase (*PYC1*, strain SJB19), co-expression of malate dehydrogenase, peroxisomal (*MDH3*) and malic enzyme^32–34^ (from *Aurantiochytrium, AsME1*) which created SJB20, heterologous ATP citrate lyase from *Yarrowia lipolytica*^35^ (*ACL1/ACL2*, SJB21), and *Aurantiochytrium* acetyl-CoA carboxylase (*AsACC1*, SJB22), each improved titers relative to the control strain (15.7 mg/L). Each modification improved SA titers, reaching 38.0, 36.7, 35.8, and 39.1 mg/L, respectively, with modestly increased cell densities (OD₆₀₀ = 11.0–12.6) compared to the control (OD₆₀₀ = 10.2; Fig. 4d).

To assess the cumulative effect of these precursor-enhancing strategies, we sequentially stacked the modifications: Integration of *AsME1* and *MDH3* into SJB19 yielded strain SJB23, producing 57.2 mg/L SA. Subsequent introduction of *ACL1/ACL2* (SJB24) further increased titers to 74.1 mg/L, and final addition of *AsACC1* (SJB25) achieved 95.2 ± 3.5 mg/L—representing a sixfold improvement over the parental control strain (Fig. 4d). These results demonstrate that coordinated reinforcement of acetyl-CoA and malonyl-CoA supply effectively drives carbon flux toward SA biosynthesis.

Since SA synthesis directly competes with the endogenous fatty acid synthase (FAS) pathway for acetyl-CoA/mlonyl-CoA, we next attempted to redirect flux away from lipid metabolism. We implemented two modifications. Placing *FAA1* (a key fatty acid activation gene) under the glucose-responsive promoter *pHXT1* (Supplementary Fig. 22a), which enables *FAA1* expression during the glucose-rich growth phase but suppresses it upon glucose depletion^36^; Expressing FAS (the core fatty acid synthesis enzyme) under the weak promoter *pBGL2*^37^ to limit acetyl-CoA diversion into fatty acid biosynthesis. Although these modifications reduced total fatty acid content, SA titers remained unchanged (Supplementary Fig. 22b, c), indicating that precursor redirection requires absolute expansion of acetyl-CoA pools rather than simple suppression of competing pathways.

### Engineering ethanol utilization for enhanced acetyl-CoA and NADPH supply

In *S. cerevisiae*, cytosolic acetyl-CoA can be synthesized through ethanol oxidation, where ethanol is sequentially converted by alcohol dehydrogenase (ADH), acetaldehyde dehydrogenase (ALD), and acetyl-CoA synthetase (ACS). Prior studies have demonstrated that overexpressing this pathway can substantially increase intracellular acetyl-CoA levels^38^. To channel ethanol-derived carbon toward SA biosynthesis, we overexpressed *ADH2*, *ALD6*, *ACS1*, and *ACS2* under the ethanol-inducible promoter *pADH2* (Fig. 4e). Individual overexpression of *ADH2* (SJB26), *ALD6* (SJB27), *ACS1* (SJB28), or *ACS2* (SJB29) increased titers to 28.7, 30.1, 45.5, and 33.4 mg/L, representing 1.8- to 2.9-fold improvements in compared to the parental strain (15.7 mg/L). Cell growth remained comparable to the control, except for *ADH2* overexpression (OD₆₀₀ = 8.9).

Combinatorial expression further reinforced flux into acetyl-CoA. Strains co-expressing *ADH2* and *ALD6* (SJB30), *ADH2* + *ALD6* + *ACS2* (SJB31), and *ADH2* + *ALD6* + *ACS1* (SJB32) produced 64.7, 75.7, and 92.4 mg/L SA, corresponding to 3.1-, 3.8-, and 4.9-fold increases compared to the control (Fig. 4e). When these modules were integrated into the high-performing acetyl-CoA-boosted strain SJB25, titers reached 144.8 mg/L (SJB33, *ADH2* + *ALD6* + *ACS2*) and 173.2 mg/L (SJB34, *ADH2* + *ALD6* + *ACS1*; Fig. 4f). The superior performance of *ACS1* relative to *ACS2* aligns with its higher affinity for acetate^39^. Based on these results, SJB34 was selected as the parent strain for subsequent engineering. Given that ALD6 also contributes to cytosolic NADPH regeneration, we next examined whether enhanced redox supply might influence SA production.

SA biosynthesis requires the KR domain of SoaA to reduce malonyl-CoA intermediates using NADPH (Supplementary Fig. 23a). Enzymes (Zwf1, Gnd1, Tkl1, Tal1) in the pentose phosphate pathway (PPP) in *S. cerevisiae* (Supplementary Fig. 23b) are involved in NADPH generation. Enhancing the expression of these enzymes has the potential to increase NADPH levels^33^. However, overexpression of *ZWF1* (SJB37), encoding glucose-6-phosphate dehydrogenase, did not increase SA levels (Supplementary Fig. 23c). Similarly, overexpression of *AsTAL1*, *AsTKL1*, or *GND1* failed to improve titers (Supplementary Fig. 23c). To confirm whether these modifications altered intracellular redox balance, NADPH levels were quantified in strains overexpressing ALD6 (SJB27), ZWF1 (SJB37), PGI1 (SJB38), and AsTAL1/AsTKL1 (SJB39). As shown in Supplementary Fig. 23d, NADPH levels were transiently elevated, particularly at 24 h, whereas by 48 h they remained only slightly higher than those of the control strain. Only *PGI1* expression under *pCOX9* yielded a modest increase (16.3 vs. 15.7 mg/L) at the expense of growth (OD₆₀₀ = 9.3). These results indicate endogenous NADPH supply through the PPP is sufficient to meet this reducing power demand, since SA biosynthesis requires only two NADPH, while fatty acid synthesis demands large amounts of NADPH (14 molecules per palmitate^40,41^).

### Peroxisomal targeting substantially boosts SA titers

Peroxisomes have emerged as promising organelles for hosting toxic compound biosynthesis owing to their high protein-folding capacity, permeability to small molecules, and lack of adverse impact on host viability when engineered proteins are targeted to their lumen^42–47^. Localizing pathway enzymes to the peroxisome not only benefits from enzyme proximity effects but also enables direct utilization of acetyl-CoA exported as acetylcarnitine^48^. Anchoring *CAT2* to the peroxisomal membrane has been shown to intercept acetylcarnitine transport into mitochondria, thereby increasing cytosolic acetyl-CoA^48,49^. Moreover, malonyl-CoA, a critical precursor for polyketide biosynthesis^50^, can be enhanced by expressing the deregulated variant *ACC1*^S1157A^, which prevents enzyme inactivation upon glucose depletion^51^. As SA biosynthesis requires one acetyl-CoA and two malonyl-CoA molecules, we hypothesized that peroxisome-based co-localization of *CAT2*, *ACC1*^S1157A^, and the SA synthases could enhance pathway flux (Fig. 4g).

To test this, we fused *CAT2* and *ACC1m* (*ACC1*^S1157A^) with the anchoring sequence PEX15 and integrated them into the *S. cerevisiae* genome. Strain SJB41 (*CAT2-PEX15*) increased titers from 15.7 to 32.8 mg/L, while SJB42 (*ACC1*^S1157A^-PEX15) reached 45.5 mg/L. Co-integration of both (SJB43) further raised titers to 64.4 mg/L, a 4.1-fold improvement, accompanied by improved growth (OD_600_ = 11.3). In parallel, peroxisome-targeted expression of *SoaA* and *SoaB* from a 2 µ plasmid boosted titers to 63.9 mg/L (SJB44). Combining both strategies—*CAT2-PEX15*, *ACC1*^S1157A^-*PEX15* integration, and peroxisome-targeted *SoaA/B* overexpression—yielded 94.8 mg/L SA (SJB45), representing a 6-fold improvement over the parental strain (Fig. 4h).

Building upon the high-producing strain SJB34, we next tested peroxisomal anchoring. Integration of *CAT2-PEX15* (SJB47) and *ACC1*^S1157A^-PEX15 (SJB48) increased titers to 191.7 and 201.3 mg/L, respectively. Co-expression of both (SJB49) raised titers to 212.9 mg/L. Further introduction of peroxisome-targeted *SoaA/B* (SJB50) resulted in 227.6 mg/L, a 31.5% increase over SJB34 with improved growth (OD_600_ = 13.2). Finally, in SJB51, where both anchoring constructs were integrated alongside 2µ-based peroxisomal *SoaA/B* overexpression, SA titers peaked at 253.3 mg/L—15-fold higher than SJB01 strain (Fig. 4i). These results demonstrate that peroxisomal surface display significantly enhances SA biosynthesis by possible improving enzyme co-localization, reducing substrate diffusion losses, and increasing utilization of peroxisome-derived acetyl-CoA pools.

### Two-stage fed-batch fermentation enables high-level SA production

Promoter screening identified *pADH2* as the most efficient driver for SA biosynthesis. Since *pADH2* is strongly repressed by glucose but robustly induced by ethanol, shake-flask cultures could not simultaneously achieve high biomass and high titers. To overcome this, we evaluated the effect of ethanol induction in 50 L fed-batch fermentations of the engineered strain SJB51.

During the initial phase, glucose feeding was maintained to keep residual concentrations below 1.5 g/L. This strategy supported rapid biomass accumulation (OD₆₀₀ ≈ 81 at 37 h) and concurrent ethanol production (up to 3.6 g/L). At this stage, only trace amounts of SA were detected (15.8 mg/L), reflecting strong glucose repression of the pathway. Once glucose supplementation ceased, ethanol was gradually consumed and SA began to accumulate, reaching 387.8 mg/L at 64.5 h. However, as ethanol was depleted (<0.1 g/L), SA synthesis slowed, and titers declined from a maximum of 436 mg/L at 100 h to 410 mg/L at 146 h (Supplementary Fig. 24a), indicating insufficient carbon flux toward SA.

To address this limitation, we implemented ethanol feeding after glucose depletion, maintaining residual ethanol below 1 g/L. Under these conditions, biomass stabilized at OD₆₀₀ ≈ 93–99, while SA titers steadily increased, peaking at 992.7 mg/L at 132 h (Supplementary Fig. 24b). These results highlight the essential role of ethanol not only as a carbon source for acetyl-CoA supply but also as a transcriptional activator of *pADH2*, ensuring strong pathway expression during the production phase.

**Fig. 5:**
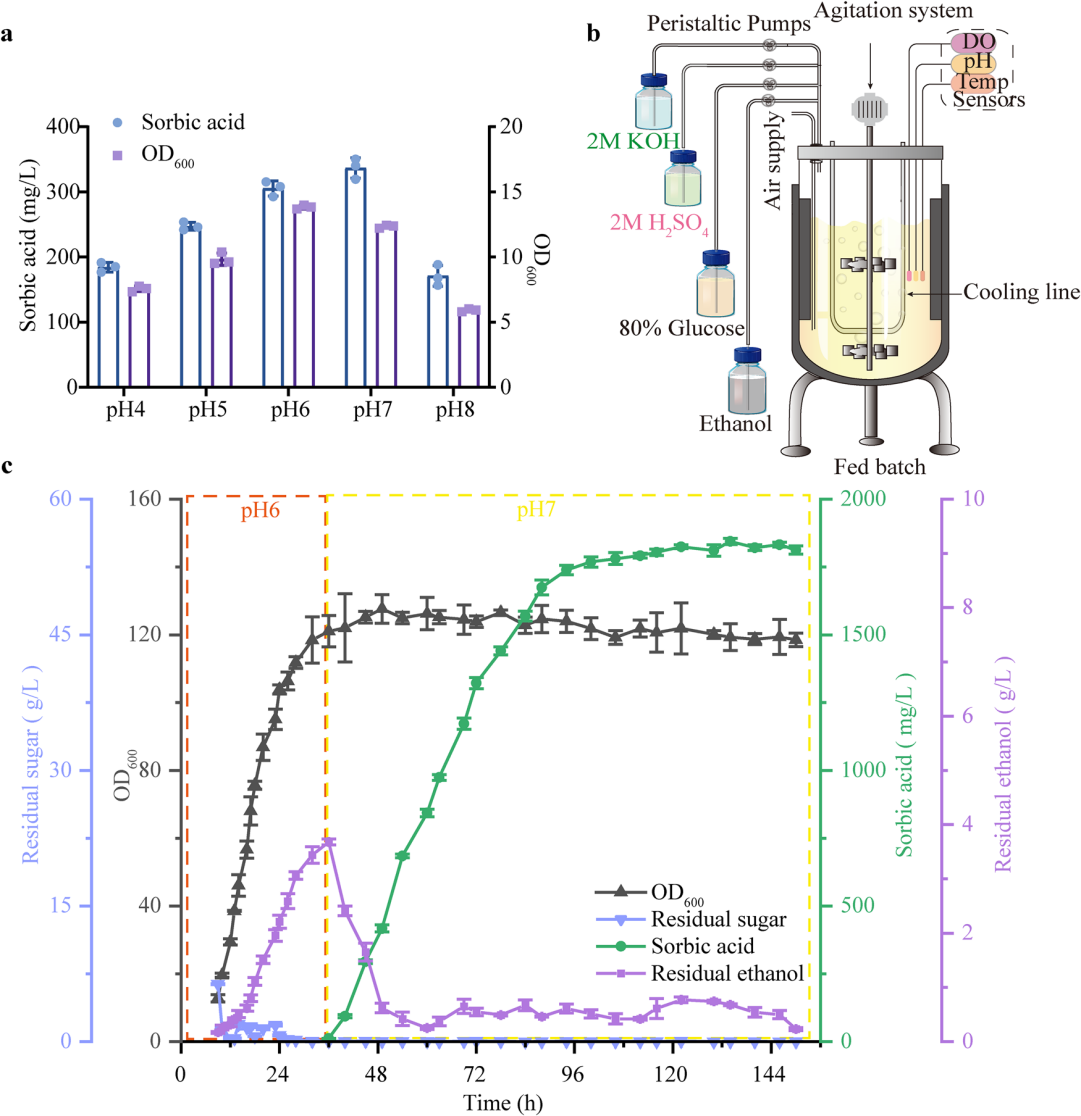
Scale-up cultivation and process optimization for SA production. **a** SA titers (blue bars) and cell density (OD₆₀₀, purple bars) of SJB51 in shake-flask cultures at different pH conditions (pH 4.0–8.0). Cultures were grown in 250 mL shake flasks with 50 mL YPD medium; pH was adjusted per 12 h using 2 M KOH or 2% H₂SO₄. **b** Schematic of the fed-batch bioreactor setup, including peristaltic pumps for substrate/agent feeding, an agitation system, and sensors for dissolved oxygen (DO), pH, and temperature monitoring. **c** Two-stage fed-batch fermentation process. In the first stage, glucose feeding at pH 6.0 supported biomass accumulation; in the second stage, ethanol feeding at pH 7.0 induced SA production. SA titer (green line), cell density (OD₆₀₀, black line), residual glucose (blue line), and residual ethanol (purple line) during fed-batch cultivation, with distinct phases at pH 6 (orange dashed box) and pH 7 (yellow dashed box) to illustrate process dynamics. Data are presented as mean ± s.d. (*n* = 3 biologically independent samples). Source data are provided as a Source data file. Panel (**b**) was prepared using Adobe Illustrator 2021.

The antimicrobial activity of SA is strongly pH-dependent, since its undissociated form readily permeates microbial membranes and disrupts intracellular metabolism, whereas the dissociated anion is less effective^52,53^. We therefore evaluated yeast growth and SA production under different pH conditions (pH was adjusted per 12 h using 2 M KOH or 2% H₂SO₄). In shake-flask fermentations, SJB51 exhibited optimal growth at pH 6.0 (OD₆₀₀ = 13.8), with SA titers reaching 305.3 mg/L. SA production increased with higher pH values and peaked at pH 7.0 (340 mg/L), although biomass decreased slightly (OD₆₀₀ = 12.3). At pH 8.0, both growth and SA accumulation declined (Fig. 5a), consistent with impaired cytoplasmic pH homeostasis under alkaline stress. Moreover, the IC₅₀ of SA against *S. cerevisiae* increased with pH (Supplementary Fig. 18a), underscoring the necessity of precise pH control in industrial processes.

We next investigated pH effects in fed-batch fermentation. At pH 6.0, cell growth was maximal (OD₆₀₀ = 128.6 at 60 h), and SA titers reached 1251.3 mg/L at 112 h (Supplementary Fig. 25a). At pH 7.0, biomass accumulation was lower (OD₆₀₀ = 105.7 at 65 h), yet SA synthesis was more efficient, with titers peaking at 1369.6 mg/L at 112 h (Supplementary Fig. 25b). Collectively, these results reveal a clear trade-off: acidic conditions promote robust cell growth, whereas mildly neutral conditions optimize SA biosynthesis.

Leveraging this principle, we designed a two-stage fed-batch process (Fig. 5b, c). In stage I (0–36 h), glucose feeding at pH 6.0 supported rapid biomass accumulation. In stage II (post-36 h), pH was shifted to 7.0 and ethanol was continuously supplied (0.3–0.5 g/L), inducing *pADH2*-driven expression and fueling acetyl-CoA supply. This dynamic regime achieved the highest performance: biomass reached OD₆₀₀ = 127.7 at 49 h, SA titers rose to 417.4 mg/L, and ultimately peaked at 1844.3 mg/L at 134 h (OD₆₀₀ = 119.3, Fig. 5c).

Together, these results demonstrate that synchronizing glucose- and ethanol-fed phases with dynamic pH control effectively decouples cell growth from product formation. By repressing *pADH2* during biomass accumulation and activating it during ethanol induction, this strategy resolves the conflict between growth and production, establishing a robust platform for industrial-scale SA biosynthesis in yeast.

## Discussion

The global transition from fossil fuel-dependent to bio-based production has driven intense interest in microbial cell factories for high-value chemicals^54,55^, However, three core challenges persist for many industrially critical molecules: decoding their biosynthetic pathways, engineering robust production chassis, and resolving product toxicity. SA, one of the world’s most widely used safe food preservatives, epitomizes this gap: despite its natural occurrence in some plants, commercial SA production still relies on fossil fuel-derived feedstocks (ketene and crotonaldehyde)^11,55^.

In this study, we isolated both SA and SN from filamentous fungus *Myrothecium* sp. FJNU6, elucidated its fungal biosynthetic pathway, and engineered *S. cerevisiae* to produce SA. Our study bridges this gap by establishing the first full bioproduction of SA via microbial fermentation, leveraging pathway discovery, metabolic engineering, and process optimization in *S. cerevisiae* (Supplementary Fig. 26).

By harnessing the metabolic capabilities of appropriately engineered microbial hosts, we envision a sustainable, efficient, and eco-friendly route to SA. While *E. coli* is famed for its rapid growth, genetic tractability, and high expression levels^3,56,57^, concerns over endotoxin contamination and biosafety significantly restrict its utility in food and nutraceutical applications. In contrast, *S. cerevisiae* (GRAS status) offers a safer, more versatile platform—combining industry-grade biosafety with advanced genome-editing tools such as CRISPR–Cas9, rapid growth, and scalable fermentation processes^58,59^. This makes yeast an increasingly preferred chassis for the industrial production of bioactive compounds^60,61^.

Engineering *S. cerevisiae* for SA production required coordinated optimization of gene expression, precursor supply, and pathway compartmentalization. Promoter engineering revealed that dynamic control is essential to balance growth and production^62^, as demonstrated in prior *S. cerevisiae* systems using inducible promoters to moderate metabolic burden and optimize toxic metabolite pathways^63^. Fusion of SA synthases via peptide linkers consistently reduced titers, likely reflecting the intricate multidomain and multimeric architecture of PKSs^64^ that resist simple proximity engineering. Redirecting carbon flux toward cytosolic acetyl-CoA was highly effective—aligning with metabolic engineering strategies that re-purpose native pathways to relieve key bottlenecks^65^. Overexpression of *PYC1*, *ME1/MDH3*, *YlACL1/2*, and *AsACC1* substantially increased titers, with combinatorial integration yielding a 6-fold improvement. This confirms acetyl-CoA as a central bottleneck for both fatty acid and polyketide biosynthesis^66^. Enhancing ethanol utilization offered another robust strategy to increase acetyl-CoA. Overexpression of *ADH2*, *ALD6*, and *ACS1/2* increased titers to 173.2 mg/L, underscoring the metabolic advantage of ethanol: it channels carbon directly into acetyl-CoA while simultaneously activating *pADH2*-driven pathway induction, creating a self-reinforcing production regime. However, manipulation of NADPH supply through the pentose phosphate pathway or TCA cycle enzymes did not improve SA titers. Unlike fatty acid synthesis, which requires 14 NADPH molecules per C16 fatty acid^33,40,41^, SA biosynthesis consumes only two NADPH molecules per unit. Endogenous supply therefore appears sufficient, highlighting how distinct cofactor requirements differentiate PKS pathways from fatty acid metabolism.

Among all strategies, peroxisomal engineering had the strongest impact. Targeting *CAT2* and deregulated *ACC1*^*S1157A*^ to peroxisomes increased acetyl-CoA and malonyl-CoA pools, while co-localization of *SoaA* and *SoaB* at the peroxisomal surface enhanced catalytic efficiency through proximity effects. Titers exceeded 253 mg/L—representing a 15-fold increase—establishing peroxisomes as powerful metabolic platforms, consistent with recent advances in compartmentalized pathway engineering^42–45,50^. Importantly, this strategy is likely applicable to a broad range of PKS- and NRPS-derived molecules.

The enhanced SA production observed upon peroxisomal targeting is consistent with the metabolic role of peroxisomes and their emerging utility for compartmentalizing heterologous pathways^43^. Peroxisomes are enriched in acetyl- and acyl-CoA intermediates derived from fatty acid metabolism and β-oxidation, which may increase precursor availability for polyketide assembly. Compartmentalization may also reduce competition with cytosolic malonyl-CoA-consuming pathways and limit acid-associated cytosolic stress. Together, these factors likely contribute to the improved pathway efficiency, although further quantitative analyses will be required to define their relative contributions.

Product toxicity remains a major challenge in yeast-based production, manifesting as slowed growth, altered metabolism, and reduced productivity^42,67,68^. SA toxicity is strongly pH-dependent, with optimal growth at pH 6.0 but maximal production at pH 7.0. To resolve this trade-off, we implemented a two-stage fed-batch process: glucose feeding at pH 6.0 for biomass accumulation, followed by ethanol feeding at pH 7.0 for production induction. This strategy yielded >1.8 g/L SA—over 7-fold higher than flask cultures and, to our knowledge, the highest reported titer in yeast. This demonstrates the power of synchronizing promoter regulation, carbon utilization, and pH control. Future improvements could include rational protein engineering or the introduction of resistance-conferring genes against SA’s cellular targets^69^.

Parallel advances in synthetic chemistry and biology have sought renewable routes to SA precursors, such as one-pot condensation of biomass-derived intermediates^11^ and microbial production of TAL, which can be chemically converted to SA with >75% yield^15,55,70^. Although these approaches reduce dependence on fossil fuel resource, they still require petrochemical inputs for downstream conversion. By contrast, direct biosynthesis of SA via elucidated gene clusters offers a fully biological and potentially carbon-neutral route, though the complete BGC remains to be fully characterized. Its discovery would unlock opportunities for both high-titer production and structural diversification through metabolic engineering.

From a commercial perspective, SA and KS are currently produced predominantly via petrochemical routes. Recent techno-economic analysis by Kim et al. of TAL-derived SK production estimated a minimum product selling price of ~$8.3/kg, exceeding prevailing market levels (approximately $5–6.5/kg)^18^.

For the biosynthetic pathway developed here, the theoretical maximum yield from glucose to SA via an acetyl-CoA-dependent route in yeast is approximately 0.41 g/g, providing a realistic carbon-efficiency benchmark. The current titer achieved in this study (1.84 g/L in a 50 L fermenter) represents an early-stage demonstration and remains below the levels typically required for large-scale commercialization of commodity organic acids.

Further advancement toward industrial implementation will require coordinated improvements in carbon flux toward acetyl- and malonyl-CoA, enhancement of acid tolerance, increased volumetric productivity, and process intensification strategies such as in situ product removal (e.g., two-phase extractive fermentation) to mitigate product-associated toxicity and reduce downstream processing burdens. These considerations position the present study as an enabling step toward sustainable SA biomanufacturing.

Together, our findings define a roadmap for microbial SA production in yeast: (i) inducible promoters to mitigate toxicity, (ii) precursor expansion through acetyl-CoA engineering, (iii) avoidance of generic linker fusions in PKS systems, (iv) peroxisomal compartmentalization to enhance flux, and (v) dynamic cultivation strategies for industrial translation. Beyond SA, this work underscores the versatility of *S. cerevisiae* as a sustainable production platform for diverse polyketide-derived natural products.

In conclusion, our work establishes a fully sustainable, high-titer route to SA via engineered *S. cerevisia*e, decoding its biosynthetic pathway and overcoming key challenges of toxicity, precursor limitation, and process scalability. While further optimization will be required to achieve commercial competitiveness, this study provides a mechanistic and engineering blueprint for translating polyketide-derived molecules into sustainable biomanufacturing platforms, contributing to the broader transition toward a bio-based economy.

## Methods

### Strains and culture conditions

Different microbial strains were employed to meet the requirements of cloning, protein expression, genetic engineering, and metabolite production. *Escherichia coli* strains were cultivated in LB medium (1% NaCl, 0.5% yeast extract, 1% tryptone). *E. coli* DH10B was used for routine cloning, while *E. coli* BL21 (DE3) was employed for recombinant protein expression^71^. Antibiotic concentrations were adjusted according to standard protocols (50 μg/mL kanamycin or 100 μg/mL ampicillin, unless otherwise indicated).

*Myrothecium* sp. FJNU6 was cultured on potato dextrose agar (PDA) at 25 °C. For rice fermentation, spores of FJNU6 (5% v/v inoculum from PDB) were transferred into sterile rice medium (125 g rice, 200 mL water, 2 g MgSO₄, 1.3 g KH₂PO₄) and incubated 30 d at 25 °C. Biomass and medium (~0.5 cm³) were extracted in 800 µL EtOAc/MeOH/AcOH (89:10:1), vortexed (1 min), sonicated (10 min), and centrifuged (12,000 × *g*, 5 min). Supernatants were dried, dissolved in 100 µL MeOH, clarified by centrifugation, and analyzed by LC–MS.

*Aspergillus nidulans* LO8030 was cultivated in either CD medium (1% glucose, 5% [v/v] 20× N-salts, 0.1% [v/v] trace elements, and 2% agar) at 37 °C for protoplast transformation, or CDST medium (2% starch, 2% peptone, 5% [v/v] 20× N-salts, 0.1% [v/v] trace elements, and 2% agar) at 25 °C for heterologous expression and metabolite production^72^.

*Saccharomyces cerevisiae* BJ5464-NpgA was used to construct sorbic acid-producing strains^73^. For genetic manipulation, yeast was grown in YPD medium (2% glucose, 1% yeast extract, 2% peptone) supplemented with selective antibiotics (100 μg/mL nourseothricin sulfate (NAT) and 250 μg/mL G418). For plasmid selection, dropout synthetic media lacking specific nutrients (Ura, Trp, Leu) were used.

### Bioinformatics analyses

Protein and gene sequences were analyzed using BLAST (NCBI; https://blast.ncbi.nlm.nih.gov). Biosynthetic gene clusters were predicted using antiSMASH 8.0 (fungal version). SnapGene (GSL Biotech LLC) was used for annotation, restriction site identification, and cloning design. Multiple sequence alignment was carried out with ClustalW, and phylogenetic trees were reconstructed using the maximum-likelihood method in MEGA X^74^.

### Gene amplification and plasmid construction

DNA manipulations followed manufacturers’ recommendations. Restriction enzymes were purchased from New England Biolabs (NEB). Genomic DNA was extracted using phenol–chloroform, and high-fidelity PCR was performed with SparkJade polymerase. Seamless assembly was carried out using Gibson Assembly Master Mix (NEB).

Total RNA was extracted using an RNA extraction kit (Takara) and reverse-transcribed with HiScript III First Strand cDNA Synthesis Kit (Vazyme). Primers were synthesized, and all plasmids were sequence-verified (Sangya Biotech).

Genomic DNA of *Myrothecium* sp. FJNU6 was used to amplify *soaA* (with primer pairs soaA-1F/R and soaA-2F/R), *soaB* (soaB-1F/R), and *soaC* (soaC-1F/R). Gibson assembly was used to construct recombinant plasmids in *E. coli*. Intron-free *soaA/B/C* genes were amplified from cDNA and cloned for expression in *S. cerevisiae*. Endogenous elements (promoters, ORFs, and terminators) were amplified from the *S. cerevisiae* genome. gRNA target sites were designed using CHOPCHOP. Details of primers and engineered *S. cerevisiae* strains used in this study are summarized in Supplementary data 1 and 2. All primer sequences were designed using SnapGene 6.0.2 software and synthesized by Fuzhou Shangya Biotechnology Co., Ltd.

### CRISPR–Cas9 genome editing in yeast

Yeast competent cells expressing Cas9 were co-transformed with donor DNA (containing homology arms) and gRNA plasmids. Transformations were incubated at 30 °C for 1.5 h with mixing every 20 min. Cells were centrifuged (500 × *g*, 5 min), resuspended in 2 mL YPD, and incubated at 30 °C, 220 rpm for 2 h. Selection was performed in YPD medium supplemented with G418 (200 μg/mL) and NAT (100 μg/mL) for 6 h, followed by plating on YPD agar with the same antibiotic concentrations. Plates were incubated for 3–4 d at 30 °C, and colonies were verified by PCR^75^.

### Activity detection of the crude fermentation extract of *Myrothecium* sp. FJNU 6

The antimicrobial activities were tested against *C. albicans* CICC 1965. The strains for bioassay were purchased from China Center of Industrial Culture Collection (CICC). *C. albicans* was cultured in yeast extract peptone dextrose (YPD) medium at 30 °C for 24 h. Cell (Spore) density was measured by direct counts using a hemocytometer and adjusted to 1 × 10^6^ cells (spore)/mL. And positive control (hygromycin B) was dissolved in MeOH to prepare a stock solution (50 mg/mL). Oxford Cup Diffusion Method: The microbial suspension (200 µL) was spread evenly onto YPD agar medium. Three Oxford cups were placed on each plate, and 10 µL of test solution, positive control, or MeOH (negative control) was added per cup. Plates were incubated in 30 °C for 24 h. Inhibition zones were measured and compared to controls.

### Determination of NADPH content

Single colonies of recombinant *S. cerevisiae* were picked and inoculated into 50 mL YPD medium for overnight cultivation (12 h). For recombinant strains harboring 2μ-based expression plasmids, cells were cultured in SD-Ura-Trp dropout medium for 24 h. The pre-cultures were inoculated into 250-mL shake flasks containing 50 mL YPD medium at an inoculation ratio of 10% (v/v), followed by fermentation at 30 °C with shaking at 220 rpm. After cultivation, cells were harvested by centrifugation at 17,000 × *g* and 4 °C for 10 min, and the supernatant was discarded. Cell pellets were washed twice with ddH₂O. For NADPH measurement, cells from 1 mL culture were resuspended in 500 μL NADPH extraction buffer, snap-frozen in liquid nitrogen, and then ground thoroughly. The lysate was centrifuged at 12,000 × *g* and 4 °C for 10 min, and the supernatant was collected. The protein concentration of each sample was determined, and all samples were adjusted to a uniform protein concentration using NADPH extraction buffer. To decompose NADP⁺, the adjusted samples were incubated in a water bath at 60 °C for 30 min. Then, 200 μL of the diluted supernatant was transferred to a 96-well plate as the test sample, followed by the addition of 10 μL chromogenic reagent. After incubation at 37 °C in the dark for 10 min, the absorbance at 450 nm was measured.

### Heterologous expression

Plasmids were introduced into *A. nidulans* LO8030 using PEG-mediated protoplast transformation^76^. Transformants were plated on CD medium and incubated at 37 °C for 3 d, then single colonies were propagated and transferred onto CDST medium at 25 °C for 4 d for LC–MS analysis.

Plasmids were introduced into *S. cerevisiae* BJ5464-NpgA by the lithium acetate method^77^. Colonies were grown for 3 d in dropout medium, expanded in shake flasks for 2 d, and inoculated into YPD medium for 3 d prior to LC–MS analysis.

### Protein expression and purification

Recombinant plasmids were transformed into *E. coli* BL21 (DE3). Overnight cultures grown in LB medium with kanamycin (50 μg/mL) were inoculated (5%) into 1 L LB medium containing kanamycin (100 μg/mL). Cells were grown at 37 °C until OD₆₀₀ reached 0.6. After cooling on ice, protein expression was induced with IPTG (final concentration 0.1 mM) and incubated overnight at 16 °C.

Cells were harvested by centrifugation, resuspended in 50 mL buffer A (25 mM Tris-HCl, 200 mM NaCl, 10% [v/v] glycerol, pH 7.4), and lysed by sonication. Lysates were centrifuged at 12,000 × *g* for 30 min at 4 °C and filtered through a 0.45 μm membrane. Supernatants were loaded onto Ni–NTA resin pre-equilibrated with binding buffer. Elution was performed stepwise with imidazole (25, 50, 100, 200, and 300 mM) in buffer A. Fractions containing the target protein were pooled, concentrated by ultrafiltration, and stored in buffer B (25 mM Tris-HCl, 10% glycerol, pH 7.4) at −80 °C^69^.

The recombinant *S. cerevisiae* BJ5464-NpgA strain for protein expression was cultured in deficient medium for 24 h. The pre-cultured seed culture was inoculated at a 10% inoculum size into 3 L shake flasks containing 1 L of YPD medium and fermented at 30 °C with shaking at 220 rpm. The bacterial cells were harvested by centrifugation at 8000 × *g* and 4 °C for 10 min, washed twice with washing buffer (25 mM Tris-HCl, 200 mM NaCl), and then collected and stored at −80 °C for subsequent use.

For the purification of SoaA and SoaB, 100 g of frozen bacterial cells were resuspended in 100 mL of Buffer A (25 mM Tris-HCl, 200 mM NaCl, 10% [v/v] glycerol, pH 7.4), and the cells were disrupted by ultrasonic treatment at 4 °C. The lysate was centrifuged at 12,000 × *g* and 4 °C for 30 min to remove cell debris, and the resulting supernatant was filtered through a 0.45-μm filtration membrane. Subsequently, 3 mL of Ni-NTA resin equilibrated with Buffer A was added to the filtered supernatant, and the centrifuge tube was gently rotated on a shaker for 2 h to achieve sufficient binding of the target proteins to the resin. The mixture was transferred to a 20 mL gravity flow chromatography column, which was first washed with 60 mL of Buffer A. Stepwise elution was then performed using Buffer A supplemented with imidazole at gradient concentrations (25, 50, 100, 200 and 300 mM), and the eluate was collected in 5 mL fractions per tube. Fractions containing the target proteins were pooled, concentrated by ultrafiltration, and stored at −80 °C in Buffer B (25 mM Tris-HCl, 10% [v/v] glycerol, pH 7.4).

### In vitro activity assay

All sample addition steps for the in vitro enzymatic activity assay were performed on ice. The standard reaction system (final volume: 100 μL) consisted of 25 mM Tris-HCl buffer (pH 7.4), 5 mM NADPH, 1 mM acetyl-CoA, 2 mM malonyl-CoA, 30 μM SoaA and 20 μM SoaB. The reaction was incubated at 25 °C overnight and then quenched by the addition of 100 μL of acetonitrile. After vacuum drying, the precipitate was dissolved in 100 μL of methanol and centrifuged at 12,000 × *g* for 5 min; the supernatant was finally analyzed by liquid chromatography-mass spectrometry (LC–MS). Heat-denatured enzymes (treated at 95 °C for 10 min) were used as the negative control.

For the in vitro enzymatic activity assay coupled with base hydrolysis, the reaction system (final volume: 300 μL) contained 25 mM Tris-HCl buffer (pH 7.4), 2 mM NADPH, 1 mM acetyl-CoA, 2 mM malonyl-CoA and 100 μM SoaA. After overnight incubation at 25 °C, 30 μL of 1 M NaOH was added to the system, which was then heated at 65 °C for 10 min. The reaction mixture was subjected to vacuum drying, and the resulting precipitate was dissolved in 100 μL of methanol and centrifuged at 12,000 × *g* for 5 min. The supernatant was subsequently analyzed by LC–MS.

Amidase activity was measured in 100 μL reactions containing 10 mM substrate, 10 mM ATP, 10 mM MgCl₂, 10 mM glutamine, and purified enzyme in 25 mM Tris-HCl (pH 7.4). Reactions were incubated at 37 °C for 20 min, quenched with 100 μL acetonitrile, dried under vacuum, dissolved in 100 μL methanol, centrifuged at 12,000 × *g* for 5 min, and analyzed by LC–MS. Heat-denatured enzyme (95 °C, 10 min) was used as negative control.

The structures of SoaA and SoaB were predicted with AlphaFold 3 using default parameters^78^. Models were visualized with PyMOL (Open Source 2.6.2, Schrödinger LLC). Molecular docking was performed with AutoDock Vina^79^.

### Metabolite extraction and LC–MS/HPLC analysis

Samples were extracted three times with 1 mL solvent mixture (89% ethyl acetate, 10% methanol, 1% acetic acid), centrifuged at 17,000 × *g* for 5 min, and the organic phase was collected. Extracts were dried under reduced pressure, re-dissolved in 100 μL HPLC-grade methanol, and subjected to LC–MS or HPLC analysis. For heterologous expression experiments, fermentation extracts from *A. nidulans* or *S. cerevisiae* strains carrying the corresponding empty vector were used as negative controls.

For LC–MS analysis, a Thermo Ultimate 3000 HPLC system coupled to an LCQ Fleet mass spectrometer (Thermo Scientific) was used with a Phenomenex Luna C18(2) column (150 × 2.1 mm, 5 μm) maintained at 30 °C. Solvent A consisted of water with 0.1% formic acid and solvent B consisted of acetonitrile with 0.1% formic acid. The gradient program was 5–95% B over 30 min at a flow rate of 0.25 mL/min. Detection wavelength ranged from 190–800 nm. Ion source conditions were set as follows: spray pressure 35 psi, drying gas flow 35 mL/min, source temperature 350 °C, and scan range *m*/*z* 50–160 in both positive and negative ion modes. Raw LC–MS data were processed using Thermo Xcalibur software (version 2.2.0.48). For HPLC analysis, a Thermo Ultimate 3000 HPLC system equipped with an Agilent TC-C18 column (4.6 × 250 mm, 5 μm) was used. The gradient was 5–95% solvent B (0.1% formic acid in acetonitrile) over 20 min, followed by a 5 min hold at 95% B and re-equilibration at 5% B for 5 min. The flow rate was 1 mL/min, the column temperature was maintained at 25 °C, and detection was performed at 260 nm. The injection volume was 10 μL. HPLC data were processed using Thermo Chromeleon software (version 7.2.10.24543). Each biological sample was injected once for LC–MS or HPLC analysis.

### High-cell-density fermentation

Single colonies were inoculated into 5 mL SD-Ura-Trp medium and cultured at 30 °C for 24 h. Seed cultures were transferred into 1 L SD-Ura-Trp medium in 3 L flasks and incubated for 20 h, then inoculated into a 50 L fermenter containing 45 L medium (20 g/L glucose, 10 g/L (NH_4_)_2_SO_4_, 8 g/L KH₂PO₄, 3 g/L MgSO₄, 0.72 g/L ZnSO₄·7H₂O, 10 mL/L trace element solution, 12 mL/L vitamin solution), 10% inoculation.

Fermentation was conducted at 30 °C with pH maintained at 6.0 or 7.0 by automated addition of 2 M KOH. Dissolved oxygen was maintained above 20% saturation by adjusting agitation speed (300–800 rpm) and aeration rate (1–3 vvm). A concentrated feed (800 g/L glucose and 12 mL/L vitamins) was supplied during exponential growth. In the final stage, ethanol was continuously added to promote sorbic acid accumulation until fermentation was terminated^80^.

### Statistics & reproducibility

All experiments were performed with at least three biologically independent replicates unless otherwise stated to ensure reproducibility. Data are presented as mean ± s.d. where applicable. No statistical method was used to predetermine sample size. No data were excluded from the analyses. The experiments were not randomized, and the investigators were not blinded to allocation during experiments and outcome assessment.

### Reporting summary

Further information on research design is available in the Nature Portfolio Reporting Summary linked to this article.

## Supplementary information

Supplementary Information

Peer Review File

Description of Additional Supplementary Files

Supplementary Data 1

Supplementary Data 2

Reporting Summary

## Source data

Source data

## Data Availability

The nucleotide sequence of the *soa* gene cluster generated in this study has been deposited in the GenBank database under accession code PX619210. The information of primers used for plasmid construction and the details of engineered strains constructed in this study are provided in Supplementary Data 1 and 2. For third-party data used in this work, the protein structures include PDB IDs 1CT9 and 7YLZ. Source data are provided with this paper.
