# Supplementary material for: Toward sustainable food preservatives: high-level production of sorbic acid in engineered Saccharomyces cerevisiae
Source: Nat Commun. 2026 Apr 21;17:5492. doi: 10.1038/s41467-026-72163-8 (PMC13284220; doi:10.1038/s41467-026-72163-8)
Supplement: Supplementary file 1 — Supplementary Information [file 41467_2026_72163_MOESM1_ESM.pdf]

Supplementary information

Toward sustainable food preservatives: high-titer production of sorbic acid in engineered *Saccharomyces cerevisiae*

Jianbin Xiao,[1, 2, #] Wei Lin,[1, 2, #] Xingtong Chen,[1, 2] Haoyu Yu,[1, 2] Chao Chen,[1, 2] Qin Li,[1,2] Fan Cai,[1, 2] Huaidong Zhang,[1, 2] Huibin Chen,[2] Mingliang Zhang,[1, 2, *] Yongjin J. Zhou,[3, *] Li Li,[1, 2, *]

1. Engineering Research Center of Industrial Microbiology (Ministry of Education), Fujian Normal University, Fuzhou 350117, P. R. China

2. College of Life Sciences, Fujian Normal University, Fuzhou 350117, P. R. China

3. Division of Biotechnology, Dalian Institute of Chemical Physics, Chinese Academy of Sciences, Dalian, China.

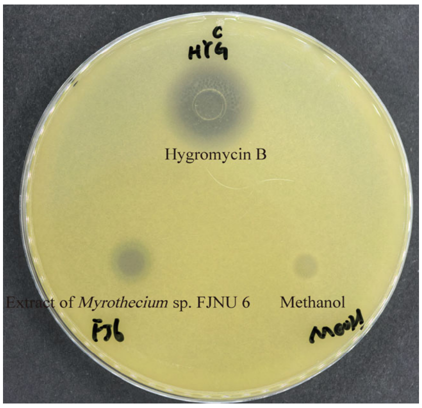

Supplementary Fig. 1 Anti-*Candida* activity of *Myrothecium* sp. FJNU6 extract. *Myrothecium* sp. FJNU6 was cultivated on rice medium at room temperature for 30 days. The culture was extracted with an appropriate extraction solution, and the extract was evaporated to dryness. The resulting residue was dissolved in methanol for testing the antimicrobial activity against *C. albicans* CICC 1965. A 200 µL suspension of *C. albicans* CICC 1965 was spread evenly onto YPD agar medium. Three Oxford cups were placed on each plate, and 10 µL of the test solution, positive control, or methanol (negative control) was added to each cup, respectively. Plates were incubated at 30 °C for 24 hours. Source data are provided as a Source Data file.

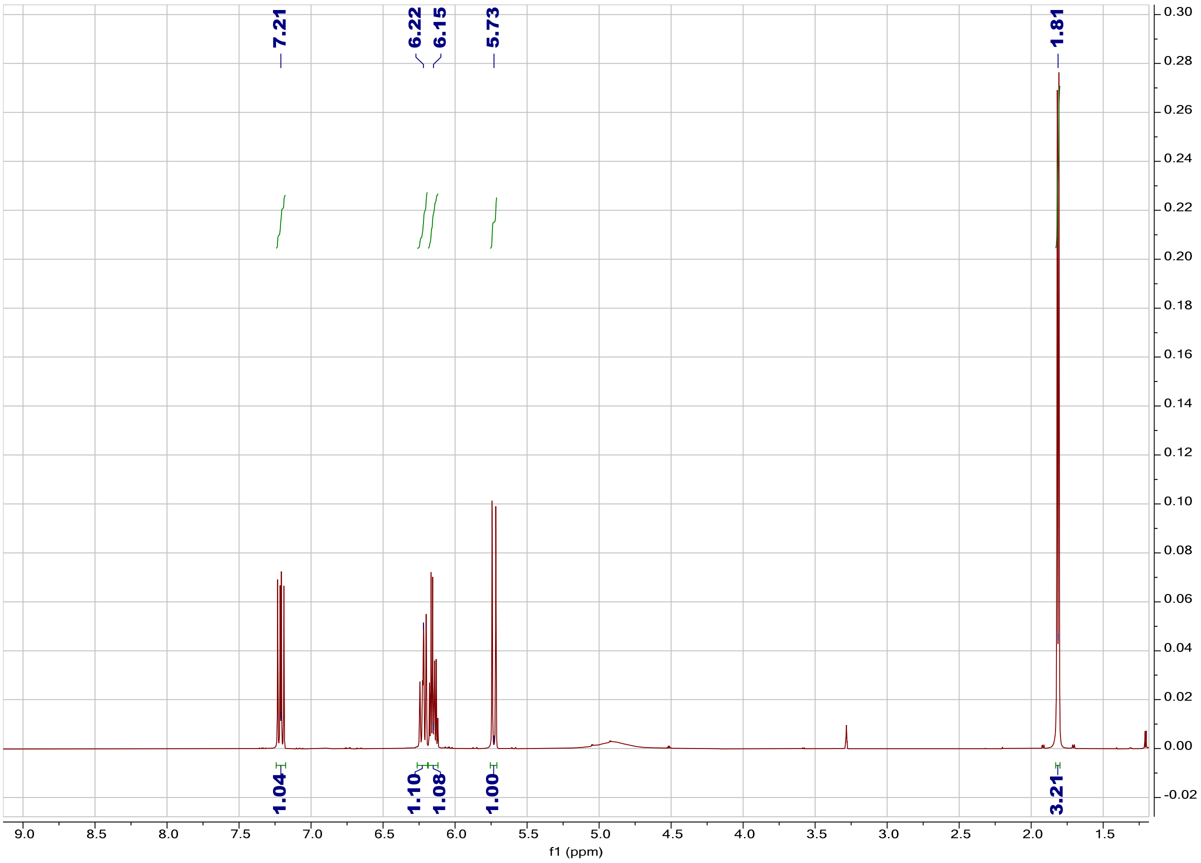

Supplementary Fig. 2. ^{1}H NMR spectrum of sorbic acid in CD_3OD.

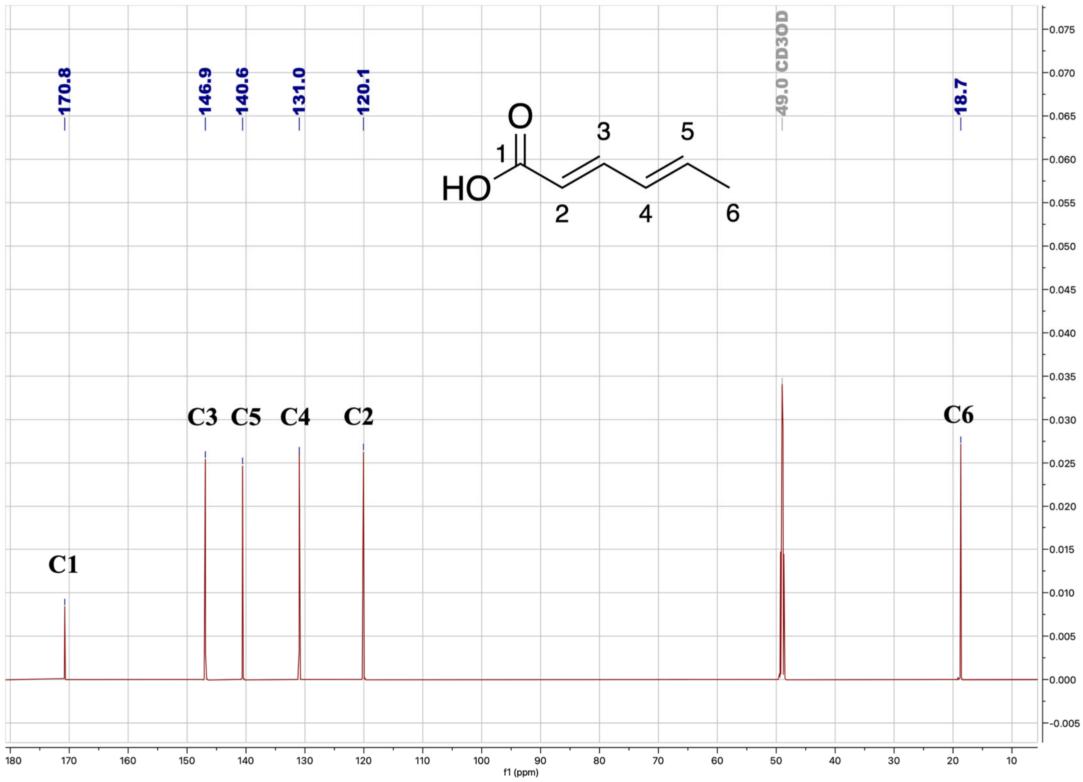

Supplementary Fig. 3. ^{13}C NMR spectrum of sorbic acid in CD_3OD.

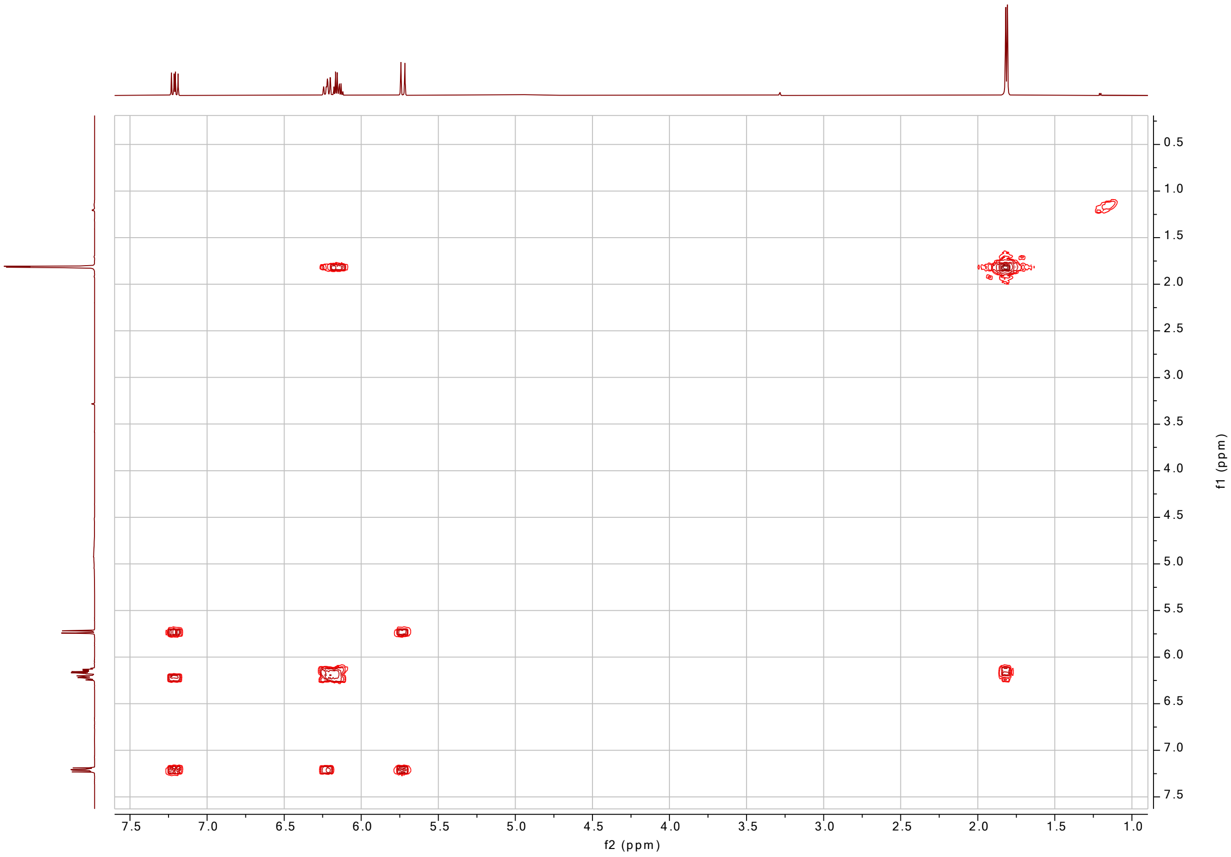

Supplementary Fig. 4. COSY spectrum of sorbic acid in CD_3OD.

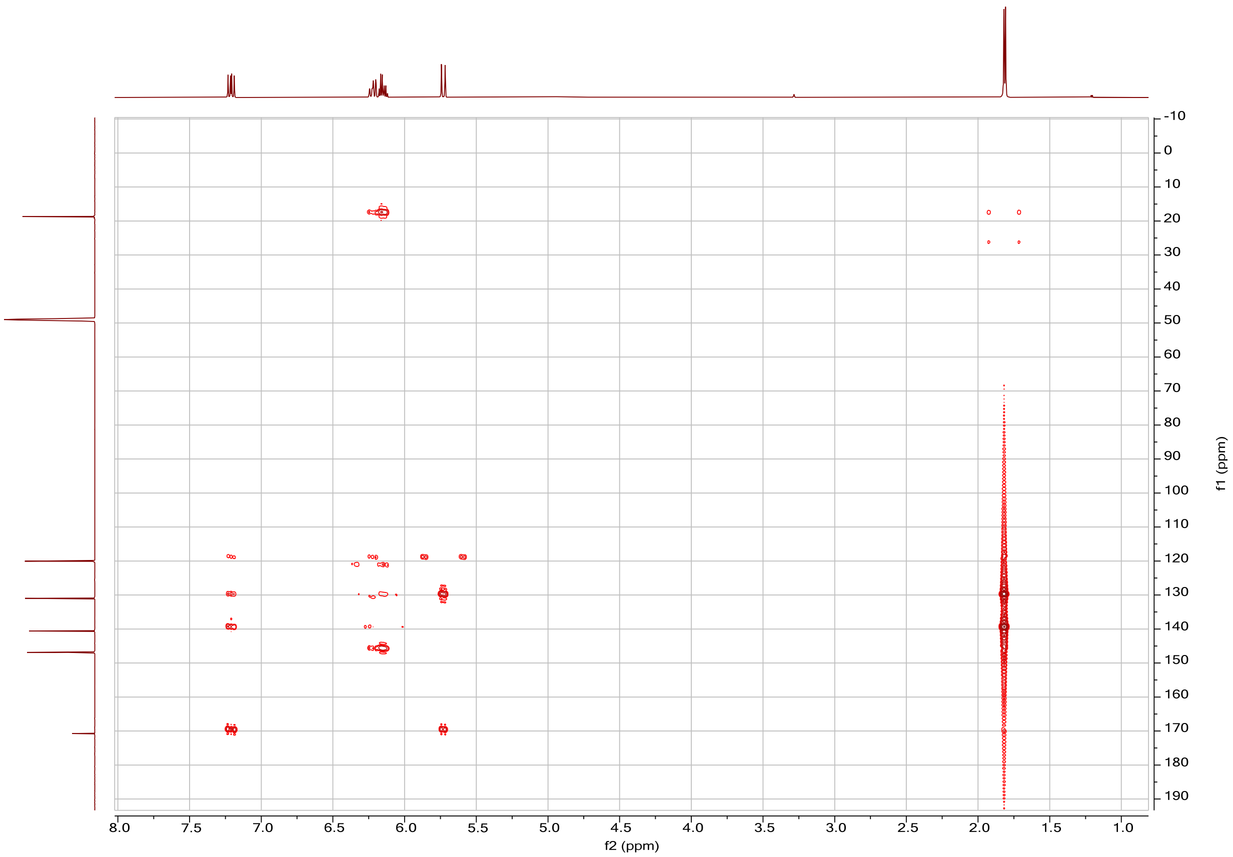

Supplementary Fig. 5. HMBC spectrum of sorbic acid in CD_3OD.

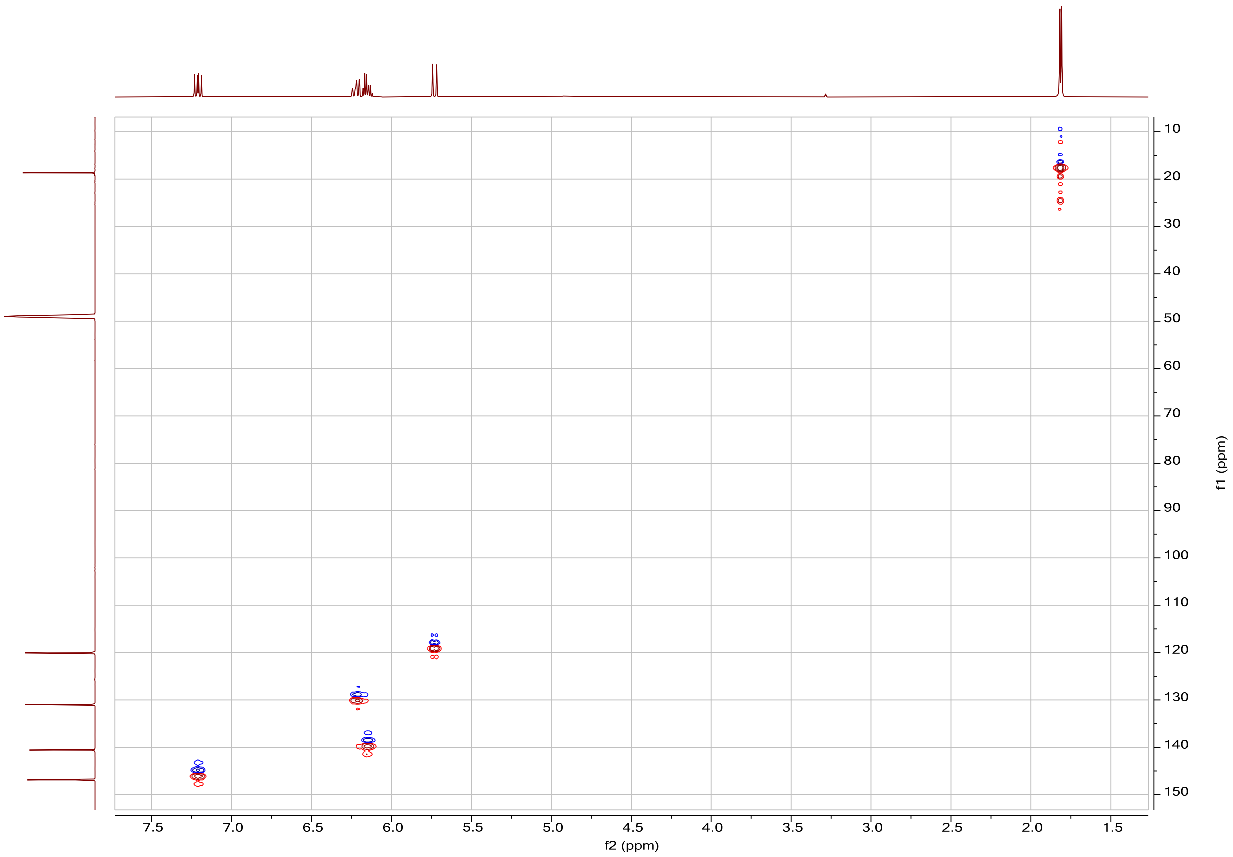

Supplementary Fig. 6. HSQC spectrum of sorbic acid in CD_3OD.

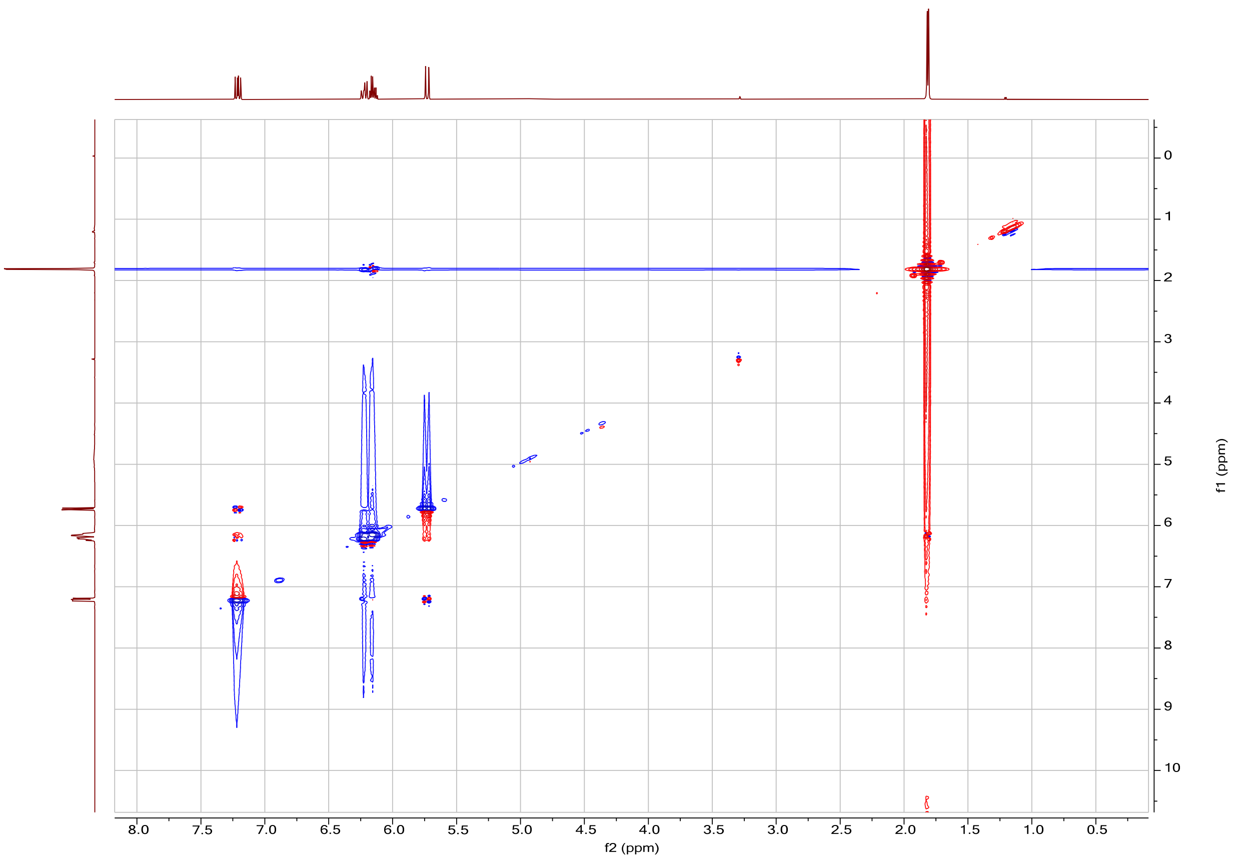

Supplementary Fig. 7. NOESY spectrum of sorbic acid in CD_3OD.

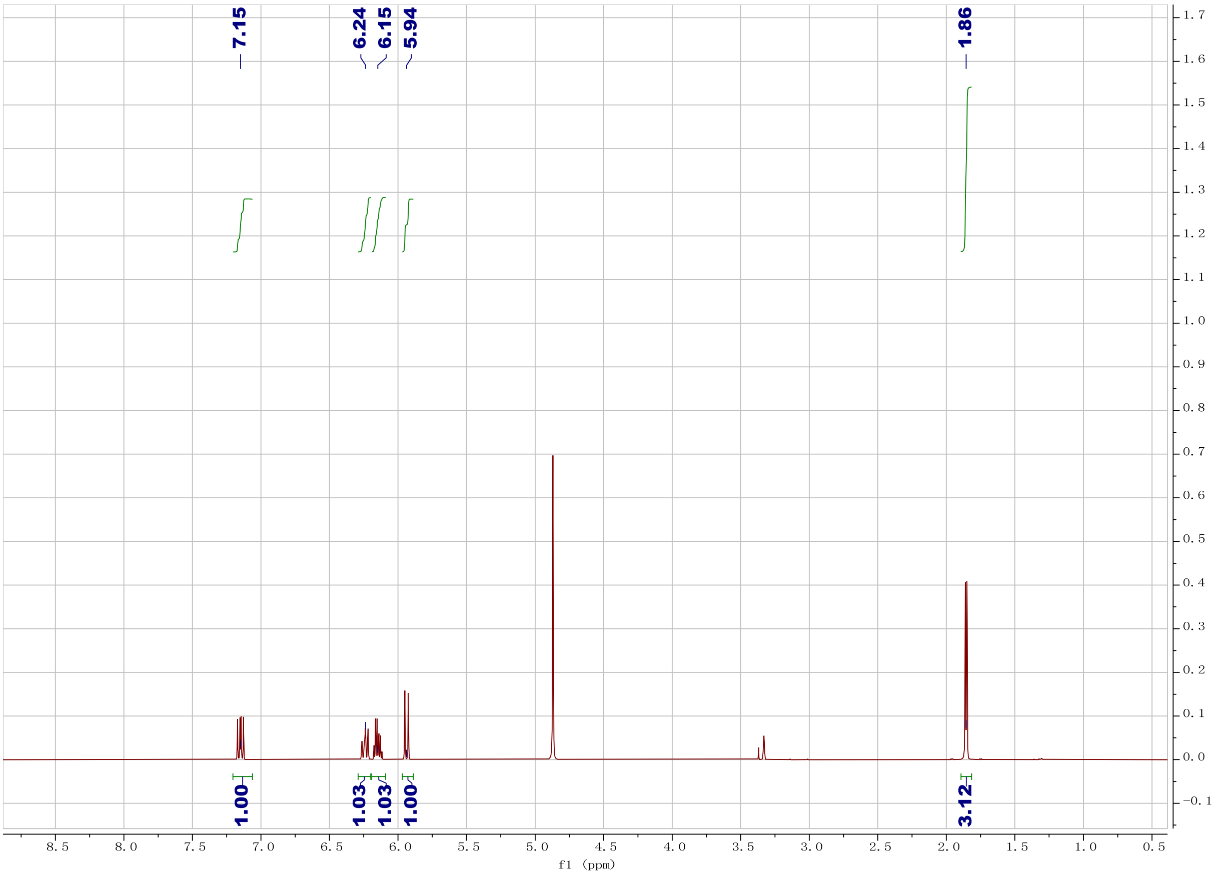

Supplementary Fig. 8. ^1H NMR spectrum of sorbamide in CD_3OD.

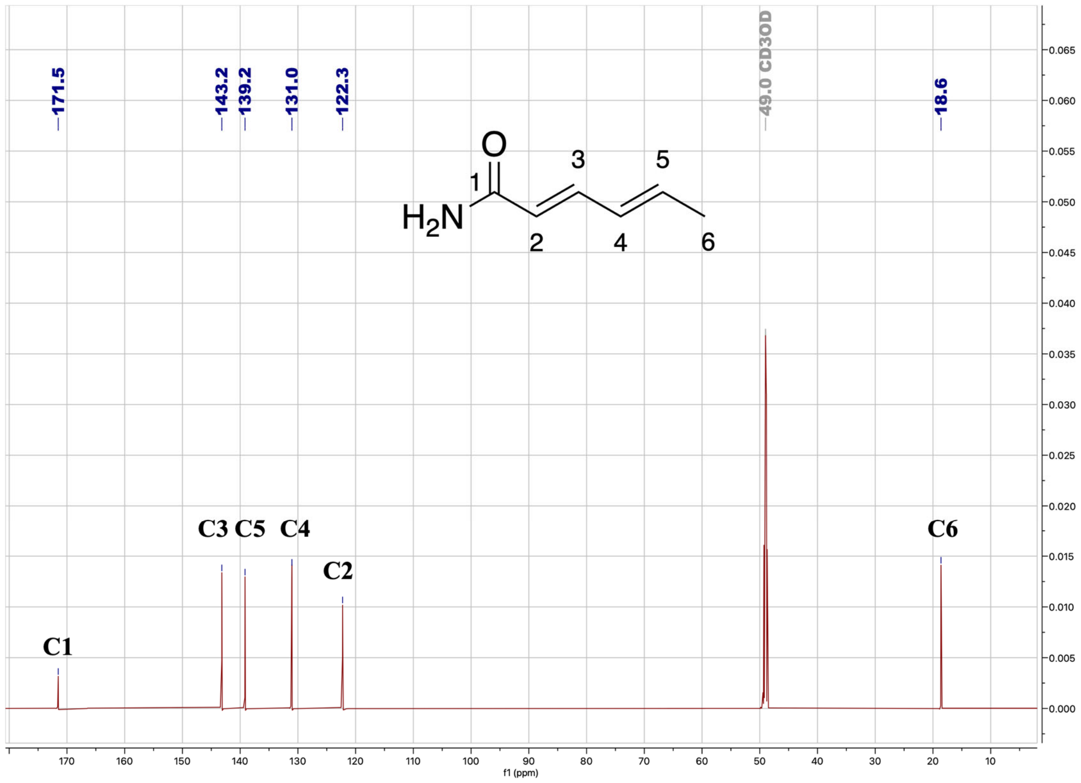

Supplementary Fig. 9. ^{13}C NMR spectrum of sorbamide in CD_3OD.

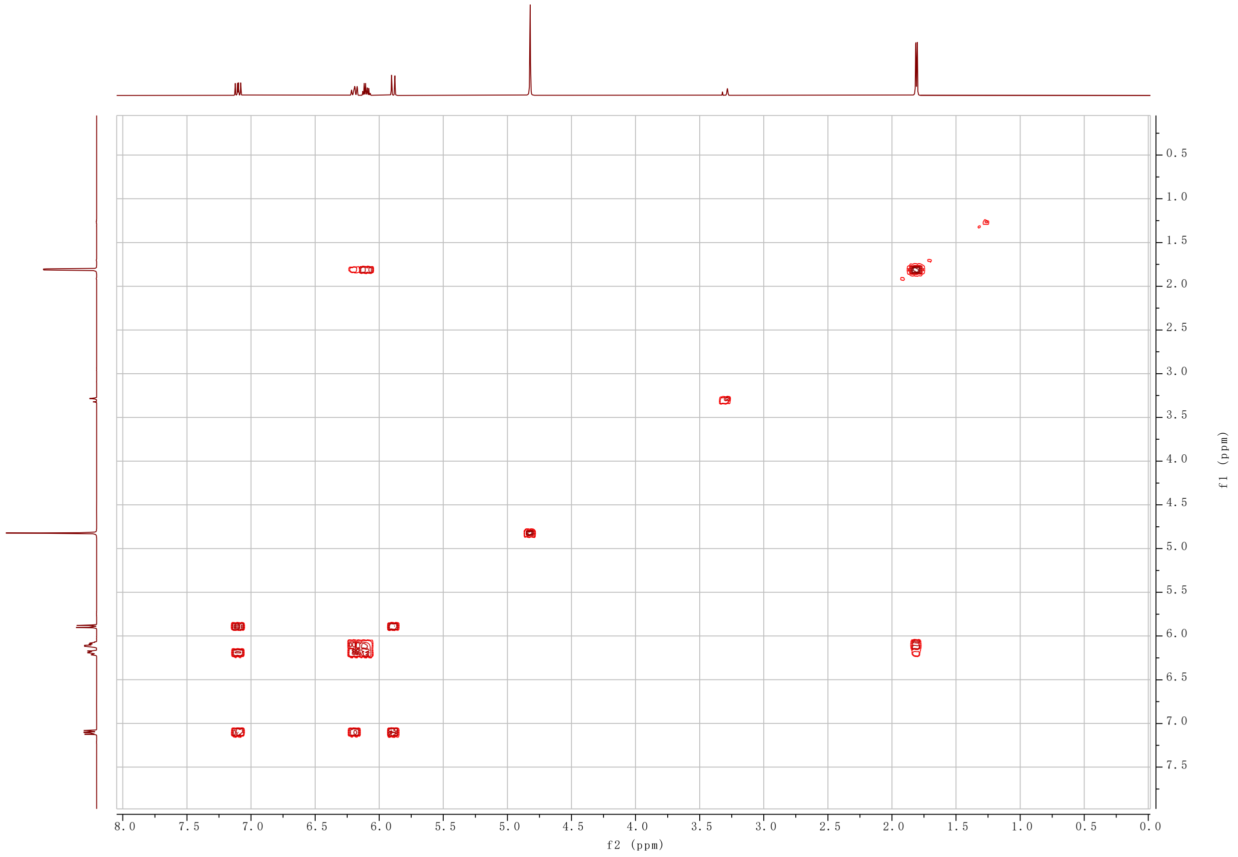

Supplementary Fig. 10. COSY spectrum of sorbamide in CD_3OD.

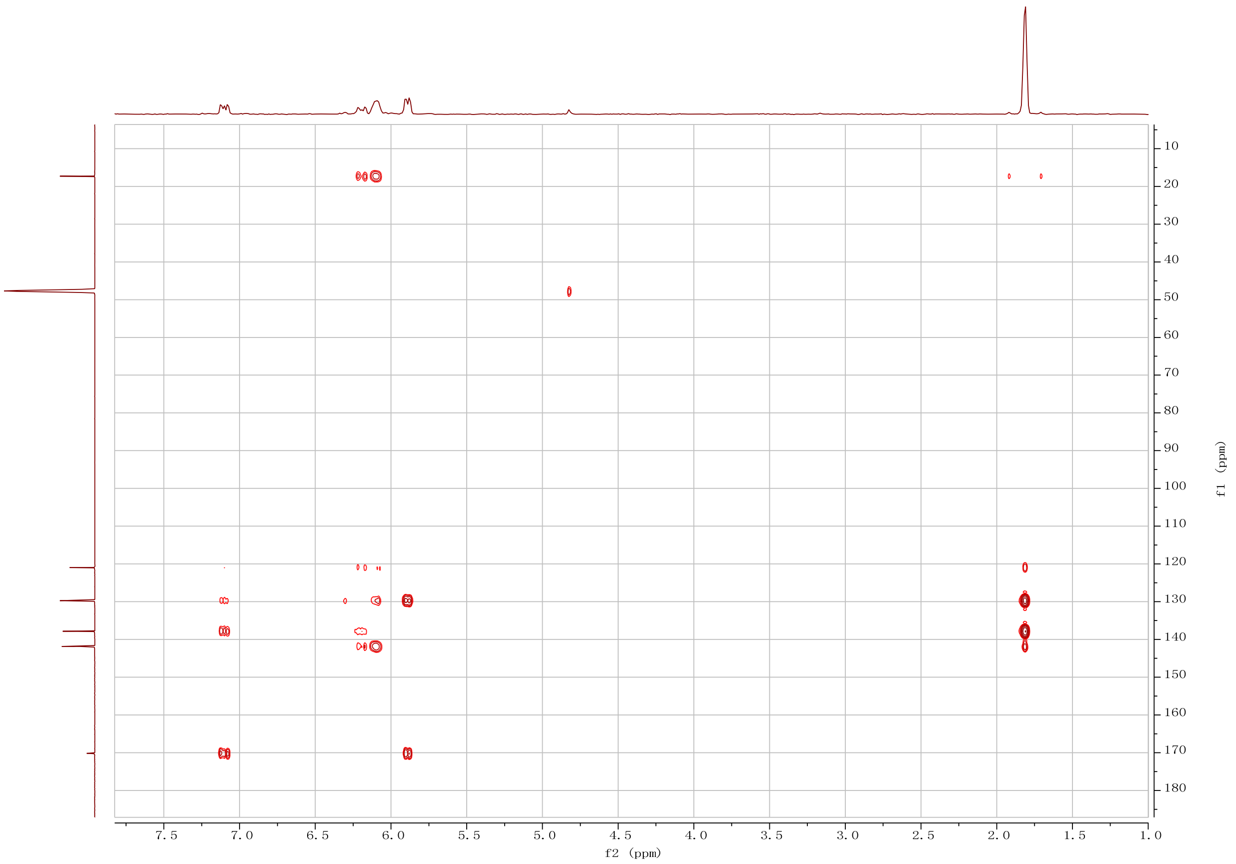

Supplementary Fig. 11. HMBC spectrum of sorbamide in CD_3OD.

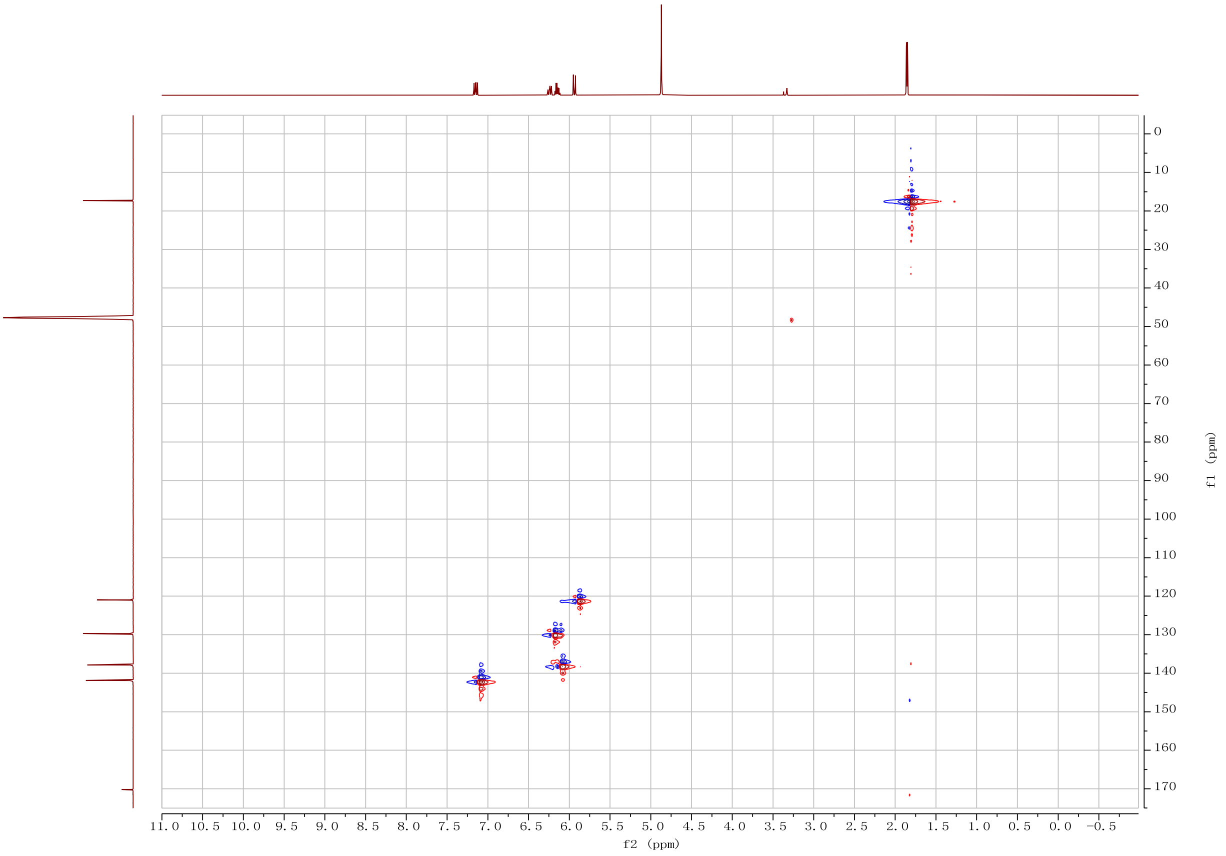

Supplementary Fig. 12. HSQC spectrum of sorbamide in CD_3OD.

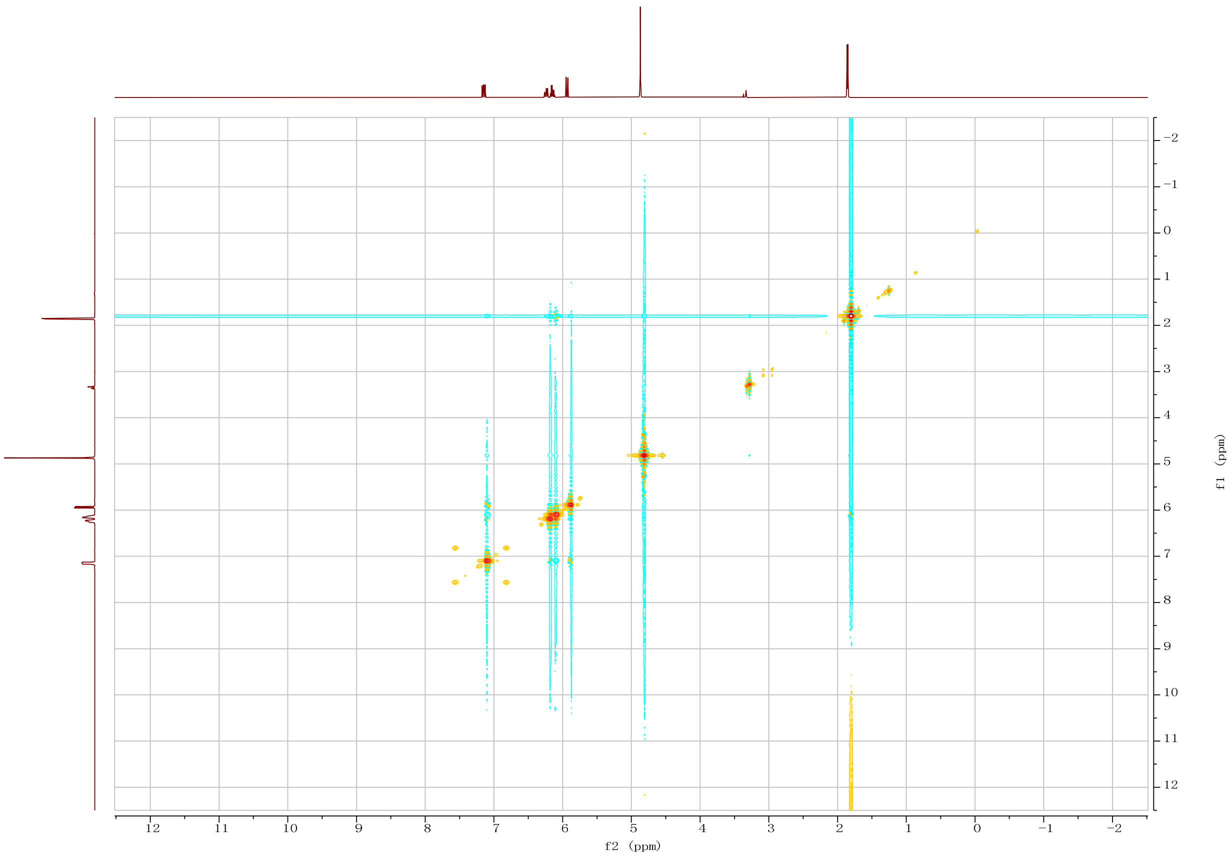

Supplementary Fig. 13. NOESY spectrum of sorbamide in CD_3OD.

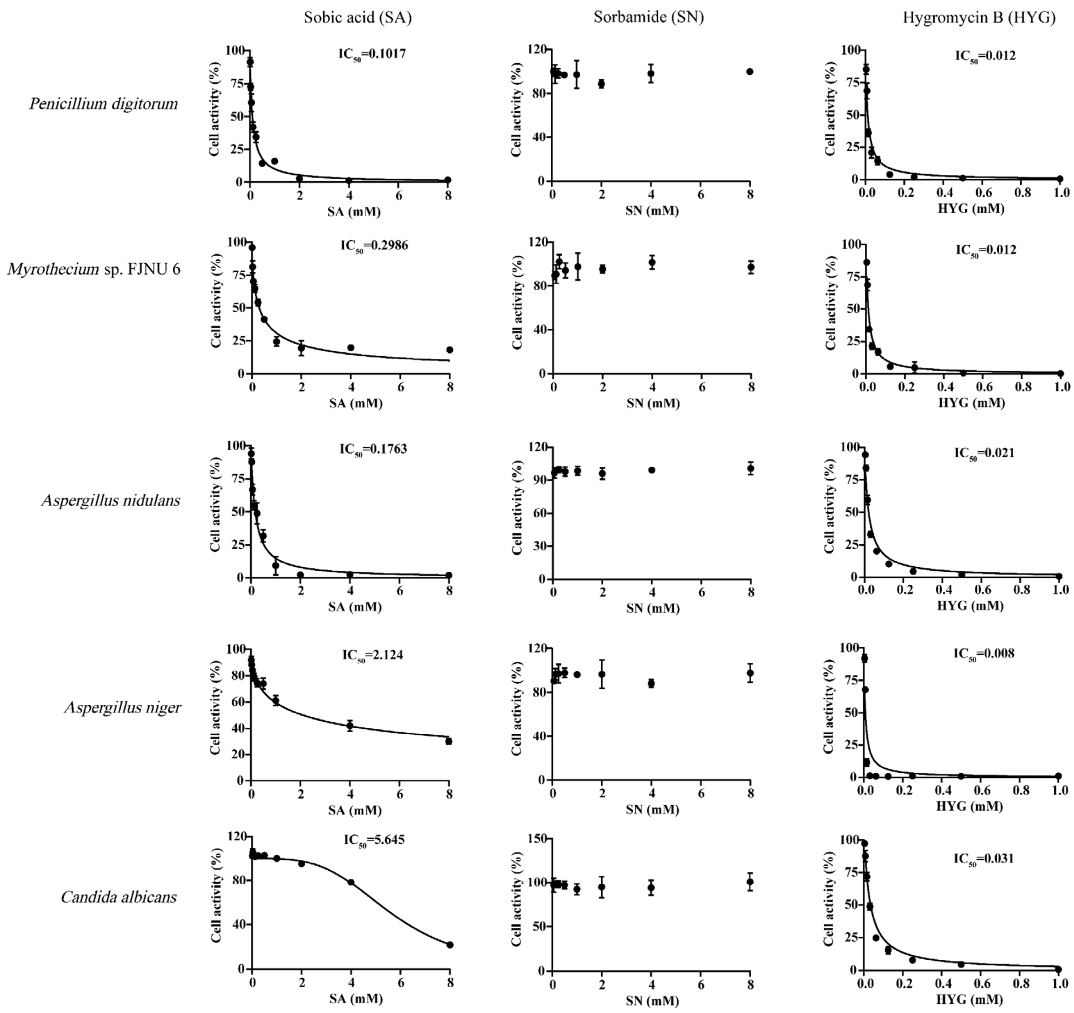

Supplementary Fig. 14. Antifungal activities of SA and SN against *Penicillium digitorum*, *Myrothecium* sp. FJNU 6, *Aspergillus nidulans*, *Aspergillus niger*, and *Candida albicans*. Cell activity was measured at different concentrations of each compound, and the half-maximal inhibitory concentration (IC_{50}) values are indicated for each fungal strain and compound. Error bars represent the standard deviation from three independent experiments. Data are presented as mean ± s.d. (n = 3 biologically independent samples). Source data are provided as a Source Data file.

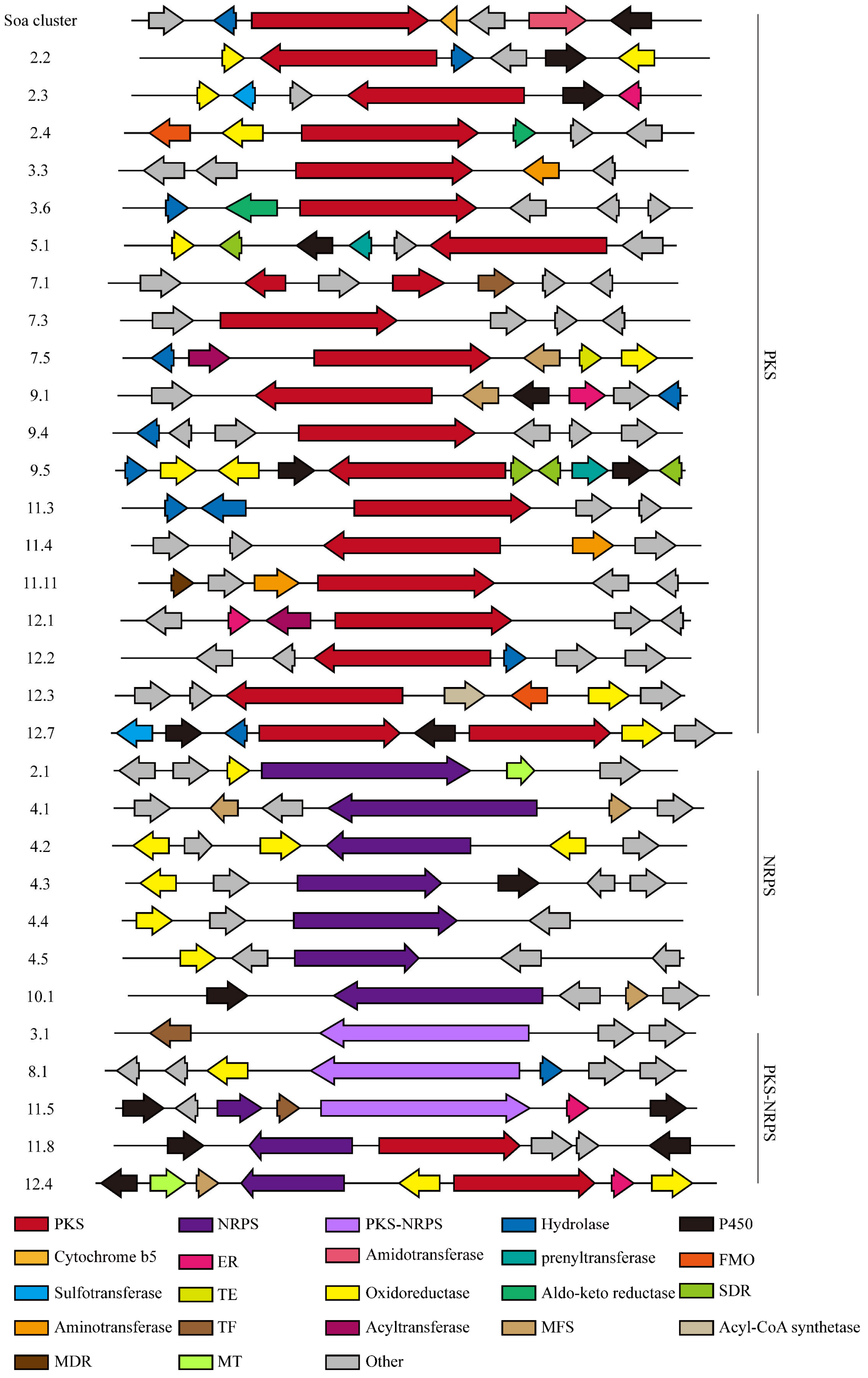

Supplementary Fig. 15. Schematic representation of the biosynthetic gene cluster from *Myrothecium* sp. FJNU6. The gene cluster is organized into three major modules, comprising a polyketide synthase (PKS), a nonribosomal peptide synthetase (NRPS), and a hybrid PKS–NRPS. The arrangement of these genes suggests a modular enzymatic architecture responsible for assembling structurally complex secondary metabolites.

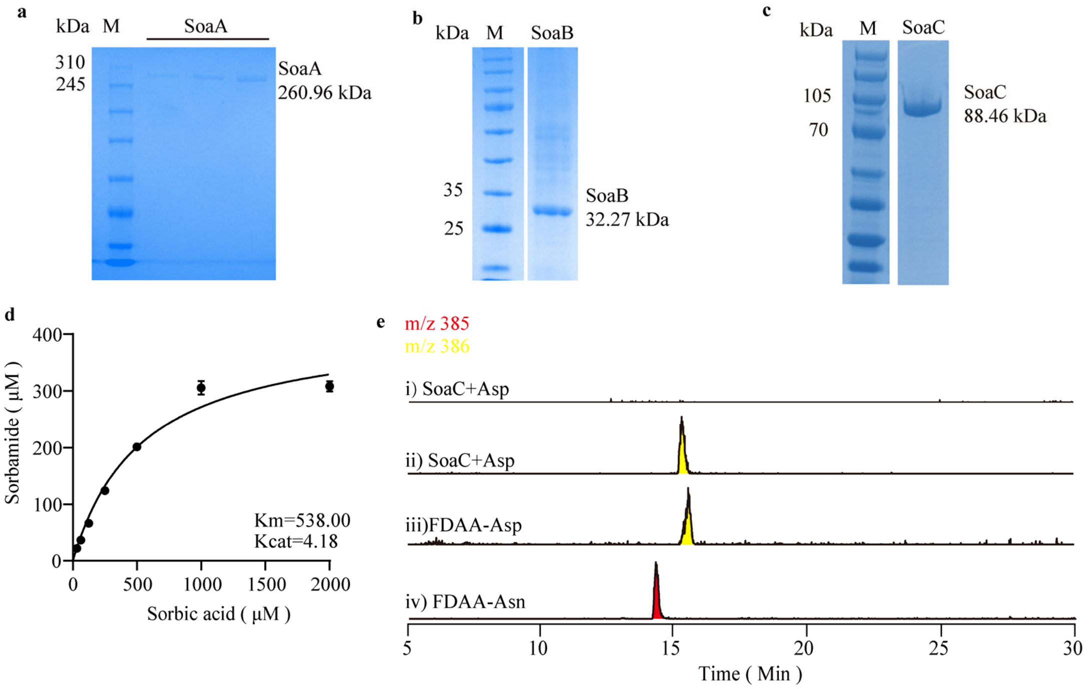

Supplementary Fig. 16 Characterization of SoaA/B/C and SoaC enzymatic kinetics. a, SDS-PAGE analysis of purified SoaA. Lane M, protein molecular weight marker (labeled in kDa); lane SoaA, purified SoaA with an apparent molecular mass of 260.96 kDa. **b**, SDS-PAGE analysis of purified SoaB. Lane SoaB, purified SoaB with an apparent molecular mass of 32.27 kDa. **c**, SDS-PAGE analysis of purified SoaC. Lane SoaC, purified SoaC with an apparent molecular mass of 88.46 kDa. **d**, Steady-state kinetic analysis of SoaC-catalyzed sorbamide production using sorbic acid as the substrate. The Michaelis-Menten plot shows the relationship between sorbic acid concentration (μM) and sorbamide production (μM), with derived kinetic parameters Km=538.00 μM and kcat=4.18 s^{-1}. Error bars represent the standard deviation from triplicate experiments. **e**, LC-MS analysis of reactions: (i) SoaC + Asp, (ii) SoaC + Asp (*m/z* 386, yellow), (iii) FDAA-Asp (*m/z* 386, yellow), and (iv) FDAA-Asn (*m/z* 385, red). FDAA: N-(2-furoyl) dansylalanine. Data are presented as mean ± s.d. (n = 3 biologically independent samples). Source data are provided as a Source Data file.

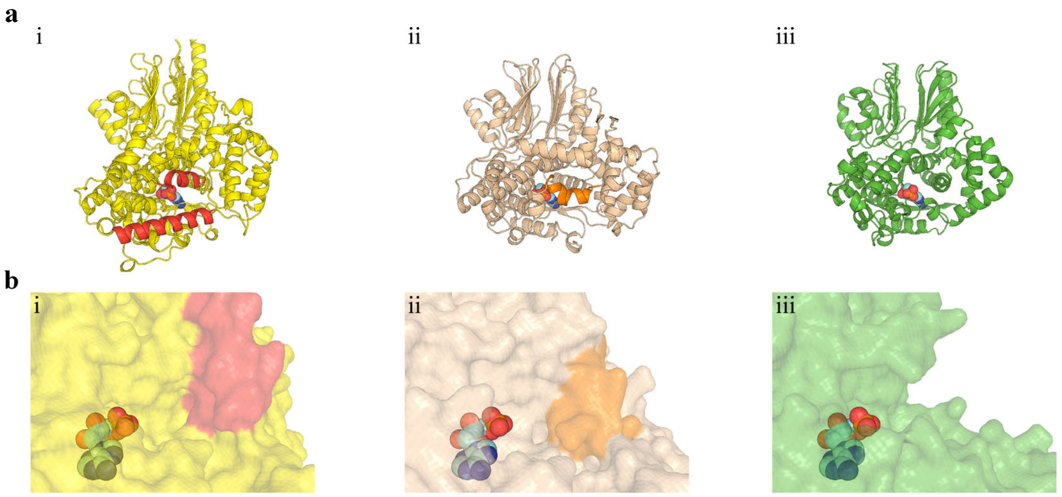

Supplementary Fig. 17. Structural Comparison between SoaC, TsnB9, and AsnB. (a) Overall structures of SoaC (i), TsnB9 (ii, PDB entry 7YLZ), and AsnB (iii, PDB entry 1CT9). The blue arrows highlight the differences in their active pockets, specifically the additional amino acid segments, with SoaC shown in red and TsnB9 in orange. In these overall structures, AMP is depicted as a spheres model. (b) Surface representation of the acceptor-binding site in SoaC (i), TsnB9 (ii), and AsnB (iii). The AMP in each enzyme, after superposition with that from the AsnB structure.

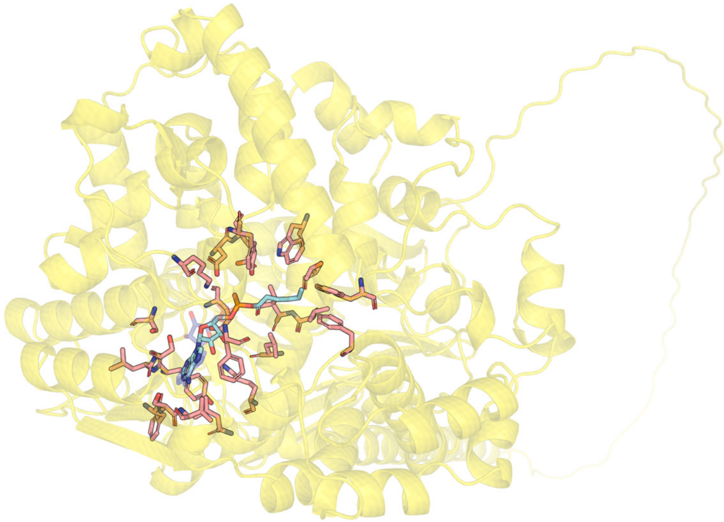

Supplementary Fig. 18. Molecular docking model of SoaC with SA-AMP. The amino acid residues within 4 Å of SA-AMP. SA-AMP and AMP are depicted as stick models, with AMP shown in a semitransparent representation.

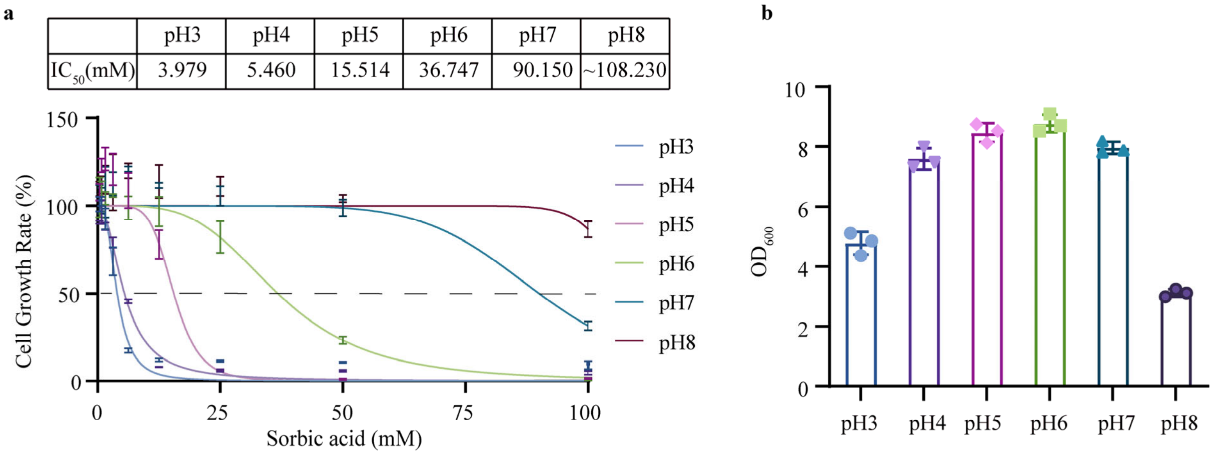

	pH3	pH4	pH5	pH6	pH7	pH8
IC_{50}(mM)	3.979	5.460	15.514	36.747	90.150	~108.230

Supplementary Fig. 19. Effect of SA concentration on cell growth rate of *S. cerevisiae* at different pH values. a, The graph depicts cell growth rate (%) as a function of sorbic acid concentration (mM) across pH 3 to 8. **b**, Effect of YPD medium at different pH values on the growth of *S. cerevisiae.* Error bars represent standard deviations. The accompanying table lists the half-maximal inhibitory concentration (IC_{50}, mM) of sorbic acid for cell growth at each pH. pH was adjusted per 12 h using 2 M KOH or 2% H_2SO_4. Data are presented as mean ± s.d. (n = 3 biologically independent samples). Source data are provided as a Source Data file.

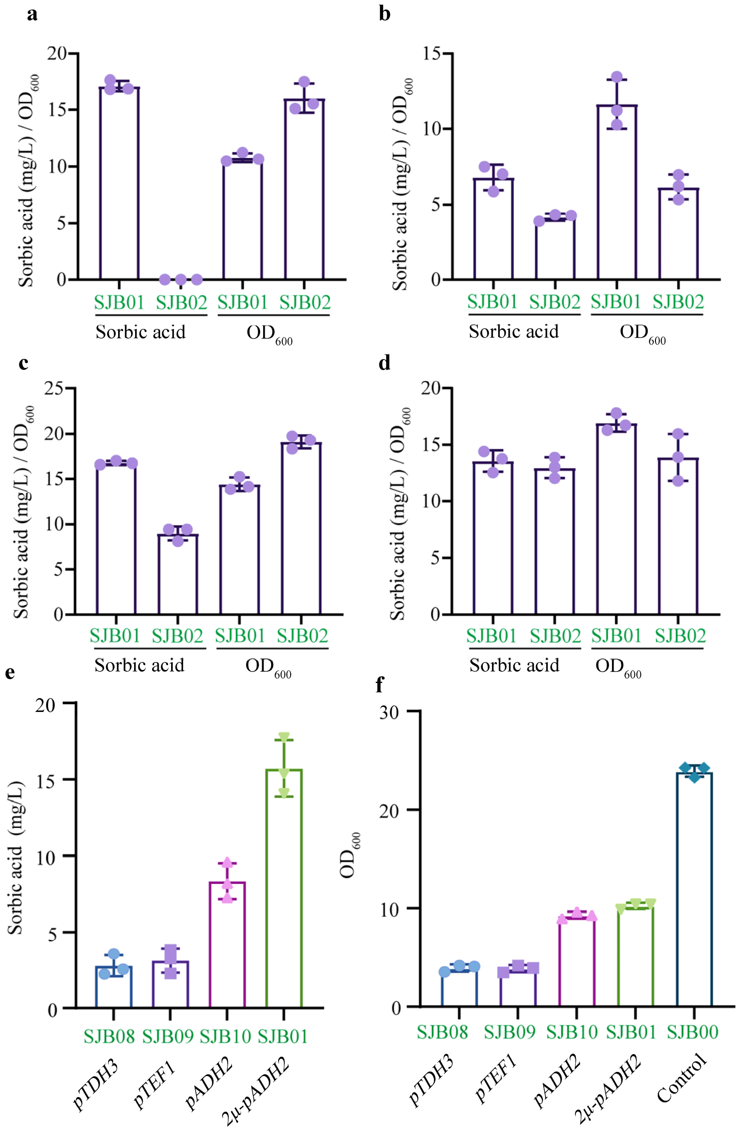

Supplementary Fig. 20. Influence of different promoters and integration strategies on SA production. Effects of different carbon sources on growth and SA production of the engineered strains. **a,** glucose; **b,** galactose; **c,** glucose+galactose; **d,** galactose supplementation after 24 h of glucose fermentation. Strains were fermented at 30℃ and 220 rpm for 48 h. Expression of SoaA and SoaB was regulated by three constitutive promoters *(pTDH3*, *pTEF1*, and *pADH2*) and integrated at distinct loci in *S. cerevisia*e (SoaA at X3, SoaB at 1021b). Strains SJB08, SJB09, and SJB10 were cultured in PYD medium for 3 days, after which SA titers (**e**) and cell density (monitored by OD_{600}, **f**) were measured. These results illustrate the impacts of promoter strength and chromosomal context on the output of the SA biosynthesis pathway. Data are presented as mean ± s.d. (n = 3 biologically independent samples). Source data are provided as a Source Data file.

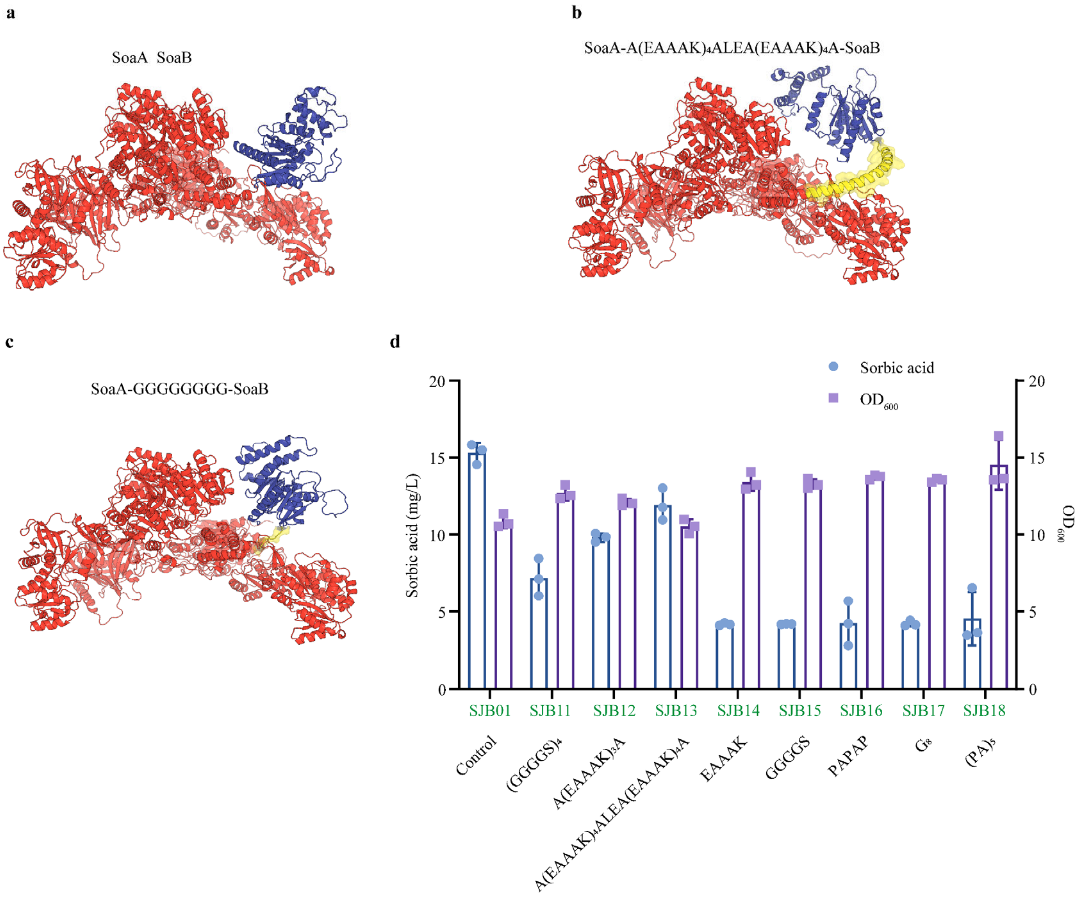

Supplementary Fig. 21. Structural models and functional characterization of SoaA–SoaB fusion proteins with different linkers and their impact on SA production. a, Structural model of the native SoaA (red) and SoaB (blue) complex. **b,** Structural model of the SoaA–(EAAAK)$_4$ALEA(EAAAK)$_4$–SoaB fusion protein, with the linker region highlighted in yellow. **c,** Structural model of the SoaA–GGGGGGGG–SoaB fusion protein, where the linker is shown in yellow. **d,** Bar graph depicting sorbic acid titers (blue bars, left y-axis) and cell density (OD$_{600}$, purple bars, right y-axis) for various engineered strains (SJB01 as control and SJB11–SJB18 with different linkers: (GGGG)$_4$, A(EAAAK)$_4$A, A(EAAAK)$_4$ALEA(EAAAK)$_4$A, EAAAK, GCGGS, PAPAP, G$_8$, (PA)$_4$). Error bars represent standard deviations from biological replicates. Data are presented as mean ± s.d. (n = 3 biologically independent samples). Source data are provided as a Source Data file.

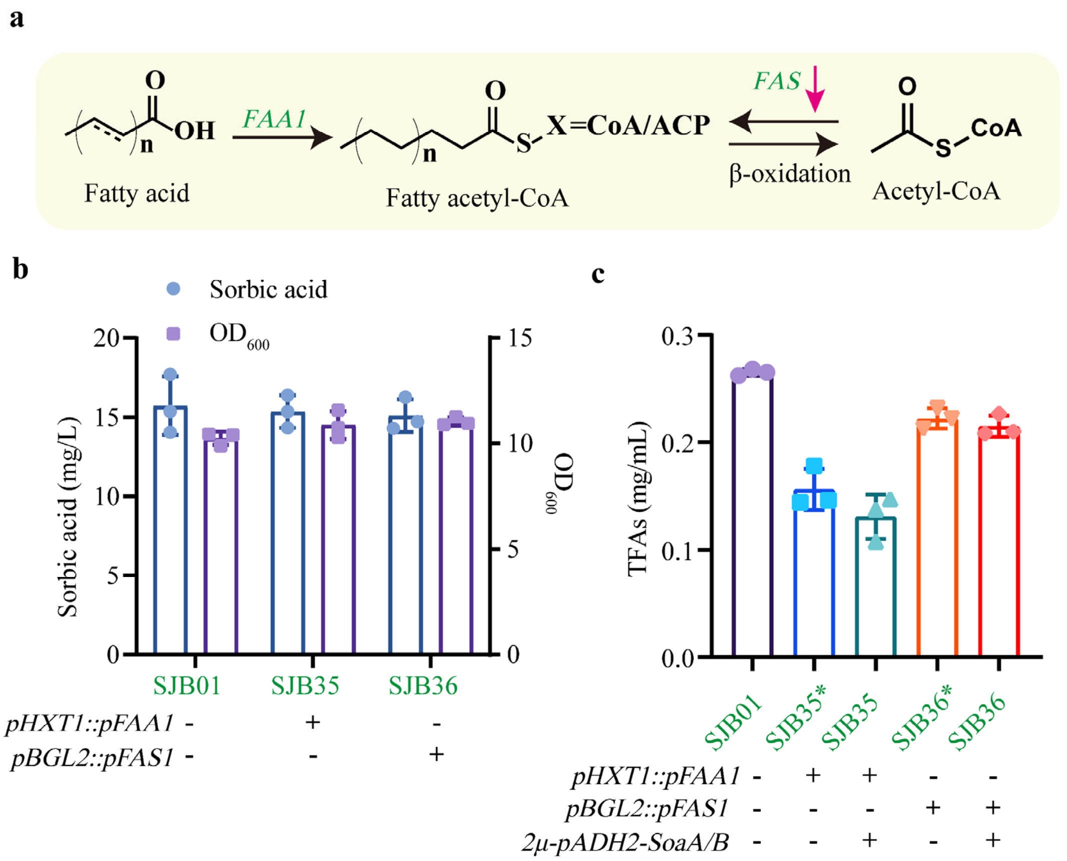

Supplementary Fig. 22. Engineering fatty acid metabolism for SA production. a, Schematic of fatty acid metabolic pathways involved in sorbic acid biosynthesis, including fatty acid activation by FAA1 to form fatty acetyl-CoA, and interconversion with acetyl-CoA via β-oxidation and fatty acid synthase (FAS)-mediated synthesis. **b,** Sorbic acid titers (blue bars) and cell density (OD_{600}, purple bars) in strains SJB01 (control), SJB35 (with *pHXT1::pFAA1*), and SJB36 (with *pBGL2::pFAS1*), cultivated to assess the impact of FAA1 and FAS1 manipulation. **c,** Total fatty acid (TFA) levels in strains SJB01, SJB35 (with and without *2μ-pADH2-SoaA/B*), and SJB36 (with and without *2μ-pADH2-SoaA/B*), showing effects of combining fatty acid metabolism modulation with SoaA/B expression. Data are presented as mean ± s.d. (n = 3 biologically independent samples). Source data are provided as a Source Data file.

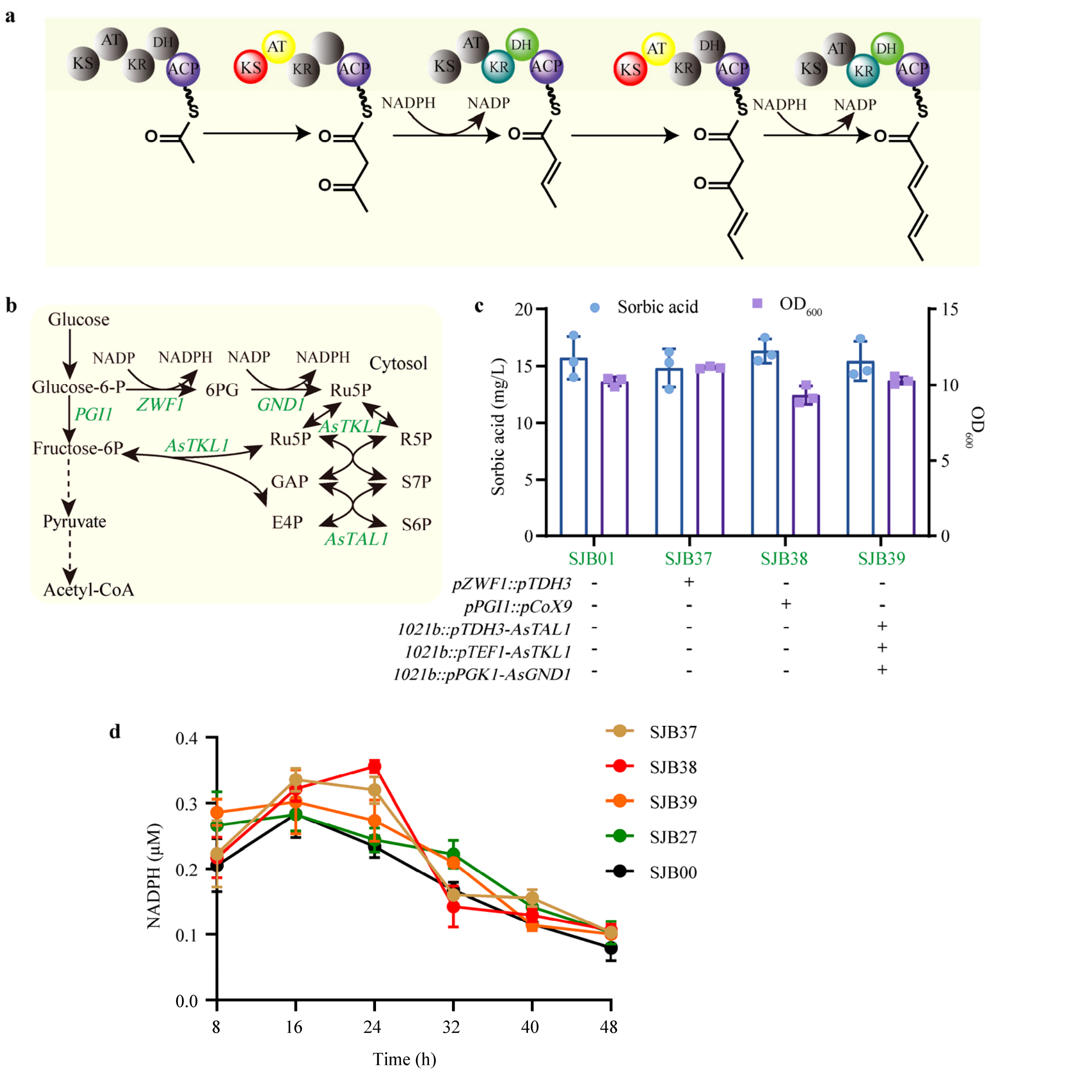

Supplementary Fig. 23. Engineering NADPH supply and central carbon metabolism for SA production. a, Schematic of enzymatic steps in a biosynthetic pathway involving acyl carrier protein (ACP) and NADPH-dependent reactions, with key domains (ketosynthase, KS; acyltransferase, AT; ketoreductase, KR; dehydratase, DH) highlighted. **b,** Metabolic map of central carbon metabolism and pentose phosphate pathway (PPP) in *S. cerevisiae*, showing engineered enzymes (e.g., ZWF1, GND1, AsTKL1, AsTAL1) and NADPH generation nodes. **c,** SA titers (blue bars) and cell density (OD_{600}, purple bars) in strains SJB01 (control) and engineered strains SJB37, SJB38, SJB39 with modifications targeting NADPH supply and PPP enzymes, cultivated to evaluate the impact on SA biosynthesis. **d**, Intracellular NADPH dynamics in engineered strains SJB37, SJB38, SJB39, and SJB27 during shake-flask fermentation. After cell disruption, samples were quantified by protein concentration, and NADPH content was determined following normalization to a uniform protein concentration. Data are presented as mean ± s.d. (n = 3 biologically independent samples). Source data are provided as a Source Data file.

a

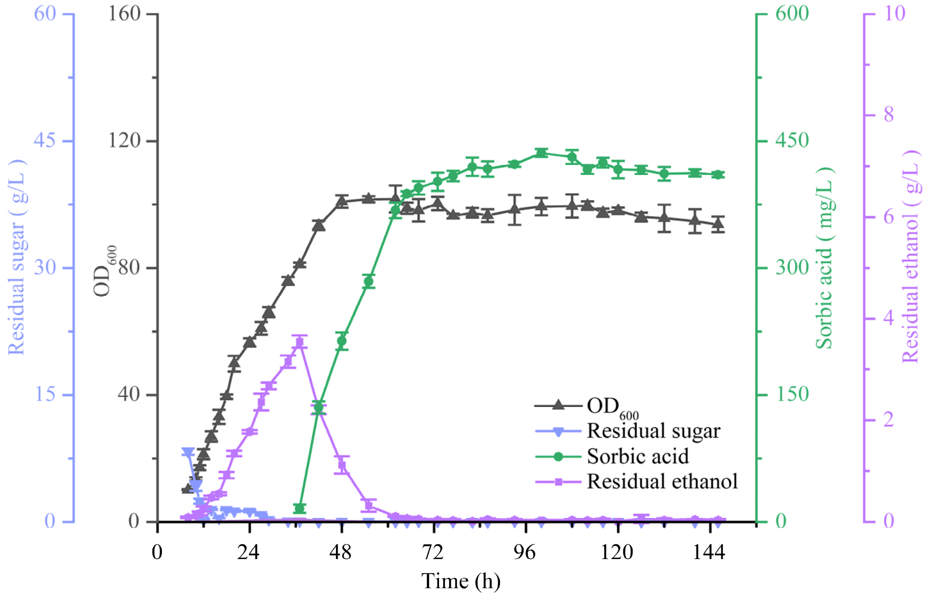

b

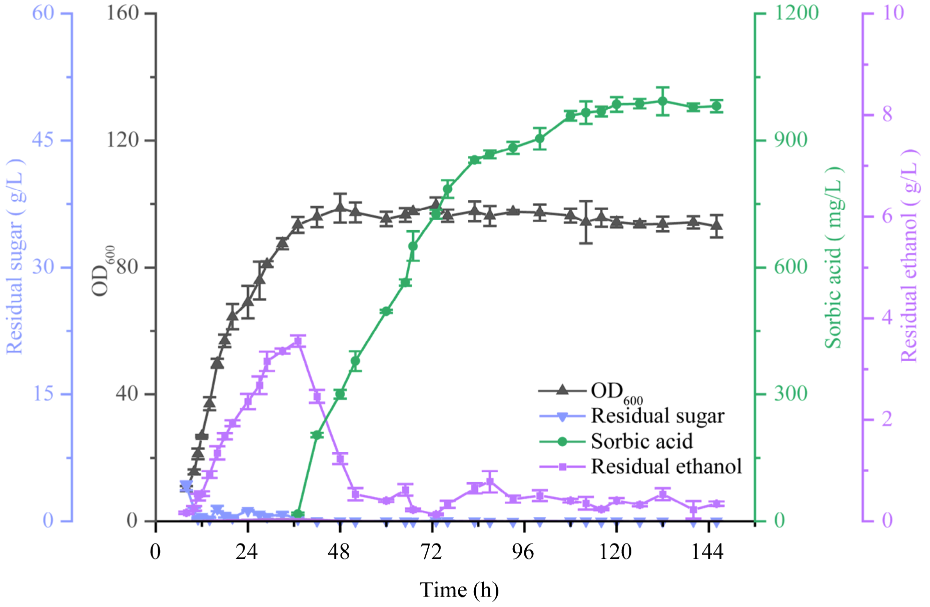

Supplementary Fig. 24. Time-course profiles of fermentation parameters in engineered *S. cerevisiae* cultures. a, Fermentation dynamics without exogenous ethanol induction, showing cell density (OD_{600}, black triangles), residual sugar concentration (blue triangles), sorbic acid titer (green circles), and residual ethanol concentration (purple circles) over 156 h. **b,** Fermentation dynamics with exogenous ethanol induction, with the same parameters plotted as in (**a**). Error bars represent standard deviations from biological replicates. Data are presented as mean ± s.d. (n = 3 biologically independent samples). Source data are provided as a Source Data file.

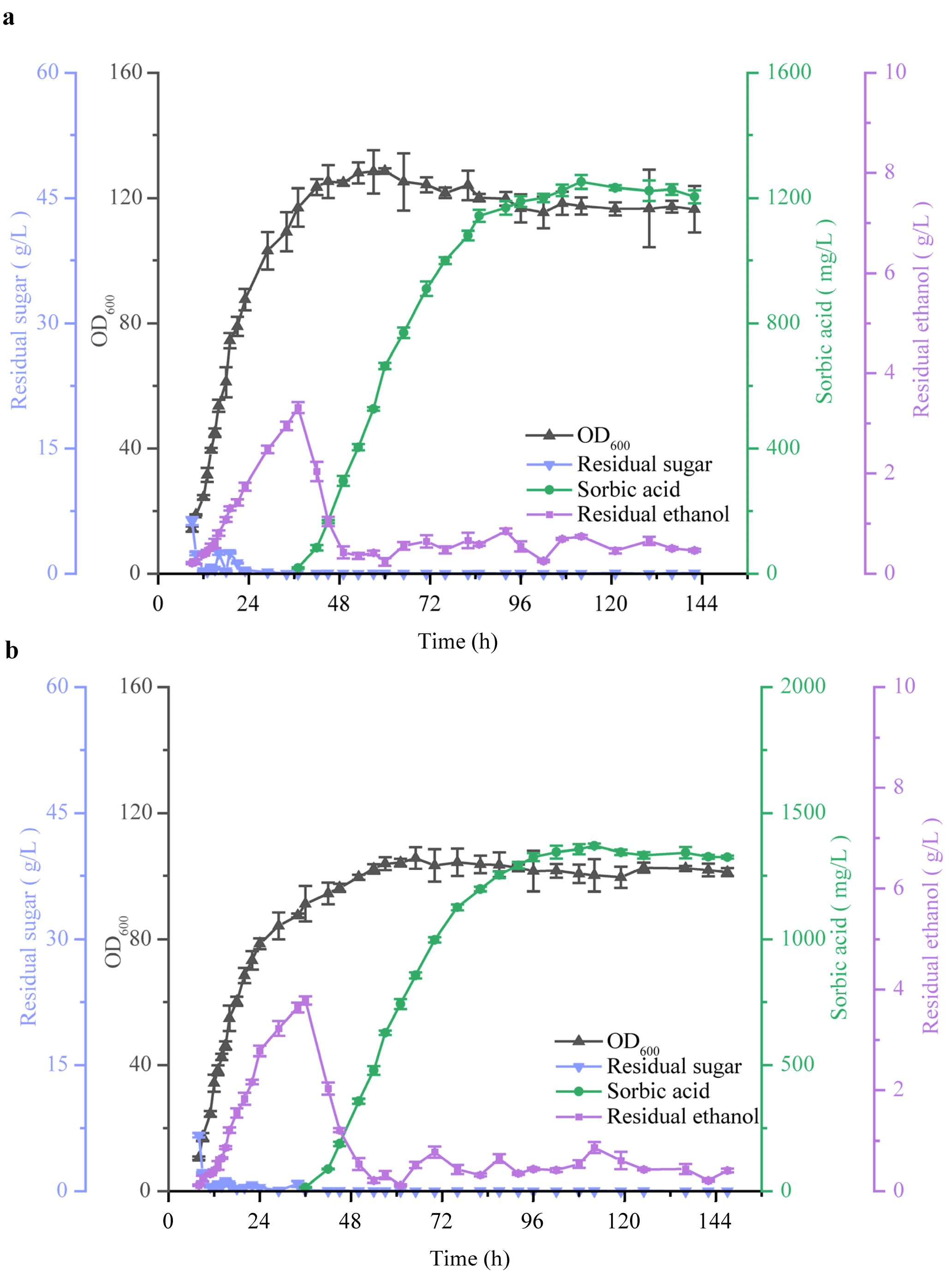

Supplementary Fig. 25. Time-course profiles of fermentation parameters during SA production by engineered *S. cerevisiae* under different pH conditions. a, Fermentation dynamics in pH6.0, showing cell density (OD_{600}, black triangles), residual sugar concentration (blue triangles), sorbic acid titer (green circles), and residual ethanol concentration (purple squares) over 156 h. **b**, Fermentation dynamics in pH7.0, with the same parameters plotted as in (**a**). Error bars represent standard deviations from biological replicates. Data are presented as mean ± s.d. (n = 3 biologically independent samples). Source data are provided as a Source Data file.

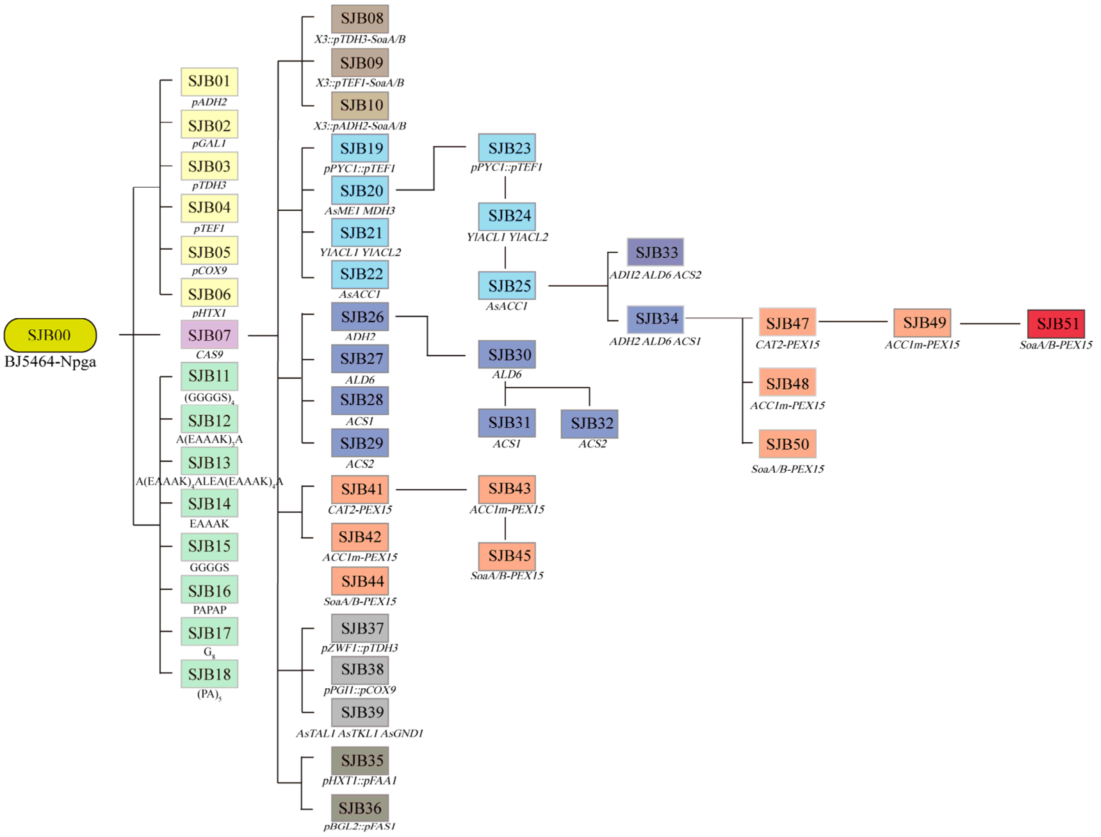

Supplementary Fig. 26. Engineering Schematic of a High-Yield SA-Producing Strain Constructed in *S. cerevisiae*. promoter engineering (yellow), SoaA–SoaB fusion protein engineering with different linkers (cyan), integration of SoaA and SoaB genes into the Saccharomyces cerevisiae genome (light brown), expansion of the cytosolic acetyl-CoA pool (light blue), enhancement of acetyl-CoA supply via ethanol utilization (purple), peroxisomal targeting of key enzymes (orange-red), augmentation of NADPH supply (dark brown), attenuation of fatty acid synthesis pathway (gray), and the final engineered strain (red).

Supplementary Tab. 1 NMR spectroscopic data of SA

^{1}H NMR spectrum (600 MHz), ^{13}C NMR spectrum (150 MHz)

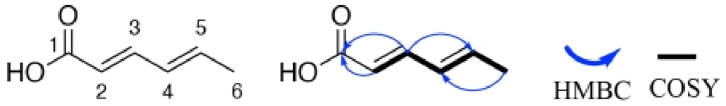

	SA (solved in CD_3OD)			
	**δ_H, mult (*J* in Hz)**	**δ_C**	**COSY**	**Key HMBC (H→C #)**
**1**		170.8, C		
**2**	5.73, d (15.3)	120.1, CH	H3	1
**3**	7.21, dd (15.3, 10.6)	146.9, CH	H2, H4	1, 5
**4**	6.22, m	131.0, CH	H3, H5	
**5**	6.15, m	140.6, CH	H4, H6	
**6**	1.81, d (6.6)	18.7, CH	H5	4

Supplementary Tab. 2 NMR spectroscopic data of SN

^{1}H NMR spectrum (600 MHz), ^{13}C NMR spectrum (150 MHz)

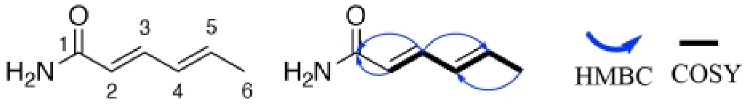

**Position**	SN (solved in CD_3OD)			
	**δ_H, mult (*J* in Hz)**	**δ_C**	**COSY**	**Key HMBC (H→C #)**
**1**		171.5, C		
**2**	5.94, d (15.2)	122.3, CH	H3	1
**3**	7.15, dd (15.3, 10.6)	143.2, CH	H2, H4	1, 5
**4**	6.24, m	131.0, CH	H3, H5	
**5**	6.15, m	139.2, CH	H4, H6	
**6**	1.86, d (6.6)	18.6, CH	H5	4

Supplementary Tab. 3 Antifungal activities of SA and SN.

IC_{50}	*Aspergillus. niger*	*Penicillum. digitorum*	*Aspergillus. nidulans*	*Canidia. albicans*	*Myrothecium* sp. FJNU 6
Sorbic acid (mM)	2.940	0.0979	0.2183	5.645	0.2513
sorbamide (mM)	NI	NI	NI	NI	NI
hygromycin (mM)	0.008	0.012	0.021	0.031	0.012

Supplementary Tab. 4. Amino acid sequences of linker peptides used in this study.

Linker	Application
Flexible	GGGGS
	$(GGGGS)_4$
	G_4
	G_8
Rigid	PAPAP
	$(PA)_5$
	$A(EAAAK)_4ALE\text{-}A(EAAAK)_4A$
	$A(EAAAK)_3A$

Eight linker peptides, representing both flexible and rigid types with variable lengths, were selected and fused between proteins to modulate their relative orientation and functional coupling.

Uncropped gels corresponding to Supplementary Figures

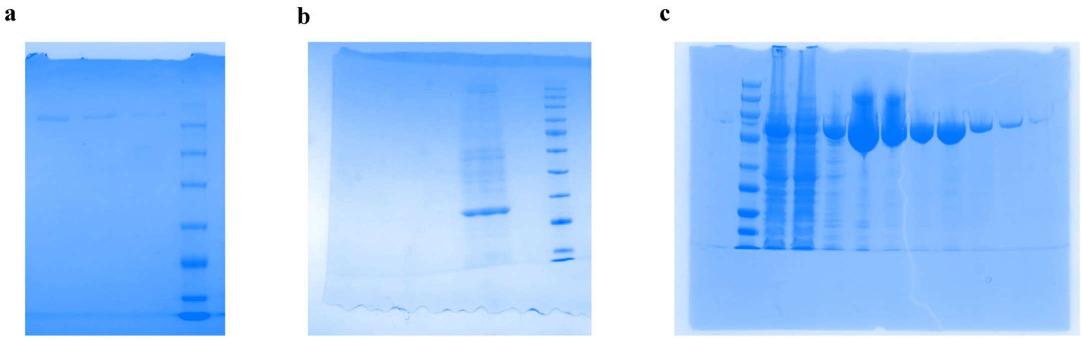

Uncropped gels corresponding to Supplementary Fig. 16. a Uncropped gel of SoaA. **b** Uncropped gel of SoaB. **b** Uncropped gel of SoaC.
