# Supplementary material for: Toward sustainable food preservatives: high-level production of sorbic acid in engineered Saccharomyces cerevisiae
Source: Nat Commun. 2026 Apr 21;17:5492. doi: 10.1038/s41467-026-72163-8 (PMC13284220; doi:10.1038/s41467-026-72163-8)
Supplement: Supplementary file 3 — Description of Additional Supplementary Files [file 41467_2026_72163_MOESM3_ESM.pdf]

Description of Additional Supplementary Files

Supplementary Data 1： List of primer sequences used in this study. All primer sequences were designed using SnapGene 6.0.2 software and synthesized by Fuzhou Shangya Biotechnology Co., Ltd.

Supplementary Data 2： Strain numbers and corresponding genetic information of the genetically engineered Saccharomyces cerevisiae strains used in this study.
