# Supplementary material for: Toward sustainable food preservatives: high-level production of sorbic acid in engineered Saccharomyces cerevisiae
Source: Nat Commun. 2026 Apr 21;17:5492. doi: 10.1038/s41467-026-72163-8 (PMC13284220; doi:10.1038/s41467-026-72163-8)
Supplement: Supplementary file 2 — Peer Review File [file 41467_2026_72163_MOESM2_ESM.pdf]

nature portfolio

Peer Review File

Toward sustainable food preservatives: high-level production of sorbic acid in engineered Saccharomyces cerevisiae

Corresponding Author: Professor Li Li

This file contains all reviewer reports in order by version, followed by all author rebuttals in order by version.

Version 0:

Reviewer comments:

Reviewer #1

(Remarks to the Author)
The study reports the identification of the biosynthetic gene cluster (BGC) responsible for sorbic acid (SA) formation, its heterologous reconstruction in S. cerevisiae, and subsequent strain and process engineering that achieved a production titer of 1.84 g/L. Sustainable and scalable bioproduction of SA as a food preservative is highly desirable. The characterization of the SA/SN BGC offers valuable insights into SA/SN biosynthesis and opens opportunities for developing sustainable alternatives to fossil fuel–derived production processes.

Major:
1, The authors claim to have uncovered the first microbial biosynthetic pathway for SA/SN formation. Detailed biochemical and functional characterization of the newly identified polyketide synthase (SoaA) would be highly valuable, as it would deepen our understanding of the pathway and elucidate the molecular mechanisms underlying SA/SN biosynthesis.
2, From 20 candidate PKS BGCs, BGC11.6 was identified as the strongest candidate for SA/SN biosynthesis through retro-biosynthetic analysis. A more detailed description of this analysis method is necessary to help readers understand the rationale behind the selection and would strengthen the impact of the study.
2, The authors initially characterized the BGC in A. nidulans, but subsequently selected S. cerevisiae as the production host. Because filamentous fungi are often regarded as highly suitable hosts for expressing fungal PKS BGC, the rationale for choosing yeast should be clarified. How does the production level in A. nidulans compare with that in S. cerevisiae prior to optimization?
3, The growth inhibition assays were performed across a pH range of 3–8. Since S. cerevisiae generally prefers acidic environments, it is unclear how the different pH conditions influenced growth, particularly at pH 7 and 8, which is not evident from Fig. S18. In addition, given the pKa of sorbic acid (4.76), pH values above 5 strongly favor the dissociated sorbate form. The difference in sorbate dissociation between pH 6 and 7 is not big, so the dramatic decrease in antifungal activity above pH 6 requires further explanation. Moreover, the IC50 at pH 7 is approximately 90 mM, which is not sufficient high for developing a high-level production process. How should this toxicity be considered when selecting the production host, and what strategies could be implemented to mitigate it?
4, The pADH2-driven expression on 2 µ plasmids yielded the highest biomass (OD = 10.2) and SA production (15.7 mg/L, Fig 4). In contrast, genomic integration driven by pADH2 reduced SA production to 8.3 mg/L (Fig S19), which is expected due to lower gene copy number. However, the biomass yield also decreased with genomic integration (OD = 9.3). If genomic integration is generally associated with reduced metabolic burden compared with plasmid expression, how do the authors explain this observation? Furthermore, the study used two 2 µ plasmids with Ura and Trp auxotrophic markers to drive SA biosynthesis gene expression, how did the authors ensure stable expression when culturing in YPD media, which lacks selection pressure?

Language and Grammar:
The manuscript requires thorough revision for grammar and language accuracy. A few examples include:
– L255: “effiecnt”
– L257: “such as S. cerevisiae is a potential strategy for high-level SA production”
– L507: “and engineering produced SA in S. cerevisiae”

Reviewer #2

(Remarks to the Author)
This manuscript reports the discovery of a previously unrecognized microbial biosynthetic pathway for sorbic acid (SA) and the successful development of a yeast-based fermentation process for its sustainable production. The work is timely and of potential industrial significance, particularly given the widespread use of SA as a food preservative and the current reliance on fossil-based chemical synthesis. The identification of the SA/sorbamide (SN) gene cluster and the demonstration of gram-scale production in a 50 L bioreactor represent notable advances. However, several questions should be addressed:
1.The authors report the co-production of sorbamide (SN) together with SA. The biological relevance of SN in the native producer remains unclear. Is SN a final metabolite, a storage form, or a shunt product of SA biosynthesis? Further discussion or experimental support would strengthen this point.

2.The identification of the SoaA–SoaB–SoaC gene cluster is central to this study. Please provide more details on how the functions of individual genes were experimentally validated, and whether potential redundancy or auxiliary genes within the cluster were assessed.

3.SoaA is described as a highly reducing polyketide synthase that produces a C6 unsaturated fatty acid. The mechanism by which SoaA achieves strict chain-length specificity is not fully addressed. Do the authors have insights into how premature termination or over-elongation is avoided?

4.The manuscript proposes SoaB as the thioesterase/hydrolase responsible for SA release. Is SoaB strictly required for product formation, and what happens to the polyketide intermediate in its absence? Additional clarification would improve mechanistic understanding.

5.SoaC is proposed to convert SA to SN. It remains unclear whether this reaction is reversible in vivo, or whether SN can be converted back to SA. Please clarify the enzymatic directionality and its implications for product distribution.

6.While S. cerevisiae is a well-established host, alternative microbes with higher native fatty acid flux may be more suitable for SA production. The authors should justify the choice of yeast over other potential chassis organisms.

7.The authors report improved production upon targeting the pathway to peroxisomes. More mechanistic explanation is needed regarding how peroxisomal localization enhances precursor availability, reduces toxicity, or minimizes competing pathways.

Reviewer #3

(Remarks to the Author)
This manuscript reports the first microbial biosynthesis of sorbic acid (SA) and sorbamide (SN). Guided by a self-protection mechanism-based retrosynthesis analysis, the authors rapidly identified the SA/SN biosynthetic gene cluster in Myrothecium sp. FJNU6. Subsequent systematic metabolic engineering, including the application of dynamic promoters, enhancement of acetyl/malonyl-CoA supply, peroxisomal compartmentalization, along with pH-controlled fed-batch fermentation strategies, achieved a high SA titer of 1.84 g/L in a 50L fermenter. Moreover, the utilization of pH optimization to mitigate SA toxicity during fermentation is a concise and efficient strategy that boosted production. Although the manuscript is acceptable after revision, I have several minor comments:
1. The NMR spectral peaks lack assignments, which should be provided.
2. In section “Characterization and bioactivity analysis of microbial SA and SN”, the antibacterial results of the extract of Myrothecium sp need to be presented.
3. In section “Promoter selection dictates SA production dynamics” pHXT1 was not a constitutive promoter; For the description “whereas pADH2, whose activity is repressed during glucose consumption and induced upon ethanol accumulation, alleviates growth-phase toxicity by delaying pathway expression.”, when pGAL1 was used, it is also repressed during glucose consumption and induced upon Galactose addition, and also alleviates growth-phase toxicity by delaying pathway expression, why the production was also limited? Additionally, why are the biomass levels of the integrated strains limited?
4. Section “Linker-mediated enzyme fusion fails to improve SA production” appears minimally impactful in its current form. Consider discussing it briefly within another relevant section rather than devoting a full section to it.
5. In section “Engineering ethanol utilization for enhanced acetyl-CoA and NADPH supply”, ALD6 is a key NADPH-regenerating enzyme in the cytosol; therefore, the observed production improvement could also relate to enhanced NADPH supply. Furthermore, after overexpression of Zwf1, Gnd1, Tkl1, and Tal1, confirming the change in NADPH levels would support the authors’ conclusion.
6. In the description “In shake-flask fermentations, SJB51 exhibited optimal growth at pH 6.0 (OD_{600} = 13.8), with SA titers reaching 305.3 mg/L. SA production increased with higher pH values and peaked at pH 7.0 (340 mg/L), although biomass decreased slightly (OD_{600} = 12.3).” Please clarify how pH stability was maintained during the shake-flask fermentations.
7. Could the authors comment on the commercial potential of this bio-manufacturing approach as an alternative to other production routes in terms of cost?
8. There are some writing mistakes:
In the sentence “The term “sorbic acid” traces its origin to its first isolation from the unripe berries of the mountain ash tree (Sorbus aucuparia) by A. W. Hoffman in the 18597. and then was commercially used as antimicrobial additive in the 1940s.” Replace the full stop in the original text ("18597. and then") with a comma after 1859.

In the sentence “It is challenging in overproduction of commercially valuable antimicrobial agent SA in its natural host Myrothecium sp. FJNU6, since SoaC effiecnt driven the bioconversion of SA toward SN biosynthesis.” Effiecnt should be efficiently

Version 1:

Reviewer comments:

Reviewer #1

(Remarks to the Author)
The authors have addressed the questions.

Reviewer #2

(Remarks to the Author)
The authors have thoroughly answered all my queries. I believe the manuscript is now suitable for publication and have no additional comments.

Reviewer #3

(Remarks to the Author)
The revised manuscript is acceptable for publication.

Open Access This Peer Review File is licensed under a Creative Commons Attribution 4.0 International License, which permits use, sharing, adaptation, distribution and reproduction in any medium or format, as long as you give appropriate credit to the original author(s) and the source, provide a link to the Creative Commons license, and indicate if changes were made.
In cases where reviewers are anonymous, credit should be given to 'Anonymous Referee' and the source.
The images or other third party material in this Peer Review File are included in the article’s Creative Commons license, unless indicated otherwise in a credit line to the material. If material is not included in the article’s Creative Commons license and your intended use is not permitted by statutory regulation or exceeds the permitted use, you will need to obtain permission directly from the copyright holder.
To view a copy of this license, visit https://creativecommons.org/licenses/by/4.0/

We sincerely thank the Editor and the Reviewers for their careful evaluation of our manuscript and for the constructive comments. The insightful suggestions have helped us improve the quality of our manuscript.

We have carefully revised the manuscript and addressed all concerns in detail. All revisions in the manuscript are shown using track changes for ease of evaluation. For clarity, the reviewers' comments are reproduced in black, and our responses are provided in blue below.

Below, we provide a point-by-point response to each comment.

Reviewer #1 (Remarks to the Author):

The study reports the identification of the biosynthetic gene cluster (BGC) responsible for sorbic acid (SA) formation, its heterologous reconstruction in *S. cerevisiae*, and subsequent strain and process engineering that achieved a production titer of 1.84 g/L. Sustainable and scalable bioproduction of SA as a food preservative is highly desirable. The characterization of the SA/SN BGC offers valuable insights into SA/SN biosynthesis and opens opportunities for developing sustainable alternatives to fossil fuel–derived production processes.

Major:

1, The authors claim to have uncovered the first microbial biosynthetic pathway for SA/SN formation. Detailed biochemical and functional characterization of the newly identified polyketide synthase (SoaA) would be highly valuable, as it would deepen our understanding of the pathway and elucidate the molecular mechanisms underlying SA/SN biosynthesis.

Response:

We thank the reviewer for this constructive suggestion. To strengthen the mechanistic basis of our study, we have now performed additional in vitro biochemical characterization of SoaA and SoaB (**Line 218-232**). His-tagged SoaA and SoaB were heterologously expressed and purified, and enzymatic assays were conducted using acetyl-CoA, malonyl-CoA, and NADPH as substrates. While no free product was detected in reactions containing SoaA alone, alkaline hydrolysis released sorbic acid, consistent with formation of an ACP-bound intermediate. The hydrolyzed product corresponded to a triketide-derived SA, with no longer-chain products detected. Inclusion of SoaB enabled direct enzymatic release of SA without chemical treatment. These new data are presented in revised Fig. 3a and described in the revised manuscript.

Together, these experiments provide direct biochemical evidence for the catalytic role of SoaA in triketide assembly and clarify the function of SoaB in product release.

For clarity, the revised **Fig. 3** incorporating the newly added SoaA and SoaB biochemical data is provided below.

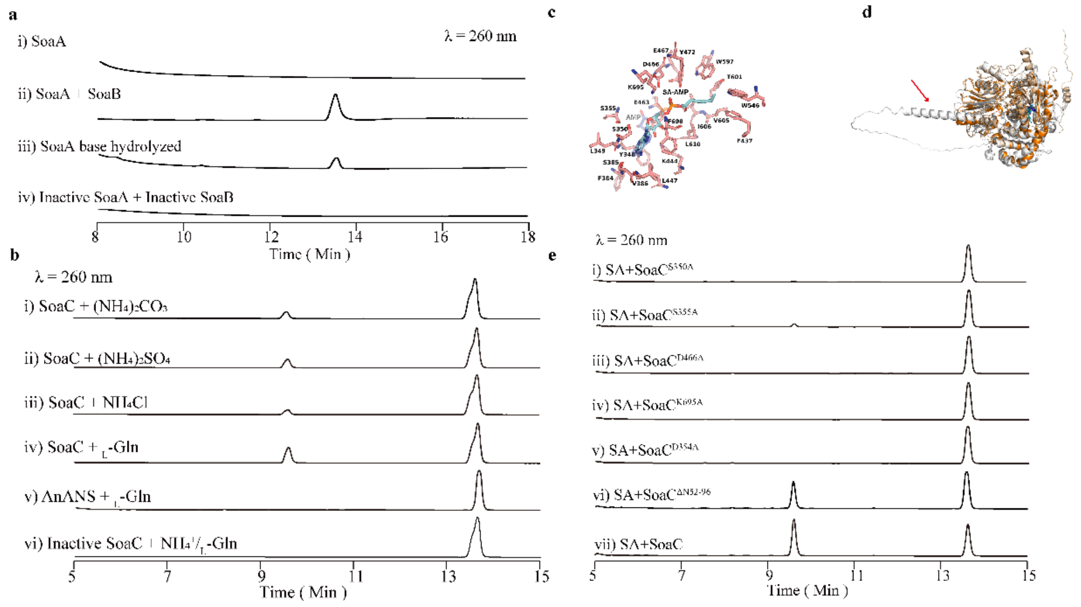

Revised **Fig. 3**. Biochemical characterization of the *soa* pathway enzymes

The in vitro assay testing whether SoaC catalyzes asparagine formation has been relocated from **Fig. 3** to Supplementary **Fig. S16** for clarity and improved figure organization,

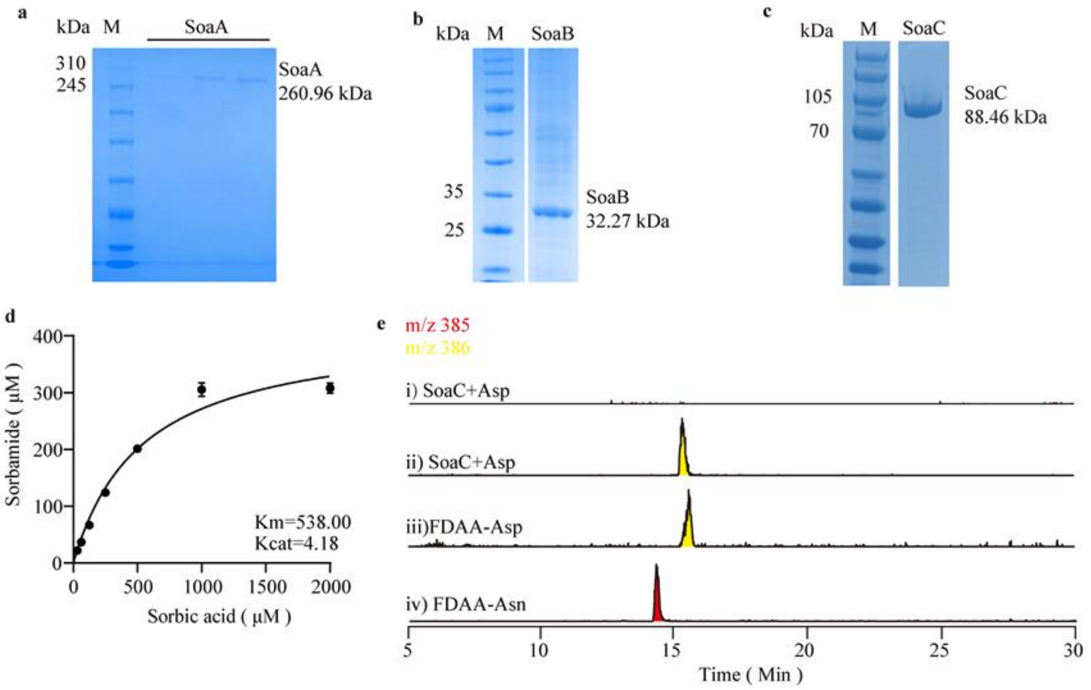

Fig. S16 Characterization of SoaA/B/C and SoaC enzymatic kinetics.

2, From 20 candidate PKS BGCs, BGC11.6 was identified as the strongest candidate for SA/SN biosynthesis through retro-biosynthetic analysis. A more detailed description of this analysis method is necessary to help readers understand the rationale behind the selection and would strengthen the impact of the study.

Response:

We thank the reviewer for this helpful suggestion. To clarify the rationale underlying cluster selection, we have revised the corresponding paragraph to explicitly describe the evaluation criteria applied to the 20 PKS-containing BGCs.

These PKS clusters were systematically evaluated based on (i) the presence of an HR-PKS capable of iterative chain elongation and programmed reduction/dehydration steps, (ii) the presence of a gene encoding a putative hydrolase or product-release enzyme, and (iii) the co-localization of an amidotransferase candidate capable of catalyzing SA amidation. Among the 20 PKS-containing BGCs, only BGC11.6 satisfied all three criteria and was therefore designated the *soa* cluster. (**Line 173-179**).

We believe this clarification improves the transparency of the retro-biosynthetic analysis and strengthens the justification for selecting BGC11.6.

3, The authors initially characterized the BGC in A. nidulans, but subsequently selected S. cerevisiae as the production host. Because filamentous fungi are often regarded as highly suitable hosts for expressing fungal PKS BGC, the rationale for choosing yeast should be clarified. How does the production level in A. nidulans compare with that in S. cerevisiae prior to optimization?

Response:

We thank the reviewer for this important comment. *A. nidulans* was initially employed as a heterologous host for pathway validation, as filamentous fungi are well suited for expression of fungal PKS gene clusters. Under solid-state fermentation conditions, expression of the *soa* cluster in *A. nidulans* resulted in production of SA and SN at 0.81 mg/L and 0.50 mg/L, respectively These data have been included in the manuscript (**Line 202–203**). In contrast, only trace amounts were detected under liquid fermentation conditions. Solid-state fermentation is commonly employed for heterologous expression of fungal BGCs in filamentous fungi, as liquid cultivation often yields substantially lower titers.

In *S. cerevisiae*, initial expression of the *soa* cluster during promoter screening yielded 15.7 mg/L SA under the control of pADH2 prior to further metabolic optimization. These results are presented in **Fig. 4b**.

While filamentous fungi provide a suitable platform for pathway verification, the primary objective of this work is to establish a scalable and industrially translatable production route for food-grade SA. *S. cerevisiae* supports robust liquid fermentation, offers extensive genetic tractability, and possesses GRAS status, which are important considerations for

food-related applications. For these translational and regulatory reasons, *S. cerevisiae* was selected as the chassis for subsequent engineering and scale-up studies. We have clarified this rationale in the revised manuscript (**Line 284–290**).

4, The growth inhibition assays were performed across a pH range of 3–8. Since S. cerevisiae generally prefers acidic environments, it is unclear how the different pH conditions influenced growth, particularly at pH 7 and 8, which is not evident from Fig. S18. In addition, given the pKa of sorbic acid (4.76), pH values above 5 strongly favor the dissociated sorbate form. The difference in sorbate dissociation between pH 6 and 7 is not big, so the dramatic decrease in antifungal activity above pH 6 requires further explanation. Moreover, the IC50 at pH 7 is approximately 90 mM, which is not sufficient high for developing a high-level production process. How should this toxicity be considered when selecting the production host, and what strategies could be implemented to mitigate it?

Response:

We thank the reviewer for these insightful comments regarding pH-dependent toxicity.

S. cerevisiae generally grows optimally under mildly acidic conditions. We acknowledge that the effect of different pH conditions on *S. cerevisiae* growth, especially at pH 7 and 8, was not clearly presented in the original **Fig. S18**. To address this concern, we have added YPD control experiments conducted at different pH values in the revised Supplementary Information (**Fig. S19b**) to explicitly evaluate the effect of pH on cell growth. These data clarify the growth behavior across the tested pH range. During the experiments, culture pH was monitored and adjusted with 2 M NaOH or 2% H_2SO_4 as required. The relevant description has been incorporated into the revised manuscript (**Line 300**).

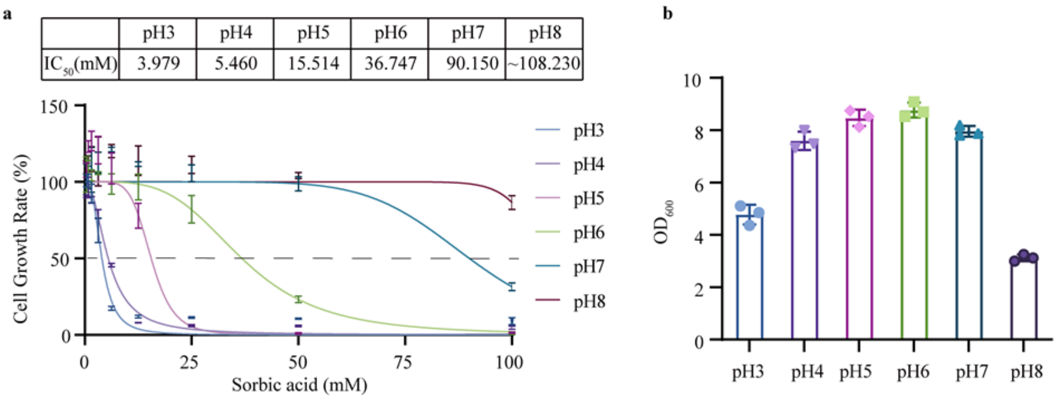

Fig. S19. Effect of SA concentration on cell growth rate of *S. cerevisiae* at different pH values.

Regarding the apparent decrease in antifungal activity above pH 6, SA exerts its inhibitory effect primarily in its undissociated form, which can diffuse across the plasma membrane and acidify the cytosol. Although the numerical difference in dissociation between pH 6

and 7 may appear modest, the fraction of undissociated SA decreases exponentially with increasing pH according to the Henderson–Hasselbalch relationship. This reduction substantially limits intracellular accumulation and thereby sharply attenuates antifungal activity at higher pH values.

With respect to the IC_{50} (~90 mM at pH 7), we agree that product toxicity represents a key consideration for high-level production. Notably, the maximum SA concentration achieved in this study (1.84 g/L, corresponding to ~16 mM) remains substantially below the measured IC_{50} value. Therefore, under our current fermentation conditions, SA accumulation does not approach inhibitory levels.

Nevertheless, toxicity is expected to become increasingly relevant as titers increase. We thank the reviewer for raising this important point. While no severe toxicity effects were observed under the current production conditions, product-associated toxicity may become increasingly relevant at higher titers. Future optimization strategies could therefore include enhanced export capacity, adaptive laboratory evolution, dynamic pathway regulation, and process intensification approaches such as in situ product removal to mitigate toxicity and facilitate further development toward industrial implementation. These aspects are discussed in the revised Discussion section (**Line 623-629**).

5, The pADH2-driven expression on 2 μ plasmids yielded the highest biomass (OD = 10.2) and SA production (15.7 mg/L, Fig 4). In contrast, genomic integration driven by pADH2 reduced SA production to 8.3 mg/L (Fig S19), which is expected due to lower gene copy number. However, the biomass yield also decreased with genomic integration (OD = 9.3). If genomic integration is generally associated with reduced metabolic burden compared with plasmid expression, how do the authors explain this observation? Furthermore, the study used two 2 μ plasmids with Ura and Trp auxotrophic markers to drive SA biosynthesis gene expression, how did the authors ensure stable expression when culturing in YPD media, which lacks selection pressure?

Response:

We thank the reviewer for this important comment. We agree that genomic integration is generally expected to reduce plasmid-associated metabolic burden. However, chromosomal integration can also introduce position-dependent effects that may influence host physiology. In the present study, the integrated strain exhibited the anticipated reduction in SA production due to lower gene copy number, whereas the decrease in biomass was modest (OD_{600} values of 10.2 and 9.3 for plasmid-based and integrated strains, respectively), and may fall within normal biological variation observed in shake-flask cultures.

In addition, we cannot exclude the possibility that chromosomal insertion events or altered pathway balance under lower expression levels may impose a slight fitness cost, for example through accumulation of inhibitory intermediates or subtle perturbations to central metabolism.

Regarding plasmid stability, prior to production cultivation, seed cultures were propagated under selective conditions to ensure plasmid maintenance. A relatively high inoculation ratio (10%) was then used to rapidly reach the stationary phase, thereby minimizing the number of generations and reducing the likelihood of plasmid loss. These experimental details have now been clarified in the MM section (**Line 180-181**).

Language and Grammar:

The manuscript requires thorough revision for grammar and language accuracy. A few examples include:

– L255: “effiecnt”

– L257: “such as S. cerevisiae is a potential strategy for high-level SA production”

– L507: “and engineering produced SA in S. cerevisiae”

Response:

We thank the reviewer for pointing out these language issues. We have carefully revised the entire manuscript to improve grammar, clarity, and consistency. The specific errors highlighted by the reviewer (e.g., “effiecnt,” “such as *S. cerevisiae* is a potential strategy for high-level SA production,” and “and engineering produced SA in S. cerevisiae”) have been corrected in the revised version.

Reviewer #2 (Remarks to the Author):

This manuscript reports the discovery of a previously unrecognized microbial biosynthetic pathway for sorbic acid (SA) and the successful development of a yeast-based fermentation process for its sustainable production. The work is timely and of potential industrial significance, particularly given the widespread use of SA as a food preservative and the current reliance on fossil-based chemical synthesis. The identification of the SA/sorbamide (SN) gene cluster and the demonstration of gram-scale production in a 50 L bioreactor represent notable advances. However, several questions should be addressed:

1.The authors report the co-production of sorbamide (SN) together with SA. The biological relevance of SN in the native producer remains unclear. Is SN a final metabolite, a storage form, or a shunt product of SA biosynthesis? Further discussion or experimental support would strengthen this point.

Response:

We thank the reviewer for this insightful question. The biological role of SN in the native producer is indeed an important consideration. Based on our biochemical and structural analyses, SoaC functions as a dedicated sorbamide synthetase with specificity toward SA, suggesting that SN formation is a programmed enzymatic step rather than a spontaneous side reaction.

Given that SA possesses antifungal activity, we propose that conversion of SA to SN may serve as a self-protection mechanism in *Myrothecium* sp. FJNU6, potentially reducing intracellular toxicity by temporarily masking the carboxylic acid functionality. This hypothesis is consistent with the presence of a dedicated amidotransferase gene within the biosynthetic gene cluster and parallels other secondary metabolite pathways in which modifying enzymes mitigate product toxicity.

At present, it remains unclear whether SN represents a final metabolite, a storage form, or a regulatory intermediate in vivo. we have added a brief discussion in the revised manuscript (**Line 247-251**). Further studies will be required to fully resolve the physiological role of SN.

2.The identification of the SoaA–SoaB–SoaC gene cluster is central to this study. Please provide more details on how the functions of individual genes were experimentally validated, and whether potential redundancy or auxiliary genes within the cluster were assessed.

Response:

We thank the reviewer for emphasizing the importance of comprehensive functional validation of the *soa* gene cluster.

The roles of SoaA, SoaB, and SoaC were experimentally validated through stepwise heterologous reconstruction and in vitro biochemical assays. In the original submission, in vitro characterization of SoaC had already established its ATP-dependent amidation activity, demonstrating specific conversion of sorbic acid (SA) to sorbamide (SN).

In the revised manuscript, we have now added in vitro biochemical validation of SoaA and SoaB to further strengthen the mechanistic framework (**Line 222-237**). Expression of *soaA* alone in the heterologous host did not yield detectable free products, consistent with the absence of an intrinsic product-release domain. Co-expression of *soaA* and *soaB* resulted in production of SA, demonstrating that SoaB functions as a trans-acting hydrolase required for product release. Further addition of SoaC enabled conversion of SA to SN, confirming its role in amidation.

In vitro reconstitution assays further supported these assignments. When purified SoaA was incubated with acetyl-CoA, malonyl-CoA, and NADPH in the absence of SoaB, no free SA was detected. However, alkaline hydrolysis of the reaction mixture released SA as the sole product. These results indicate that SoaA synthesizes a triketide intermediate that remains tethered to the acyl carrier protein (ACP) domain in the absence of a dedicated release mechanism. Inclusion of SoaB enabled enzymatic release of SA without chemical treatment, confirming its function in product offloading. Together, these data establish soaA–soaB–soaC as the minimal and sufficient gene set for SA/SN biosynthesis. **Fig. 2** illustrates the in vivo functional reconstitution of SoaA–SoaB–SoaC, while the revised **Fig. 3** provides biochemical validation of all three enzymes' activities in vitro.

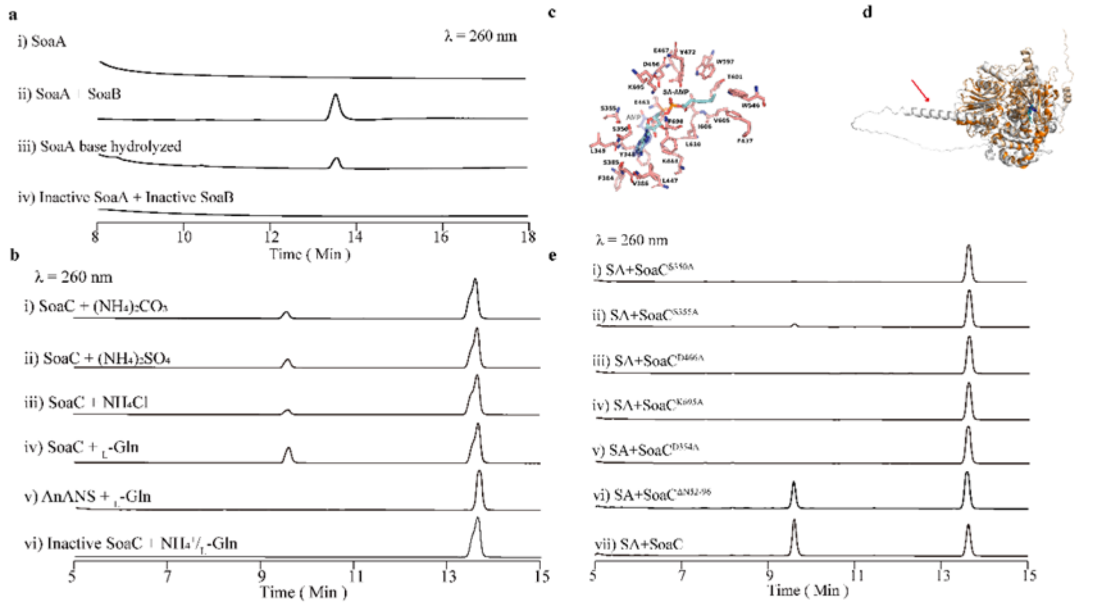

Fig.3. Characterization of SoaA/SoaB/SoaC activity in vitro.

Regarding potential redundancy or auxiliary genes, the soa cluster also contains genes encoding a cytochrome P450 and a cytochrome b5 protein. However, heterologous reconstruction of the minimal soaA–soaB–soaC gene set was sufficient to reconstitute SA/SN production, indicating that these additional genes are not required for core SA formation under the conditions tested. In the heterologous expression experiments, we additionally introduced the P450 and cytochrome b5 genes; however, no new products were detected under the tested conditions.

3.SoaA is described as a highly reducing polyketide synthase that produces a C6 unsaturated fatty acid. The mechanism by which SoaA achieves strict chain-length specificity is not fully addressed. Do the authors have insights into how premature termination or over-elongation is avoided?

Response:

We sincerely thank the reviewer for raising this important mechanistic question. We agree that chain-length specificity is a central issue in fungal HR-PKSs. In the original submission, this aspect was not directly addressed through biochemical evidence. In response to the reviewer’s suggestion, we have performed additional in vitro reconstitution and alkaline hydrolysis experiments in the revised manuscript to clarify the programming mechanism of SoaA (**Fig. 3**, revised).

Specifically, when purified SoaA was incubated with acetyl-CoA, malonyl-CoA, and NADPH in the absence of SoaB, no free product was detected. However, alkaline hydrolysis of the reaction mixture released SA as the sole detectable product, with no

longer-chain intermediates observed. These results demonstrate that SoaA synthesizes a triketide intermediate and does not undergo over-elongation under the tested conditions, indicating intrinsic chain-length programming.

Chain-length control in fungal HR-PKS systems can be influenced by partnering thiohydrolases, as demonstrated by Zabala et al. (ACS Chem. Biol. 2014, 9, 1576–1586). However, in our system, the newly added biochemical data support the conclusion that triketide specificity is established at the level of SoaA catalysis, while SoaB primarily mediates product release. We appreciate the reviewer's comment, which has allowed us to strengthen the mechanistic clarity of the manuscript.

4.The manuscript proposes SoaB as the thioesterase/hydrolase responsible for SA release. Is SoaB strictly required for product formation, and what happens to the polyketide intermediate in its absence? Additional clarification would improve mechanistic understanding.

Response:

We sincerely thank the reviewer for this insightful question. We agree that clarifying whether SoaB is strictly required for SA release, and elucidating the fate of the polyketide intermediate in its absence, are essential for a comprehensive mechanistic understanding.

Our heterologous expression experiments in *A. nidulans* provide strong genetic evidence that SoaB is required for efficient SA production. Expression of *soaA* alone did not result in detectable levels of free SA. In contrast, co-expression of *soaA* and *soaB* led to robust SA formation. Given that *A. nidulans* contains numerous endogenous hydrolases and thioesterases, if SA release occurred via non-specific hydrolysis of the ACP-bound intermediate, one would expect at least trace amounts of free SA in the SoaA-only strain. However, no SA was detected under these conditions. This observation strongly suggests that spontaneous or non-specific hydrolysis does not significantly contribute to product release and supports the role of SoaB as the dedicated thioesterase.

Importantly, in response to the reviewer's comment, we have now complemented these genetic data with new in vitro biochemical evidence. In the revised manuscript (**Fig. 3**), we demonstrate that purified SoaB is capable of hydrolytically releasing SA from the enzyme-bound intermediate generated by SoaA. In the absence of SoaB, no detectable free SA was observed, whereas addition of SoaB led to efficient formation of free SA. These results directly confirm the hydrolytic activity of SoaB and substantiate its role in product offloading.

Regarding the fate of the intermediate in the absence of SoaB, our data indicate that the polyketide remains enzyme-bound and does not undergo significant spontaneous release. No alternative shunt products were detected.

We have revised the Results sections (**Line 222-232**) and added the new in vitro data (**Fig. 3**) to clarify this point. We believe these additions significantly strengthen the mechanistic interpretation of the SoaA–SoaB functional pairing.

5.SoaC is proposed to convert SA to SN. It remains unclear whether this reaction is reversible in vivo, or whether SN can be converted back to SA. Please clarify the enzymatic directionality and its implications for product distribution.

Response：

We thank the reviewer for this important question.

If the SA-to-SN conversion were reversible, it could potentially function as a dynamic buffering mechanism to regulate intracellular SA levels. However, our experimental data do not support such a model.

In vitro assays demonstrate that SoaC catalyzes the amidation of SA to SN in an effectively unidirectional manner under the tested conditions. SN could not be converted back to SA. Consistently, in vivo feeding experiments in yeast showed that exogenously supplied SN was not converted to detectable SA. These results indicate that the SoaC-catalyzed reaction is effectively irreversible under physiological conditions.

Accordingly, the observed SA:SN ratio does not arise from a reversible equilibrium. Instead, it reflects metabolic flux partitioning at an irreversible branch point, where product distribution is determined by the relative rates of SA biosynthesis and its conversion to SN. Thus, SoaC does not operate as a dynamic equilibrium-based self-protection system, but rather as a flux-diverting enzyme controlling pathway output. Nevertheless, by channeling SA into SN, this reaction likely contributes to cellular protection through net reduction of intracellular SA levels.

6.While S. cerevisiae is a well-established host, alternative microbes with higher native fatty acid flux may be more suitable for SA production. The authors should justify the choice of yeast over other potential chassis organisms.

Response：

We thank the reviewer for this valuable and forward-looking suggestion.

We agree that alternative hosts with higher native fatty acid flux, such as *Yarrowia lipolytica* or certain bacterial systems, may offer advantages for future high-level SA production.

In this study, our objective was to establish and validate the microbial SA/SN biosynthetic pathway in a genetically stable and industrially relevant system. *S. cerevisiae* was selected because it is a widely used GRAS organism with extensive industrial application history and well-established regulatory acceptance. Importantly, a few studies have demonstrated that *S. cerevisiae* is a reliable platform for functional expression and reconstitution of fungal polyketide synthases.

These features make *S. cerevisiae* a suitable system for initial pathway validation and translational development. We have clarified this rationale in the revised manuscript (**Line**

286-290). Guided by the reviewer's suggestion, future work will evaluate alternative chassis organisms with higher fatty acid flux and enhanced weak-acid tolerance to further improve SA production levels.

7.The authors report improved production upon targeting the pathway to peroxisomes. More mechanistic explanation is needed regarding how peroxisomal localization enhances precursor availability, reduces toxicity, or minimizes competing pathways.

Response:

We thank the reviewer for this insightful comment. We agree that the positive effect of peroxisomal targeting warrants further mechanistic discussion.

In the revised manuscript (**Line 583-591**), we have expanded the Discussion to elaborate on several plausible mechanisms underlying the enhanced SA production upon peroxisomal localization.

First, peroxisomes are central organelles for fatty acid metabolism and β-oxidation, and are enriched in acetyl-/acyl-CoA intermediates with active CoA turnover. Targeting the SoaA–SoaB pathway to the peroxisome may increase the local availability of precursors required for polyketide chain assembly, thereby promoting SA biosynthesis.

Second, spatial compartmentalization may reduce competition with cytosolic malonyl-CoA-consuming pathways, such as fatty acid biosynthesis, effectively channeling metabolic flux toward SA production.

Third, as SA is a weak acid capable of causing intracellular acidification and toxicity, sequestration within the peroxisomal compartment may limit cytosolic exposure and partially alleviate product-associated stress.

We acknowledge that further quantitative metabolite analysis and subcellular flux measurements will be required to fully elucidate these mechanisms, and we have identified this as an important direction for future investigation.

Reviewer #3 (Remarks to the Author):

This manuscript reports the first microbial biosynthesis of sorbic acid (SA) and sorbamide (SN). Guided by a self-protection mechanism-based retrosynthesis analysis, the authors rapidly identified the SA/SN biosynthetic gene cluster in Myrothecium sp. FJNU6. Subsequent systematic metabolic engineering, including the application of dynamic promoters, enhancement of acetyl/malonyl-CoA supply, peroxisomal compartmentalization, along with pH-controlled fed-batch fermentation strategies, achieved a high SA titer of 1.84 g/L in a 50L fermenter. Moreover, the utilization of pH optimization to mitigate SA toxicity during fermentation is a concise and efficient strategy that boosted production. Although the manuscript is acceptable after revision, I have several minor comments:

1. The NMR spectral peaks lack assignments, which should be provided.

Response:

We thank the reviewer for pointing this out. We agree that peak assignment is essential for accurate interpretation of NMR data. In the revised Supporting Information, we have now provided complete ^{13}C NMR signal assignments for both SA and SN. Major peaks have been directly annotated on the NMR spectra (**Fig.S3 and S9**), and the corresponding chemical shift assignments have been summarized in **Tables S1 and S2**.

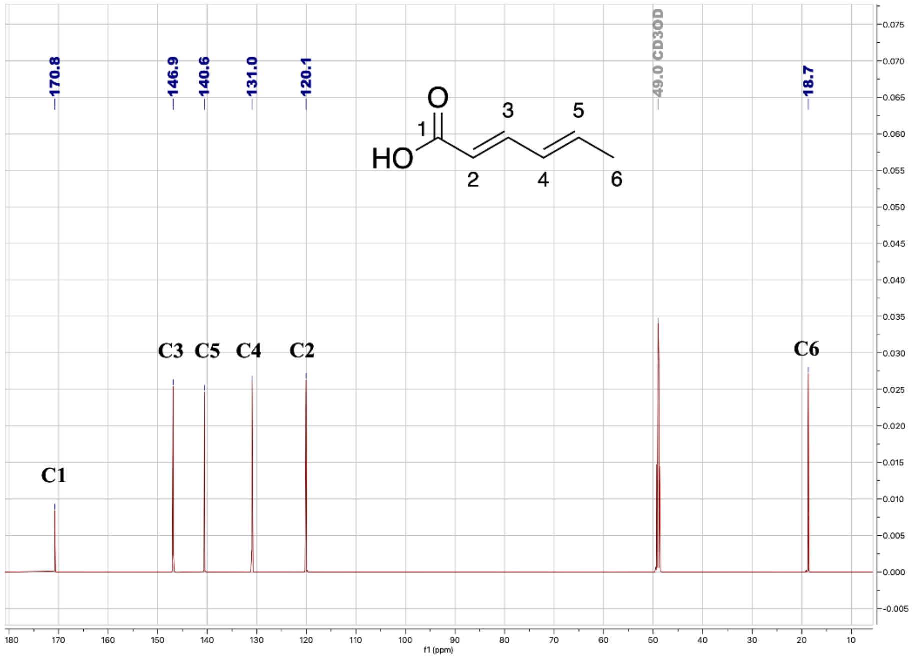

Fig. S3. ^{13}C NMR spectrum of sorbic acid in CD_3OD.

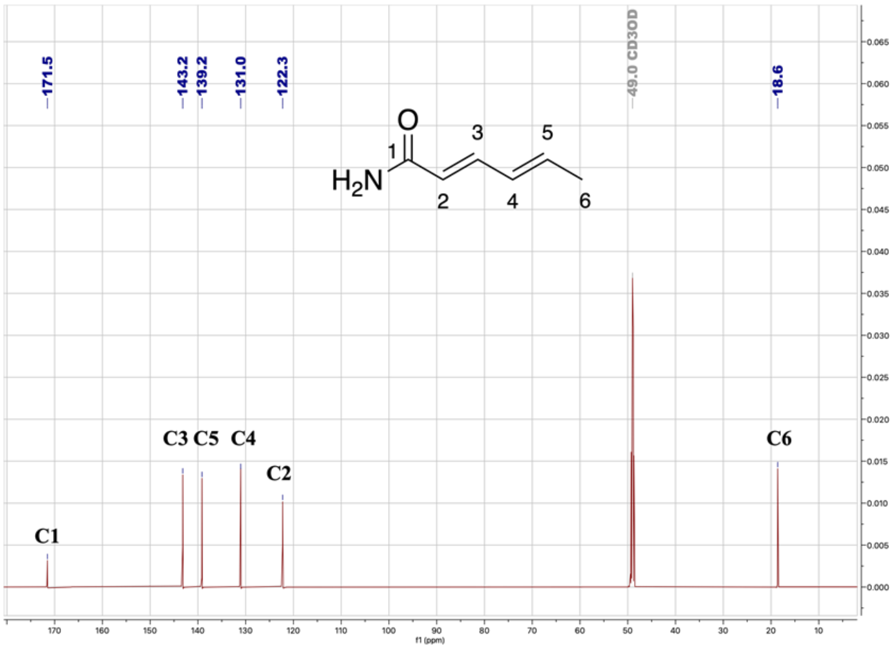

Fig. S9. ^{13}C NMR spectrum of sorbamide in CD_3OD.

Tab. S1 NMR spectroscopic data of SA

^{1}H NMR spectrum (600 MHz), ^{13}C NMR spectrum (150 MHz)

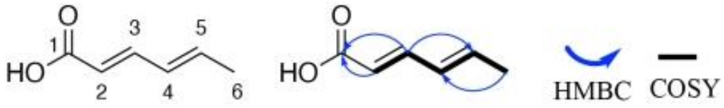

	SA (solved in CD_3OD)			
	**δ_H, mult (J in Hz)**	**δ_C**	**COSY**	**Key HMBC (H→C #)**
**1**		170.8, C		
**2**	5.73, d (15.3)	120.1, CH	H3	1
**3**	7.21, dd (15.3, 10.6)	146.9, CH	H2, H4	1, 5
**4**	6.22, m	131.0, CH	H3, H5	
**5**	6.15, m	140.6, CH	H4, H6	
**6**	1.81, d (6.6)	18.7, CH	H5	4

Tab. S2 NMR spectroscopic data of SN

^{1}H NMR spectrum (600 MHz), ^{13}C NMR spectrum (150 MHz)

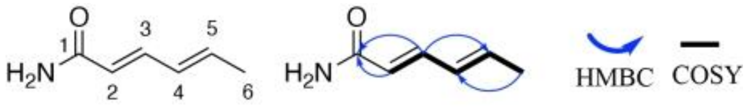

Position	SN (solved in CD_3OD)			
	**δ_H, mult (*J* in Hz)**	**δ_C**	**COSY**	**Key HMBC (H→C #)**
**1**		171.5, C		
**2**	5.94, d (15.2)	122.3, CH	H3	1
**3**	7.15, dd (15.3, 10.6)	143.2, CH	H2, H4	1, 5
**4**	6.24, m	131.0, CH	H3, H5	
**5**	6.15, m	139.2, CH	H4, H6	
**6**	1.86, d (6.6)	18.6, CH	H5	4

2. In section “Characterization and bioactivity analysis of microbial SA and SN”, the antibacterial results of the extract of Myrothecium sp need to be presented.

Response:

We thank the reviewer for this helpful suggestion. We agree that including the antibacterial activity data of the native *Myrothecium* sp. extract strengthens the bioactivity analysis. In the revised manuscript, we have added the antibacterial assay results of the crude extract from the natural producer strain, which are now presented in **Fig. S1** of the Supporting Information.

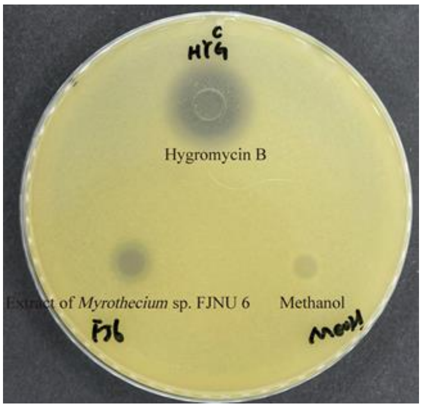

Fig. S1 Anti-*Candida* activity of *Myrothecium* sp. FJNU6 extract.

3. In section "Promoter selection dictates SA production dynamics" pHXT1 was not a constitutive promoter; For the description "whereas pADH2, whose activity is repressed during glucose consumption and induced upon ethanol accumulation, alleviates growth-phase toxicity by delaying pathway expression.", when pGAL1 was used, it is also repressed during glucose consumption and induced upon Galactose addition, and also alleviates growth-phase toxicity by delaying pathway expression, why the production was also limited? Additionally, why are the biomass levels of the integrated strains limited?

Response:

We thank the reviewer for this careful and important comment. We agree that clarification of promoter characteristics and production dynamics is necessary.

First, we have corrected the description of *pHXT1* in the revised manuscript (**Line 307, 310**). *pHXT1* is not a constitutive promoter but a glucose-responsive hexose transporter promoter regulated by extracellular glucose concentration.

Regarding the comparison between *pADH2* and *pGAL1*, we performed additional fermentation experiments to further clarify their production behaviors. In our initial *pGAL1* experiments, galactose was used as the sole carbon source, which resulted in early induction of SA biosynthesis during the growth phase. To systematically evaluate promoter performance, we subsequently conducted shake-flask fermentations using (i) glucose as the sole carbon source, (ii) galactose as the sole carbon source, (iii) glucose and galactose as dual carbon sources, and (iv) staged induction, where cells were first grown on glucose and galactose was added after 24 h as an inducer.

Our results (**Fig. S20**) showed that when glucose was used as the sole carbon source, the *pGAL1* strain (SJB02) achieved higher biomass than the *pADH2* strain (SJB01), but no detectable SA production was observed due to glucose repression of *pGAL1*. Under dual-carbon or staged induction conditions, SA production was detected in both strains; however, *pADH2* consistently yielded higher SA titers than *pGAL1*. This suggests that although *pGAL1* enables inducible expression, its induction dynamics and metabolic burden under galactose conditions may not be optimal for balanced SA biosynthesis in this system.

Regarding the slightly reduced biomass observed in genome-integrated strains, although chromosomal integration reduces plasmid burden, locus-dependent effects on gene expression may occur. In addition, lower expression levels or pathway imbalance may lead to accumulation of inhibitory intermediates, modestly affecting growth. The observed biomass differences were relatively minor and remained within a typical biological variation range.

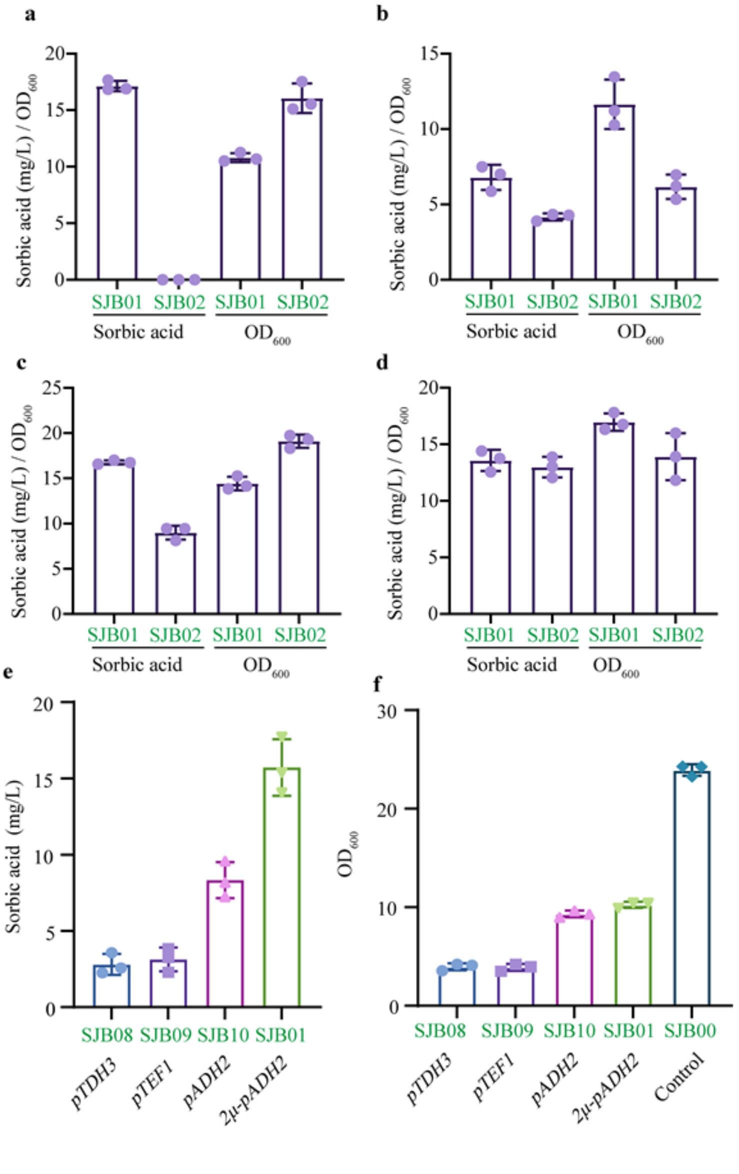

Fig. S20. Influence of different promoters and integration strategies on SA production.

These clarifications and additional experimental details have been incorporated into the revised manuscript (Line **311-316**).

4. Section "Linker-mediated enzyme fusion fails to improve SA production" appears

minimally impactful in its current form. Consider discussing it briefly within another relevant section rather than devoting a full section to it.

Response:

We thank the reviewer for this constructive suggestion. In the revised manuscript, this content has been integrated into the preceding section (**Line 348-356**) and presented in a more concise form. The standalone subsection has been removed to improve structural coherence and narrative flow.

5. In section “Engineering ethanol utilization for enhanced acetyl-CoA and NADPH supply”, ALD6 is a key NADPH-regenerating enzyme in the cytosol; therefore, the observed production improvement could also relate to enhanced NADPH supply. Furthermore, after overexpression of Zwf1, Gnd1, Tkl1, and Tal1, confirming the change in NADPH levels would support the authors’ conclusion.

Response:

We thank the reviewer for this insightful comment. We agree that ALD6 contributes to cytosolic NADPH regeneration and could potentially influence SA production through redox balance.

To directly address this point, we quantified intracellular NADPH levels in strains overexpressing ALD6 (SJB27), ZWF1 (SJB37), PGI1 (SJB38), and AsTAL1/AsTKL1 (SJB39). We have newly added **Fig. S23d** in the revised manuscript to present the time-course analysis of intracellular NADPH levels in these engineered strains. These modifications led to a transient increase in NADPH levels, particularly at 24 h, whereas by 48 h NADPH levels remained only slightly higher than those of the control strain (SJB00).

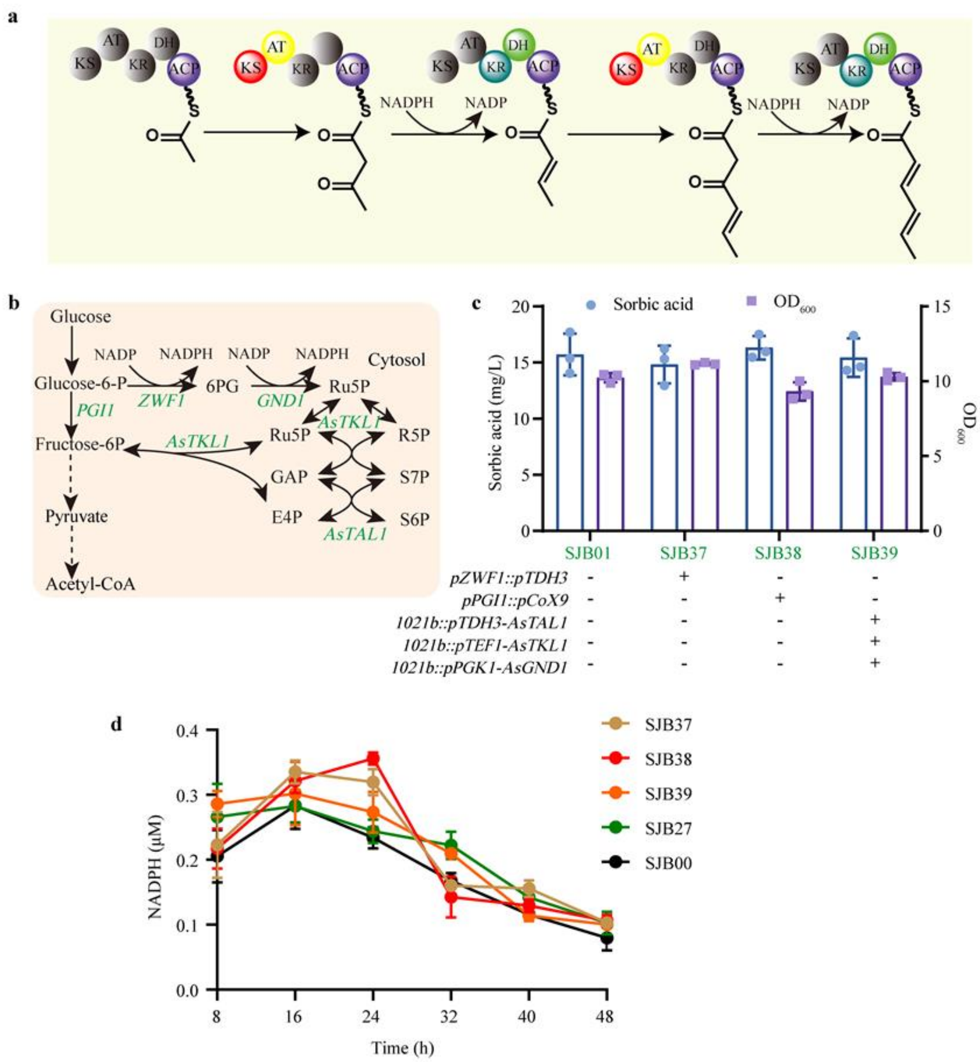

Fig. S23. Engineering NADPH supply and central carbon metabolism for SA production.

Given that SA biosynthesis consumes only two NADPH molecules per product molecule, these data suggest that endogenous NADPH supply is sufficient under our experimental conditions and is unlikely to be the limiting factor for SA production. Instead, the observed improvement is more consistent with enhanced acetyl-CoA availability.

Corresponding clarifications have been incorporated into the revised manuscript (**Line 409–410** and **417–422**).

6. In the description "In shake-flask fermentations, SJB51 exhibited optimal growth at pH 6.0 (OD_{600} = 13.8), with SA titers reaching 305.3 mg/L. SA production increased with higher pH values and peaked at pH 7.0 (340 mg/L), although biomass decreased slightly (OD_{600} = 12.3)." Please clarify how pH stability was maintained during the shake-flask fermentations.

Response:

We thank the reviewer for this important comment. We agree that pH stability is critical for accurate interpretation of shake-flask fermentation results.

Prior to inoculation, the initial pH of the medium was adjusted to the desired value. During fermentation, pH was monitored every 12 h, and deviations were corrected by the addition of 2 M NaOH or 2% (v/v) H_2SO_4 to maintain the culture within the target pH range throughout the cultivation period.

This clarification has been incorporated into the revised manuscript (**Line 486–487**) and reflected in the legend of **Fig. 5**.

7. Could the authors comment on the commercial potential of this bio-manufacturing approach as an alternative to other production routes in terms of cost?

Response:

We thank the reviewer and the editorial team for highlighting the importance of providing a economic comparison.

In the revised manuscript, we have added a dedicated paragraph (**Line 613-629**) in the Discussion section benchmarking our biosynthetic route against established production pathways. We referenced recent techno-economic analysis of TAL-derived KS production (Green Chem. 27, 6087-6104, 2025) to provide a quantitative comparison framework, included prevailing market price ranges for SA/KS, and (iii) incorporated the theoretical maximum yield (0.41 g/g from glucose to SA via an PKS route) to contextualize feedstock efficiency and carbon utilization.

We explicitly acknowledge that the current titer achieved in this study (1.84 g/L at 50 L scale) represents an early-stage demonstration and remains below the levels typically required for large-scale commercialization of commodity organic acids. The increase from the initial shake-flask level (15 mg/L) reflects the scalability and engineering responsiveness of the system. We further outline coordinated metabolic and process-level improvements—such as enhanced carbon flux, acid tolerance engineering, and in situ product removal strategies—that would be necessary to advance economic feasibility.

To ensure consistency, we have also revised the Conclusion section (**Line 637-643**) to reflect this realistic positioning and to avoid overstating the current industrial competitiveness of the system. For consistency, the final sentence of the Abstract has been slightly revised to align with this balanced commercial assessment and to avoid overstating the current industrial competitiveness of the system.

We believe these additions provide a balanced and quantitative assessment of the commercial outlook while maintaining appropriate expectations for this stage of development.

8. There are some writing mistakes:

In the sentence “The term “sorbic acid” traces its origin to its first isolation from the unripe berries of the mountain ash tree (Sorbus aucuparia) by A. W. Hoffman in the 18597. and then was commercially used as antimicrobial additive in the 1940s.” Replace the full stop in the original text ("18597. and then") with a comma after 1859.

In the sentence “It is challenging in overproduction of commercially valuable antimicrobial agent SA in its natural host Myrothecium sp. FJNU6, since SoaC effiecnt driven the bioconversion of SA toward SN biosynthesis.” Effiecnt should be efficiently

Response:

We thank the reviewer for carefully identifying these writing errors. The full stop after “1859” has been corrected to a comma as suggested. In addition, “effiecnt” has been corrected to “efficiently” in the revised manuscript. We appreciate the reviewer’s attention to detail.
